# 11-deoxycortisol positively correlates with T cell immune traits in physiological conditions

**DOI:** 10.1016/j.ebiom.2023.104935

**Published:** 2023-12-21

**Authors:** Chunying Peng, Xun Jiang, Martin Jaeger, Pepijn van Houten, Antonius E. van Herwaarden, Valerie A.C.M. Koeken, Simone J.C.F.M. Moorlag, Vera P. Mourits, Heidi Lemmers, Helga Dijkstra, Hans J.P.M. Koenen, Irma Joosten, Bram van Cranenbroek, Yang Li, Leo A.B. Joosten, Mihai G. Netea, Romana T. Netea-Maier, Cheng-Jian Xu

**Affiliations:** a Division of Endocrinology, Department of Internal Medicine, Radboud University Medical Center, Nijmegen, the Netherlands; b Centre for Individualised Infection Medicine (CiiM), A Joint Venture Between the Helmholtz-Centre for Infection Research (HZI) and Hannover Medical School (MHH), Hannover, Germany; c TWINCORE, Centre for Experimental and Clinical Infection Research, A Joint Venture Between the Helmholtz-Centre for Infection Research (HZI) and Hannover Medical School (MHH), Hannover, Germany; d Department of Internal Medicine and Radboud Center for Infectious Diseases, Radboud University Medical Center, Nijmegen, the Netherlands; e Department of Laboratory Medicine, Radboud University Medical Center, Nijmegen, the Netherlands; f Research Centre Innovations in Care, Rotterdam University of Applied Science, Rotterdam, the Netherlands; g Laboratory Medical Immunology, Radboud University Medical Center, Nijmegen, the Netherlands; h Department of Medical Genetics, Iuliu Hatieganu University of Medicine and Pharmacy, Cluj-Napoca, Romania

**Keywords:** Immune homeostasis, Steroid hormones, 11-deoxycortisol, T cell proliferation, Th17

## Abstract

**Background:**

Endogenous steroid hormones have significant effects on inflammatory and immune processes, but the immunological activities of steroidogenesis precursors remain largely unexplored.

**Methods:**

We conducted a systematic approach to examine the association between steroid hormones profile and immune traits in a cohort of 534 healthy volunteers. Serum concentrations of steroid hormones and their precursors (cortisol, progesterone, testosterone, androstenedione, 11-deoxycortisol and 17-OH progesterone) were determined by liquid chromatography-tandem mass spectrometry. Immune traits were evaluated by quantifying cellular composition of the circulating immune system and *ex vivo* cytokine responses elicited by major human pathogens and microbial ligands. An independent cohort of 321 individuals was used for validation, followed by *in vitro* validation experiments.

**Findings:**

We observed a positive association between 11-deoxycortisol and lymphoid cellular subsets numbers and function (especially IL-17 response). The association with lymphoid cellularity was validated in an independent validation cohort. *In vitro* experiments showed that, as compared to androstenedione and 17-OH progesterone, 11-deoxycortisol promoted T cell proliferation and *Candida*-induced Th17 polarization at physiologically relevant concentrations. Functionally, 11-deoxycortisol-treated T cells displayed a more activated phenotype (PD-L1^high^ CD25^high^ CD62L^low^ CD127^low^) in response to CD3/CD28 co-stimulation, and downregulated expression of T-bet nuclear transcription factor.

**Interpretation:**

Our findings suggest a positive association between 11-deoxycortisol and T-cell function under physiological conditions. Further investigation is needed to explore the potential mechanisms and clinical implications.

**Funding:**

Found in acknowledgements.

**Research in context**

**Evidence before this study**

In this study, we aim to evaluate the immunological effects of the upstream precursors of cortisol in physiological conditions. We searched PubMed without any language restriction using combinations of the terms “steroidogenesis intermediates”, “11-deoxycortisol”, “17-hydroxyprogesterone”, “androstenedione”, “lymphocyte” and “immune”, and mainly focused on human studies. The cortisol synthetic precursors were mainly used as diagnostic markers of steroidogenesis disorders, their intrinsic biological activities have rarely been investigated. We did not find studies relating immune functionality with the concentrations of steroid precursors mentioned above in humans.

**Added value of this study**

The current study provided a comprehensive assessment of the steroid hormones and their precursors in two independent cohorts of healthy volunteers, in combination with systematic immunophenotyping. Our data demonstrated that 11-deoxycortisol concentration positively correlated with peripheral lymphoid immune cell counts and cytokine responses, and *in vitro* experiments further support the immunoregulating property of 11-deoxycortisol.

**Implications of all the available evidence**

Steroids-disbalance is common in different clinical scenarios including both endogenous and iatrogenic causes. Findings from this study suggested a previously unknown immunomodulatory property of 11-deoxycortisol, which could be one of the pathogenic mechanisms underlying the immunological complications. It would be of interest to investigate how such mechanisms could be therapeutically targeted to restore immune homeostasis.

## Introduction

Variation in immune responses is an important factor that influences the susceptibility to immune-related diseases.^1^ The adaptive immune system develops significantly during infancy and early childhood as a result of continuous exposure to diverse antigens. In adulthood, the diversity and the size of the immune cell pool reach a plateau and remain stable over time.^2^ The endocrine system is one of the most effective tools utilized by the central nervous system to integrate and synchronize the immune response with a myriad of physiological process.^3^ Under stressed conditions (e.g., infection), the immune system detects the damage signals and elicits a cascade of defensive events, while the hypothalamus-pituitary-adrenal (HPA) axis is paramount to restore immune homeostasis via the release of steroid hormones which function as a crucial messenger between the central regulator and peripheral immunity. Cortisol, the final effector hormone of the HPA axis, apart from its well-known immunosuppressive effects in stressed conditions, it has been reported to increase the organism's immunological fitness via coordination of the immune cell selection and differentiation in thymus, and of the circadian oscillations of the number of circulating T cells in steady-state conditions.^4^^,^^5^ In steroidogenesis-driven diseases, both excessive and insufficient cortisol production have been reported to be associated with disturbed immune homeostasis, mainly manifested as dysregulation of immune cell compositions and increased risk of infections.^6^ In pathological conditions, a dysregulated HPA axis might exacerbate the immunological disturbances via T cell overactivation and depletion, tipping the balance between conventional and regulatory T cells and induce disbalances in inflammatory immune responses.^7^, ^8^, ^9^

The canonical steroidogenesis pathway involves a series of enzymes, with the first and rate-limiting step being conversion of cholesterol into pregnenolone. In the zona fasciculata cells of the adrenal cortex, pregnenolone is further converted into 17-OH pregnenolone, 17-OH progesterone (17-OHP), 11-deoxycortisol and, eventually, cortisol which is rapidly released into circulation.^10^ Additionally, the upstream steroid precursors can be diverted towards the synthesis of sex hormones (progesterone, testosterone and androstenedione) via shared precursors.^11^ For a long time, steroidogenic intermediates have been considered less bioactive and their role in health and disease remained largely unexplored.

Congenital adrenal hyperplasia (CAH) is a condition caused by various enzyme deficiencies in the steroidogenic pathway. Some forms of CAH are characterized by cortisol deficiency and accumulation of upstream steroid precursors, requiring life-long cortisol replacement therapy.^12^ Little is known about the immune function of patients with CAH. One recent case–control study indicated however that the infection risk in untreated patients with CAH did not differ from matched controls, indicative of a relative intact immune system. On the contrary, those who receiving cortisol replacement therapy, which also can result in suppression of upstream steroidogenic intermediates, showed increased risks of urinary tract and lower respiratory tract infections.^13^ More interestingly, a recent study has reported a unique cohort of severely affected and untreated patients with CAH from Indonesia.^14^ These patients had biochemically confirmed inadequate cortisol production and significantly elevated steroidogenic intermediates in circulation. The most striking finding was that they presented no clinical signs of cortisol deficiency, thus leading to our hypothesis that steroidal intermediates have non-redundant biological activities that contribute to immune homeostasis.

In this study, our objective was to conduct a comprehensive investigation into the potential immunoregulatory effects of cortisol and its precursors in two large, independent cohorts of healthy individuals and to explore the underlying mechanisms of these effects. To achieve this, we assessed the profile of peripheral steroidal precursors and their association to immune traits. Furthermore, we conducted *in vitro* functional validation to examine the correlations observed between steroidogenesis intermediates and T cell function.

## Methods

### Study design

**Fig. 1:**
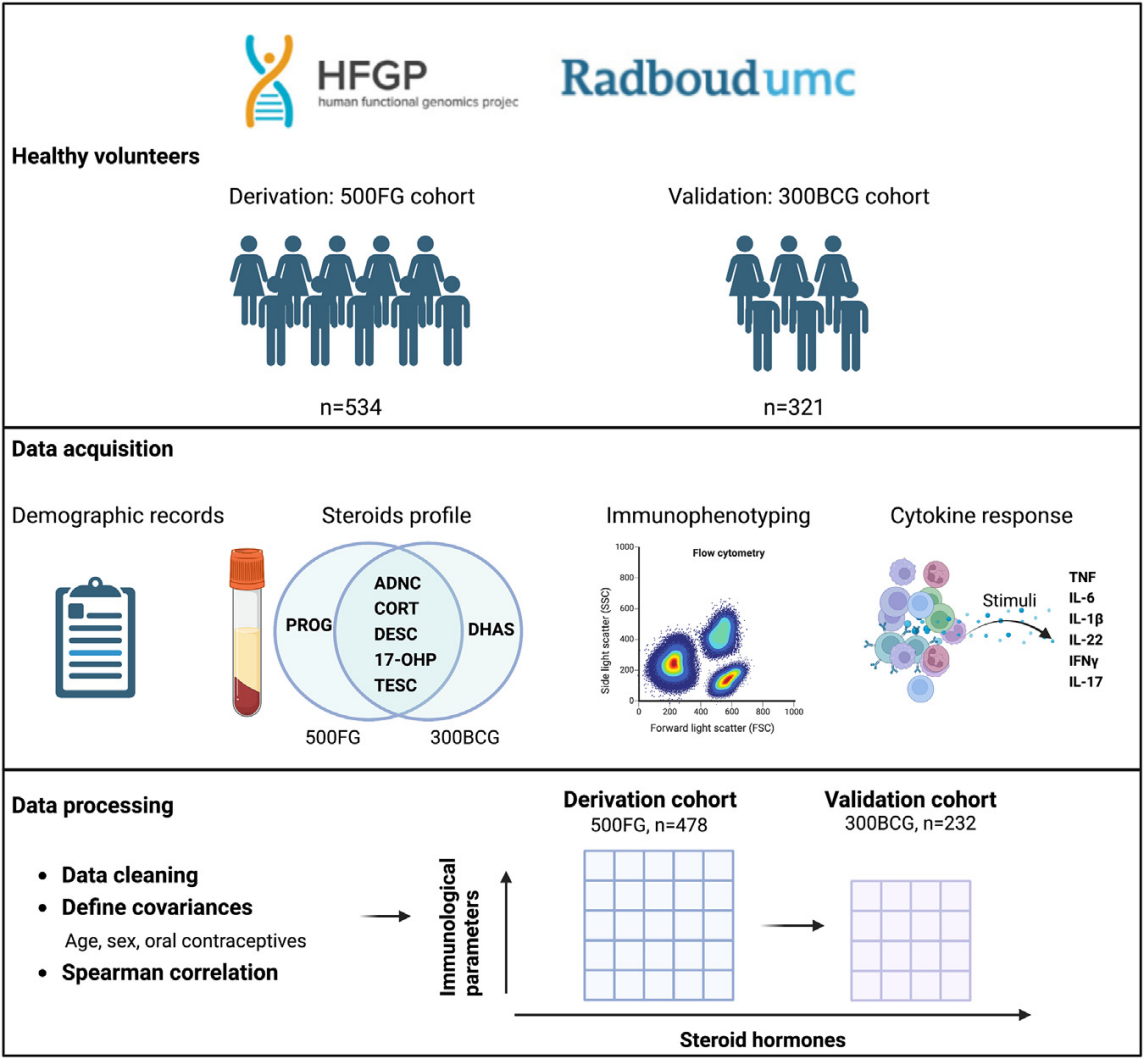
A schematic diagram of study design. ADNC, androstenedione; CORC, cortisol; DESC, 11-deoxycortisol; DHAS, dehydroepiandrosterone sulfate; 17-OHP, 17-hydroxyprogesterone; PORG, progesterone; TESC, testosterone.

The schematic diagram of the study procedures is presented in Fig. 1. Two cohorts of healthy individuals were included, namely the discovery cohort and the validation cohort. In both cohorts, the steroid profile and its association with immune phenotyping and cytokine production capacity were assessed.

### Discovery cohort-500FG

The discovery cohort (500FG) consisted of 534 healthy individuals of Western-European ancestry recruited between August 2013 and December 2014 at the Radboud University Medical Center, the Netherlands (for inclusion criteria and further details, refer to Human Functional Genomics Project (HFGP)).^15^ The primary objective of the HFGP was to assess the endogenous and exogenous sources of variation in immune responses. Demographic data were collected, and blood samples were taken in the morning.

### Validation cohort-300BCG

The validation cohort consisted of 321 healthy adult volunteers of Western European ancestry who were enrolled in the 300BCG cohort between April 2017 and June 2018 in the Radboud University Medical Center, the Netherlands.^16^ The inclusion and exclusion criteria were described previously.^17^ This cohort was designed to investigate the effects of BCG vaccination on immune function, thus morning blood samples collected prior to BCG vaccination were used as the validation set in our study.

### Measurement of steroid hormone levels

Serum cortisol, 11-deoxycortisol, androstenedione, 17-OHP, testosterone and progesterone were analyzed by liquid chromatography-tandem mass spectrometry after protein precipitation and solid-phase extraction as previously described.^15^ Female participants taking oral contraceptive pills (OCP) in the 300BCG cohort were excluded from steroid profiling. Progesterone was not measured in the 300BCG cohort.

### Analysis of immune traits

Immunophenotyping of whole blood or peripheral blood mononuclear cells (PBMCs) was performed by flow cytometry (Beckman Coulter, Indianapolis, USA). Cells were processed immediately after blood sampling and typically analyzed within 2–3 h to minimize biological variability. Manually gating and data processing were performed with Kaluza software version 1.3 (Beckman Coulter).^15^ The 500FG cohort identified 73 distinct cellular subpopulations. The absolute number of white blood cells per ml of blood determined by the cell counter (Beckman Coulter) was used to calculate the absolute numbers of CD45^+^ leukocyte subsets as measured by flow cytometry, which was utilized for further analysis. The immunological parameters obtained from the 300BCG cohort were similar to those of the 500FG cohort, except that 6 out of 73 cellular subsets were not determined. The full list of immunological parameters is provided in the Supplementary data (Supplementary Table S1).

### Ex vivo stimulation and cytokine measurement

Heat-killed human pathogens and microbial ligands were used for *ex vivo* stimulation. Isolation of PBMCs from fresh blood was performed with gradient centrifugation as previously described. Cells were resuspended in RPMI-1640 complete culture medium. PBMC stimulations were performed with 5 × 10^5^ cells/well in round-bottom 96-well plates (Greiner) for either 24 h or 7 days in the presence of 10% human pooled serum (HPS) at 37 °C and 5% CO_2_. Whole blood was stimulated for 48 h. Supernatants were collected and stored at −20 °C until cytokine measurement. Concentrations of Interleukin (IL)-1β, IL-6, tumor necrosis factor (TNF)-α, IL-17, IL-22 and interferon (IFN)-γ were measured following the manufacturer's protocols (PeliKine Compact or R&D Systems). The full list of cytokines, stimuli, cell systems and incubation times is provided in Supplementary data (Supplementary Table S2).

### In vitro cell culture

PBMCs isolation from buffy coats obtained from the Sanquin bloodbank, Nijmegen, The Netherlands, was performed with density gradient centrifugation as previously described. 11-deoxycortisol methanolic stock, 17-OHP methanolic stock and androstenedione acetonitrile stock (Sigma–Aldrich) was diluted with sterile PBS. Conditions with the same concentration of methanol or acetonitrile were used as vehicle control. The working concentrations of 11-deoxycortisol, 17-OHP and androstenedione *in vitro* were based on the total serum concentrations from both healthy cohorts, ranging from supra-physiological (10 nM) to sub-physiological level (10^−3^ nM). In selected experiments, HPS was incubated with dextran-coated charcoal at 4 °C overnight, followed by centrifugation at 2500*g* for 15 min. Supernatant was passed through 0.22 μm filter and stored at −80 °C (steroid-stripped HPS). HPS that incubate overnight without dextran-coated charcoal was used as control. PBMCs were stimulated with LPS (10 ng/ml) and heat-inactivated *C. albicans* (strain ATCC MYA-3573, UC 820, 1 × 10^6^/ml).

### T cell proliferation

PBMCs were labeled with CellTrace violet (Invitrogen) and plated at 1.5 × 10^5^ cells per 100 μl with anti-CD3/CD28 dynabeads (GIBCO, cell to bead ratio was 2:1) in a 96-well round-bottom plate in the presence of human recombinant IL-2 (100IU/ml, Biolegend) and 10% HPS. Unstimulated PBMCs were plated the same way but in the absence of anti-CD3/CD28 dynabeads. 11-deoxycortisol, 17-OHP and androstenedione stock with indicated concentration was added into culture medium before T cell activation. All stimulated/unstimulated, treated/untreated cells were incubated together for 4 days at 37 °C, 5% CO_2_. Supernatants were collected for the measurement of IFN-γ and TNF-α. Cells were collected and stained with fixable viability dye (FVD) eFlour 780 (Invitrogen), AF700-anti-CD3, BV650-anti-CD4, BV785-anti-CD8, PECy7-anti-CD25, PE-anti-CD127, FITC-anti-CD62L, BV785-anti-CXCR3, APC-anti-PD-L1 and PE-anti-PD antibodies in separate panels. Following staining, cells were resuspended in 200 μl of FACS buffer (1% BSA in PBS) and measured with Cytoflex (Beckman Coulter Inc.). Data were analyzed using the proliferation platform in FlowJo v10.7.1 software (BD Bioscience). Four indexes generated by FlowJo, Expansion index (fold-expansion of the overall culture), Division index (average number of cell divisions that a cell in the original population has undergone), Proliferation index ((total number of divisions)/(number of divided cells)) and Replication index (fold-expansion of only the responding cells), were compared in statistical analysis. The gating strategy used in the experiment is shown in Supplementary Fig. S3. The antibody information and RRID are provided in Supplementary Table S4.

### Intracellular cytokine and nuclear receptor analysis

After 7-day priming with heat-killed *C. albicans* (1 × 10^6^/ml), PBMCs were stimulated with Ionomycin (1 μg/ml) and PMA (50 ng/ml) in the presence of Golgi plug (1 μl/ml) for 4 h. Cells were collected in cold PBS and followed by FVD and surface marker staining (BV510-anti-CD8, BV650-anti-CD4 and AF700-anti-CD3 antibodies). Cells were fixed and permeabilized with FIX & PERM kit according to manufacturer's instructions, after which, cells were stained with FITC-anti-IL-17, PECy7-anti-IFN-γ, PerCP/Cy5.5-anti-T-bet and PE-CF594-anti-RORγt antibodies in permeabilization buffer at RT for 45min. The gating strategy used in the experiment is shown in Supplementary Fig. S6a. The antibody information and RRID are provided in Supplementary Table S4.

### Statistical analysis

The statistical analysis was conducted using R Statistical Software (version 4.2.0). The ‘Spearman’ method was used to estimate the correlation between steroid hormones, host characteristics and cell counts. OCP significantly affects serum total cortisol concentration by upregulating cortisol-binding globulin (CBG).^18^ In the 500FG cohort, this was addressed by either excluding females taking OCP or statistical correction. In the 300BCG cohort the women using OCP were excluded, so that no correction for use of OCP was required. The Spearman associated p-values were used to indicate the rank-based measure of correlation, positive and negative correlations were presented in different colors. Cell counts were normalized with inverse rank transformation algorithm. For p-value adjustment for multiple testing, the FDR method was used. Missing data were excluded from the correlation analysis. All plots were generated by R-package ggplot2 (version 3.3.6). Given the exploratory nature of the study, a sample size could not be calculated prior to the study. Two independent large cohorts were included to assess whether findings in the discovery cohort are reproducible in the validation cohort, in order to exclude false positive results.

For *in vitro* experiments, Wilcoxon test was performed to compare means or medians. Data analyses and visualization were performed with R-package ggplot2 (version 3.3.6). p-values of 0.05 or less were considered as statistically significant.

### Study approval

The 500FG was approved by the Arnhem-Nijmegen Medical Ethical Committee (no. NL42561.091.12). The 300BCG study was approved by the Arnhem-Nijmegen Medical Ethical Committee (no. NL58553.091.16). Experiments were conducted according to the principles expressed in the Declaration of Helsinki. All patients provided informed consent before the inclusion.

### Role of the funding source

The funders had no roles in study design, data collection and analysis or preparation of the manuscript.

## Results

### Demographic characteristics and steroids profile of the discovery and validation cohorts

A total of 534 healthy volunteers of European ancestry were recruited in the 500FG cohort. After quality controls and exclusion of a number of volunteers due to medication use or genetic background (non-European ancestry), steroid hormones data were available for 482 individuals. Four outliers were detected and excluded in the data cleaning step, and 478 participants (270 female, 208 male) were included for analysis. The 300BCG validation set consist of 321 healthy volunteers, steroid hormones were not determined in females receiving OCP (n = 89), thus leaving a total of 232 (93 female, 139 male) individuals to be included for further analysis. The demographic characteristics of the 500FG and 300BCG cohorts were comparable (Table 1).

**Table 1:** Comparable demographic characteristics of the derivation & validation cohort.

Clinical variables	Derivation		Validation	p-value
	500FG whole	500FG Female	300BCG	
n	478	266	232	
Sex (% male)	43.5	–	59.9	
Age (years)	23 (21–27)	22 (20–26)	23 (21–26)	p = 0.4480
BMI	22.22 (20.75–24.29)	21.56 (20.48–23.58)	22.21 (20.88–23.86)	p = 0.9499
OCP (%)	–	53.7	–	

Data are represented as Median (25th −75th percentile), age and BMI (500FG whole versus 300BCG) were compared by non-parametric Mann–Whitney test.

For the 500FG cohort, steroids data were available in 482 individuals, 4 outliers were excluded, 478 were included for analysis. Within the 500FG female subset, there were 270 female participants in total, 4 missing oral contraceptive information.

For the 300BCG cohort, steroids data were not available in females taking oral contraceptive pills, 232 individuals were included for analysis. OCP, oral contraceptive pills.

**Fig. 2:**
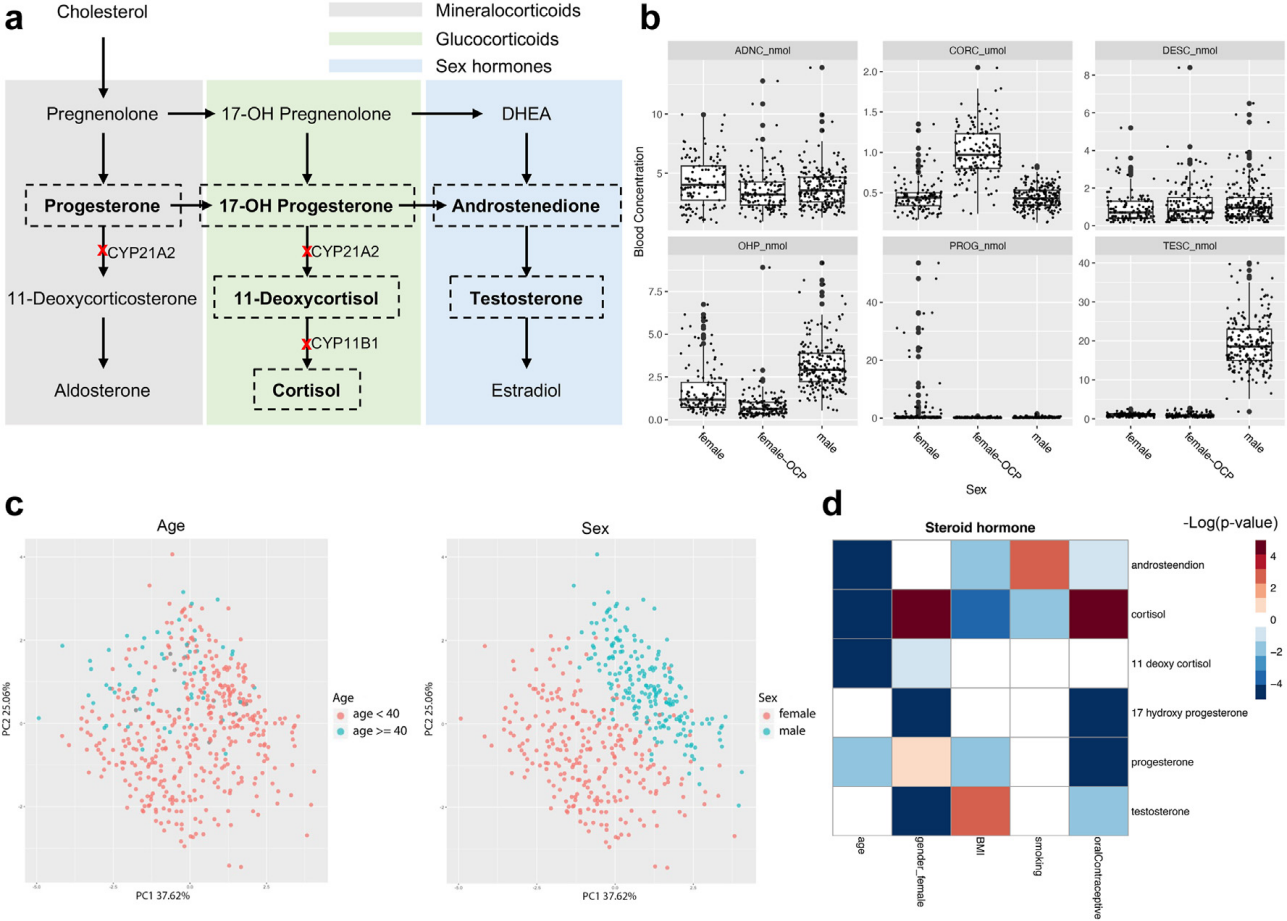
Relation between steroid hormones with non-genetic host characteristics. (a) Schematic of adrenal steroidogenesis pathway. Steroids of interest are highlighted with dash frames. Deficiencies in CYP11B1 and CYP21A2 genes are the common causes of congenital adrenal hyperplasia, which lead to the accumulation of precursors prior to the blocked step. Background color indicates adrenal cortex zone-specific steroids synthesis pathways. Grey area shows the synthesis of mineralocorticoids in zona glomerulosa. Green area shows the synthesis of glucocorticoids in zona fasciculata. Blue area shows the synthesis of sex hormones in zona reticularis. (b) Box plots of the six steroid hormones determined in the 500FG cohort. Male (n = 208), female (n = 121), female-OCP (n = 145). Each dot represents one healthy volunteer, outliers are shown as large black dots. (c) Global visualization of Steroid hormone of individuals using two top principal components. Age group and sex are labeled in different colors. (d) A heatmap showing the correlation between different steroid hormone and non-genetic host characteristics in the 500FG cohort. Negative and positive correlations were presented in different colors. Correlations which did not reached statistical significance were shown as blank. CYP11B1, cytochrome P450 family 11 subfamily B member 1; CYP21A2, cytochrome P450 family 21 subfamily A member 2; DHEA, Dehydroepiandrosterone; Female: females not taking oral contraceptives; female-OCP: females taking oral contraceptive pill; OHP, 17-hydroxyprogesterone.

The steroids of interest are shown in Fig. 2a, in the context of the steroidogenesis pathways in the adrenal cortex. Detailed descriptions of all the steroids determined in both cohorts and the correlation among them are provided in Supplementary Table S3 and Fig. S1. Previous studies, including our own, have reported that non-genetic host characteristics, especially, age and sex, are the primary contributors to the immunological variation.^2^^,^^15^ To investigate whether similar patterns exist within the steroids’ variation, we examined the relationship between steroid hormones and non-genetic host characteristics, including age, sex, BMI and the use of oral contraceptives. We found that cortisol concentration was significantly higher in females taking OCP from the 500FG cohort (p < 0.0001). 11-deoxycortisol, 17-OHP and testosterone concentrations were higher in male participants while androstenedione concentration was higher in females. No sex difference in cortisol was observed (Fig. 2b). Principal component analysis of the 500FG dataset clearly captured the effects of age and sex on the variance of the steroid profile (Fig. 2c). Similarly, global correlation highlighted the predominant impact of host characteristics on the serum steroid landscape (Fig. 2d), which was addressed in the following data analysis.

### Positive correlation of 11-deoxycortisol to adaptive immune traits in the derivation cohort

#### Analytic strategy

**Fig. 3:**
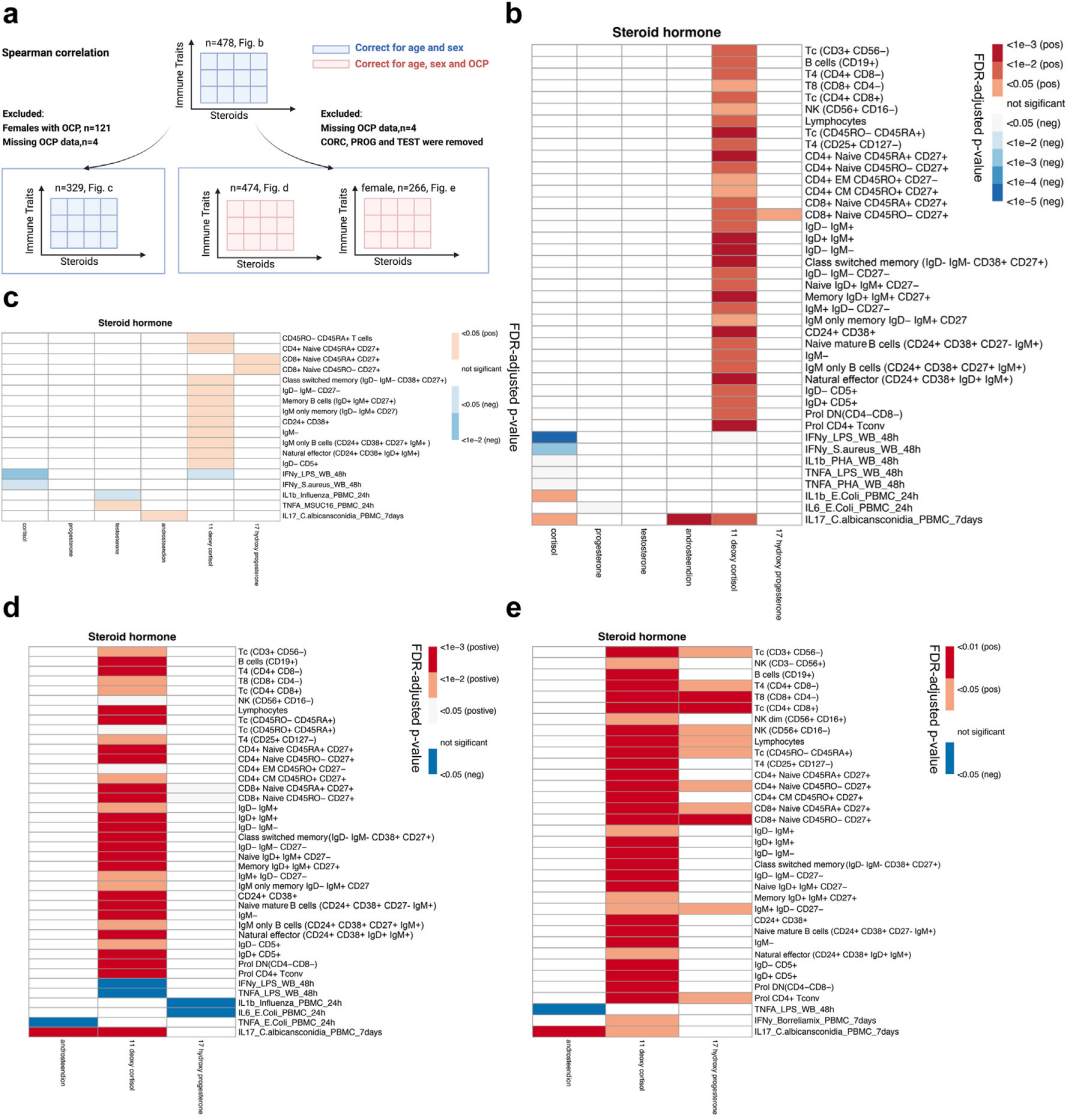
Correlation of steroid hormones to adaptive immune traits in 500FG cohort. (a) Workflow of data analysis. (b) Spearman correlation of steroids to immune traits, all individuals included (n = 478), age and sex adjusted; (c) Spearman correlation of steroids to immune traits, females taking oral contraceptives were excluded (n = 329), age and sex adjusted. (d) Spearman correlation of steroidogenesis intermediates to immune traits, all individuals except 4 missing oral contraceptive information were included (n = 474), age, sex and oral contraceptive use adjusted. (e) Spearman correlation of steroidogenesis intermediates to immune traits, all females except 4 missing oral contraceptive information were included (n = 266), age, sex and oral contraceptive use adjusted, p-values were FDR-adjusted, correlations that did not reach statistical significance were shown as blank, colors indicate positive or negative correlation. CM, central memory; CpG, short synthetic single-stranded DNA molecules containing unmethylated CpG dinucleotides; DN, double negative T cells; Eff, effector; EM, effector memory; MSUC16, combination of monosodium urate crystals (MSU) and palmitic acid (C16:0); MTB, *Mycobacterium tuberculosis*; neg, negative; OCP, oral contraceptive pills; PHA, phytohemagglutinin; pos, positive; Prol, proliferating; T4, CD4^+^ T cells; T8, CD8^+^ T cells; Tc, T cells; Tconv, conventional T cells; Treg, T regulatory cells; WB, whole blood.

We first tested for associations between steroid hormones and cell counts of peripheral immune cell subpopulations of the full dataset of the discovery cohort (n = 478). After correcting for age and sex, we observed a highly statistically significant positive correlation between 11-deoxycortisol and lymphoid cell populations and their function (Fig. 3b), while no similar correlation to myeloid cell immune traits was observed. To remove the influence of the use of OCP on steroid profiles, two approaches were used. Firstly, we performed similar analysis after excluding females receiving OCP (n = 329) (Fig. 3c). Secondly, we excluded hormones most affected by OCP and sex (cortisol, progesterone and testosterone) and performed the correlation analysis only with the steroidogenesis intermediates (11-deoxycortisol, 17-OHP and androstenedione), the use of OCP was used as covariance and adjusted statistically (Fig. 3d). Four individuals missing OCP data were excluded from analysis. In both approaches, 11-deoxycortisol remained as the prime steroid associated with lymphoid immune traits, yet other steroids, especially 17-OHP (the direct precursor of 11-deoxycortisol), showed similar trends when the influence of OCP was removed. Conversely, sex hormones (progesterone and testosterone) remained as the least relevant steroids regarding immune traits, possibly due to the influence of divergent gonadal hormones.

#### Cellular subsets

For the phenotyping of T cells, CD27 and CD45RA were utilized for distinguishing naïve, memory and effector cells. Naïve cells co-express both markers, while effector and memory cells only express CD45RA or CD27, respectively.^19^ We observed that 11-deoxycortisol mainly correlated with the naïve (both CD4^+^ and CD8^+^) and memory CD4^+^ T cells populations, but not with the effector subsets. B cell subsets were discriminated by CD19/CD20, and the maturation stages of CD19^+^ B cells were further assessed by the expression of IgM/IgD and/or CD24/CD38.^20^^,^^21^ Similar to T cells, CD27^-^ naïve and CD27^+^ memory B cells were the most represented cellular subsets positively associated with 11-deoxycortisol concentration, while plasma cells (CD19^+^CD20^-^) and their precursors (plasma blast cells) were not. Natural killer cells (CD56^+^CD16^-^), a type of lymphocytes that display effector functions of innate immunity, were consistently correlated to 11-deoxycortisol concentration through different analytic strategies.

#### Cytokine responses

Immune responsiveness was evaluated by cytokine production in multiple stimulation conditions. The PBMC system assesses the function of monocytes and lymphocytes, while the whole blood system reflects the interplay of the total leukocyte population (e.g., polymorphonuclear cells such as neutrophils, eosinophils and basophils) and platelets in the presence of autologous plasma.^22^ The negative association of cortisol and 11-deoxycortisol with the whole blood cytokine response was intriguing, particularly concerning the 48 h IFN-γ production upon LPS challenge. However, no such correlation was observed in the PBMC stimulation system, possibly indicating the involvement of the polymorphonuclear cells, platelets or other plasma mediators in this effect. To investigate pathogen-specific cytokine responses, various major human pathogens (bacteria, fungi and viruses) and microbial ligands were tested *ex vivo*. The most consistent finding was the positive correlation between *C. albicans*-elicited 7-day IL-17 production and the steroidogenesis intermediates, namely, 11-deoxycortisol and androstenedione. It should be noted that our previous research indicated higher IL-17 responses in women.^23^ For this reason, we repeated the analysis in the female subgroup (Fig. 3e). In this sub-analysis, 11-deoxycortisol remained the most strongly related steroid to lymphoid cellularity and IL-17 response. Moreover, additional associations of 17-OHP to immune traits were found in the female group.

In summary, these highly consistent results obtained by different analytic strategies are indicative of significant association between 11-deoxycortisol and the numbers and function of lymphoid cells.

### Replication in the 300BCG cohort

**Fig. 4:**
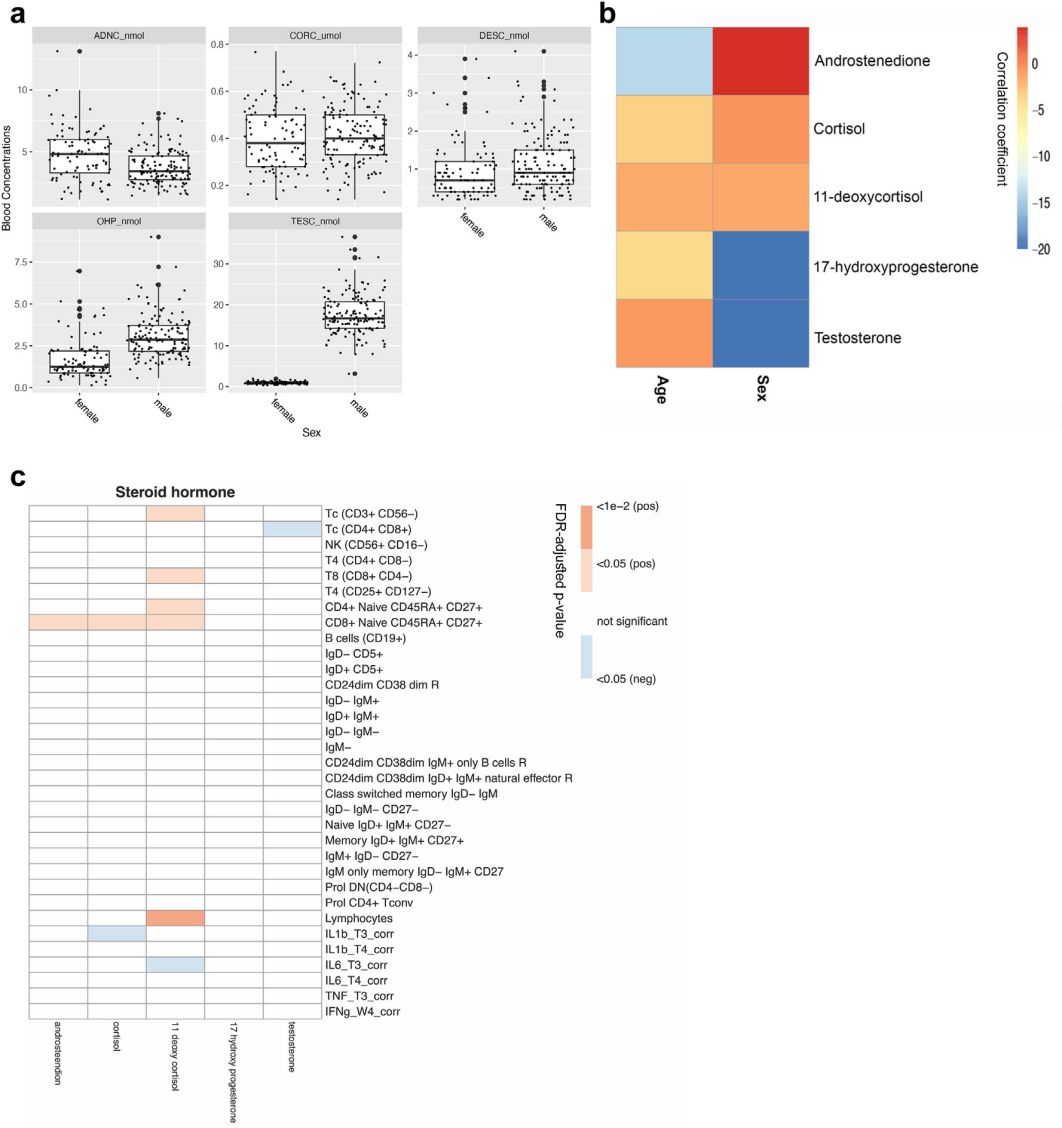
Replication in 300BCG cohort. (a) Box plot of the five steroid hormones determined in the 300BCG cohort, male (n = 139), female (n = 93). Each dot represents one healthy volunteer, outliers are shown as large black dots. (b) A heatmap summarizing the correlation between different steroid hormone and non-genetic host characteristics in the 300BCG cohort (n = 232), color indicates correlation coefficients. (c) Correlation of immune traits to testosterone, cortisol, androstenedione, 11-deoxycortisol, 17 hydroxy progesterone in the 300BCG cohort, after correcting age and sex. FDR adjusted p-value is used to show the significance of correlation, correlations that did not reach statistical significance were shown as blank, colors indicate positive or negative correlation. Corr, log10 transformed and corrected for batch; T3_corr, PBMCs with *S. aureus* stimulation for 24 h; T4_corr, PBMCs with *M. tuberculosis* stimulation for 24 h; W4_corr, PBMCs with *M. tuberculosis* stimulation for 7 days.

To assess the robustness of the association between 11-deoxycortisol and T cell functions, a validation analysis was performed in an independent cohort (300BCG). Similar to the results obtained from the 500FG, the majority of steroid hormones in the 300BCG displayed significant sex difference and negatively correlated with age (Fig. 4a and b). After correcting for age and sex, we found that the most consistent 11-deoxycortisol-correlated immune traits were total lymphocytes, total T cells (CD3^+^CD56^-^), CD8^+^ T cells (CD8^+^CD4^-^), CD4 naïve cells (CD4^+^CD45RA^+^CD27^+^) and CD8 naïve cells (CD8^+^CD45RA^+^CD27^+^) (Fig. 4c). The magnitude of the associations was less prominent than that in the discovery cohort, likely due to the smaller sample size and reduced statistical power. However, when FDR adjustment was not applied, the statistically significant correlations largely recapitulated those of the 500FG cohort (Supplementary Fig. S2). When we repeated the analysis only in the female subgroup (n = 93), none of the associations reached statistical significance, possibly due to the smaller sample size. Cytokine responsiveness was evaluated in a smaller scale in the 300BCG cohort. Data were available only from PBMCs stimulated with *M. tuberculosis* and *S. aureus* for 24 h (IL-6, IL-1β and TNF-α) and 7 days (IFN-γ). We observed that 11-deoxycortisol negatively correlated to *S. aureus*-induced IL-6 production. However, the cytokine-related findings derived from the 500FG cohort could not be fully validated in the 300BCG cohort, due to the lack of data.

Taken together, these results further support for the association of 11-deoxycortisol in physiological conditions that is mainly manifested in the T cells.

### Effects of steroid precursors on T-cells *in vitro* is dose-related

**Fig. 5:**
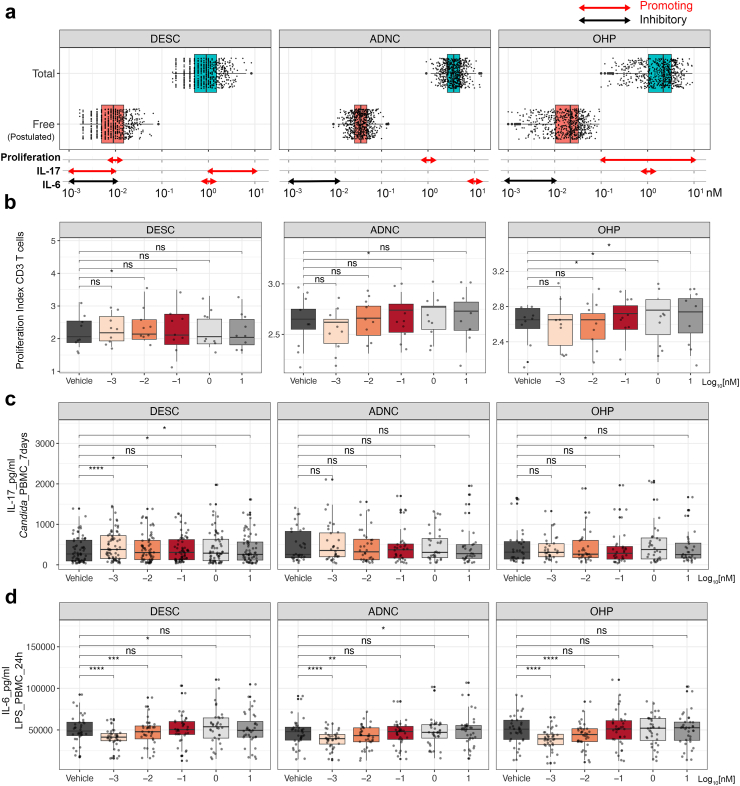
Dose response test of the three steroid precursors in functional experiments. (a) Overview of the working concentrations of 11-deoxycortisol, androstenedione and 17-OHP in different functional experiments. Pooled data of the total concentrations of steroid precursors measured in 500FG and 300BCG cohort are shown in the blue box (n = 585, female individuals taking oral contraceptives were excluded), and the postulated concentrations of the free from of steroid precursors (calculated as 1% of the total steroids) are shown in the red box. Red arrow indicates the working concentrations with promoting effects, and black arrows indicate the working concentrations with inhibitory effects. (b) Dose response test with T cell proliferation assay (n = 9). The proliferation index was calculated by the proliferation program in FlowJo using the compensated PB450-CellTraceViolet signal. (c) Dose response test with *C. albicans*-induced IL-17 production (11-deoxycortisol n = 67, androstenedione n = 32, 17-OHP n = 32). (d) Dose response test with LPS-induced IL-6 production (n = 36). In each box plot, the in-box line defines the median value, hinges depict the 25th and 75th percentiles and whiskers extend to ±1.5 interquartile ranges. Each dot represents one healthy volunteer. p-values were determined by Wilcoxon matched-pairs signed rank test comparing each dose with vehicle control, ∗p < 0.05, ∗∗p < 0.01, ∗∗∗p < 0.001, ∗∗∗∗p < 0.0001.

Previous studies have reported the broad immunosuppressive and anti-inflammatory effects of cortisol and sex hormones,^24^, ^25^, ^26^ however, few studies exist investigating the immunological relevance of their precursors. Although we observed the strongest positive association between 11-deoxycortisol and T cell immune traits, additional correlations with androstenedione and 17-OHP should not be overlooked. To further investigate the causal relationship and possible molecular mechanisms, we performed functional experiments with all three steroidogenesis precursors described above. The concentrations of the steroid precursors we obtained in both cohorts were consistent with the reported reference intervals.^27^ It should be noted that only 1–2% of the steroids in circulation are free and biologically active,^28^ thus we designed a wide concentration range covering both the total and (postulated) free concentrations of steroids in physiological conditions, ranging from 10^−3^ nM–10 nM. The association between hormone precursors and the cell counts and cytokine production capacity was tested *in vitro* using immune cells isolated from healthy volunteers. Fig. 5a provides an overview of the dose response test of 11-deoxycortisol, androstenedione, and 17-OHP, mapping to their total and free form concentrations as determined in the 500FG and 300BCG cohorts. The detailed data analyses are provided in Supplementary Fig. S4.

The stability of the T cell pool is primarily maintained via peripheral mechanisms. Homeostatic proliferation, instead of thymic output, contributes to the majority of naïve and memory T cell repertoire in adults.^29^^,^^30^ T cell proliferation requires the co-stimulation of TCR-CD3/CD28 complex by antigen-presenting cells, hence we used CD3/CD28 dynabeads to mirror this stimulation. We found that all three steroids were able to promote T cell proliferation, but the effective concentrations differed (Fig. 5b). The presence of 11-deoxycortisol *in vitro* at the concentration of 10^−2^ nM significantly augmented CD3^+^ T cell proliferation when compared with vehicle control, which was close to the postulated concentration of its free form in circulation. Androstenedione and 17-OHP also increased CD3^+^ T cell proliferation at 1 nM and 10^−1^–10^1^ nM, respectively, both of which were above the physiologically achievable concentrations.

Cytokine responses were tested with 24 h LPS and 7-day *C. albicans* stimulation. We found that 11-deoxycortisol significantly upregulated the *C. albicans*-induced IL-17 production at a wide concentration range, covering both the lower end (10^−3^–10^−2^ nM) and upper end (10^0^–10^1^ nM). Similar effect was observed in the 17-OHP-treated group, but only at the higher concentration (1 nM), and no effects of androstenedione on IL-17 production were observed at any dose (Fig. 5c). Additionally, all three steroid precursors reduced LPS-induced IL-6 production at 10^−3^–10^−2^ nM (Fig. 5d).

In summary, the working concentration of 11-deoxycortisol in functional assays was more relevant to physiological concentrations as compared to androstenedione and 17-OHP, which could possibly explain the more pronounced association of 11-deoxycortisol to immune traits in healthy volunteers.

### 11-deoxycortisol positively regulates T cell proliferation and activation *in vitro*

**Fig. 6:**
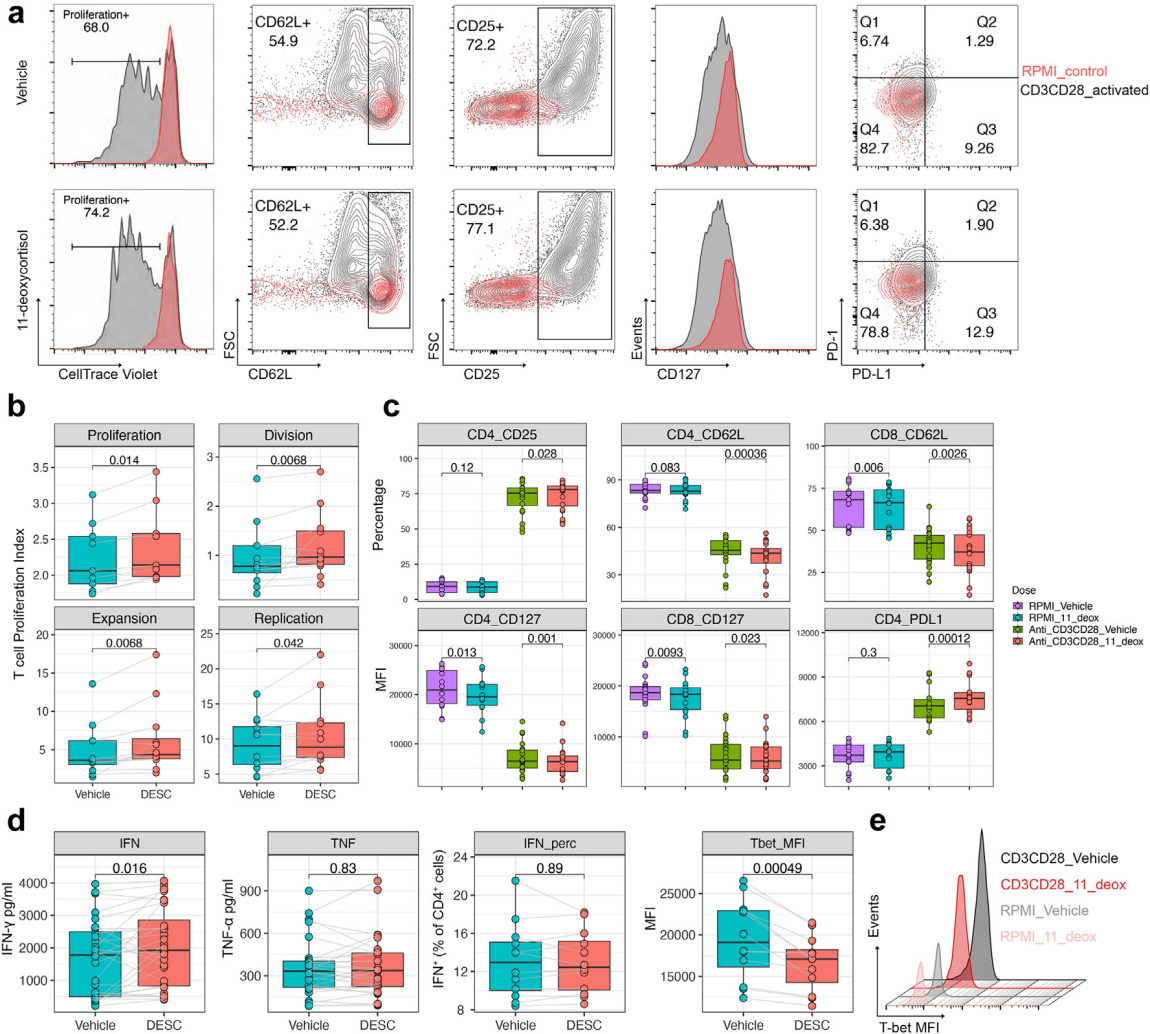
11-deoxycortisol positively regulates T cell proliferation and activation *in vitro*. (a) Representative overlays of RPMI control versus CD3/CD28 activated T cells in the presence (lower panel) or absence (upper panel) of 11-deoxycortisol. PBMCs were cultured with CD3/CD28 dynabeads for 4 days. 11-deoxycortisol at the concentration of 10^−2^ nM was tested in all the conditions. The expression of CD25 and CD62 was compared using the frequency of positive sub-populations, and the expression of CD127, PD-1 and PD-L1 was compared using the median fluorescence intensity (MFI) on gated CD3^+^CD4^+^ or CD3^+^CD8^+^ single cells. (b) Statistical analysis of the T cell proliferating parameters in the presence or absence of 11-deoxycortisol (n = 9), including expansion index (fold-expansion of the overall culture), division index (average number of cell divisions that a cell in the original population has undergone), proliferation index ((total number of divisions)/(number of divided cells)) and replication index (fold-expansion of only the responding cells). (c) Statistical analysis of the expression of the T cell activation markers in the presence or absence of 11-deoxycortisol (n = 14). RPMI_Vehicle versus RPMI_11_deox was compared, Anti_CD3CD28_Vehicle versus Anti_CD3CD28_11_deox was compared. (d) Statistical analysis of the cytokine production capacity in CD3/CD28-activated T cells in the presence or absence of 11-deoxycortisol. IFN-γ and TNF-α in the culture supernatants of CD3/CD28-activated T cells were measured by ELISA (n = 28). For the IFN-γ-producing cells, the frequency within the CD4^+^ T cells and MFI of T-bet was determined by flowcytometry after intracellular staining (n = 12). (e) Representative histogram overlay of T-bet fluorescence intensity on gated IFN-γ-producing CD4^+^ T cells from one donor (out of 12 donors tested). In each box plot, the in-box line defines the median value, hinges depict the 25th and 75th percentiles and whiskers extend to ±1.5 interquartile ranges. Each dot represents one healthy volunteer. p-values were determined by Wilcoxon matched-pairs signed rank test are shown in the figures.

We subsequently evaluated the effects of 11-deoxycortisol on T cell activation, with a special focus on the concentration of 10^−2^ nM. Specifically, the CD3/CD28-responding T cells underwent approximately 1–6 rounds of division within 4 days in both control and tested groups, and the dividing T cell fraction significantly increased after exposure to 11-deoxycortisol (Fig. 6a). The indices of cell proliferation are shown in Fig. 6b. Proliferation index, replication index and expansion index were consistently higher in the 11-deoxycortisol group. Naïve T cells downregulate the expression of CD62L (L-selectin) and CD127 (IL-7 receptor) in the initial expansion phase of activation, thus redirecting from lymph nodes to sites of infection.^31^^,^^32^ Flow cytometry showed that 11-deoxycortisol lowered the expression of CD127 and CD62L on both CD4^+^ and CD8^+^ T cells. However, it should be noted that the effects on CD62L and CD127 expression were not unique to 11-deoxycortisol, similar effects were also observed in cells treated by androstenedione and 17-OHP (Supplementary Fig. S4). Ligation of the CD3/CD28 co-receptors could significantly induce the expression of CD25 (IL-2 receptor) and PD-L1 in T cells.^33^^,^^34^ In line with this, we observed increased CD25 and PD-L1 expression (Fig. 6c, Supplementary Fig. S5), which was highly restricted to 11-deoxycortisol at the concentration of 10^−2^ nM. In addition, we quantified the cytokines in the supernatants of CD3/CD28 stimulated PBMCs treated with 11-deoxycortisol. Data analysis showed that 11-deoxycortisol treatment led to a statistically significant increase in the production of IFN-γ (Fig. 6d). However, no difference in the frequency of IFN-γ-producing CD4^+^ cells was observed, and the expression of T-bet, the transcription factor regulating IFN-γ production in Th1 cells,^35^ was reduced after 11-deoxycortisol treatment (Fig. 6d and e). Similarly, we observed no significant difference in the expression of CXCR3 (Supplementary Fig. S5), the chemokine receptor directly activated by T-bet.^36^

Collectively, these results suggest that 11-deoxycortisol facilitates T cell activation *in vitro* upon CD3/CD28 co-stimulation.

### 11-deoxycortisol promotes *in vitro* induction of CD4^+^Th17 cells by *C. albicans*

**Fig. 7:**
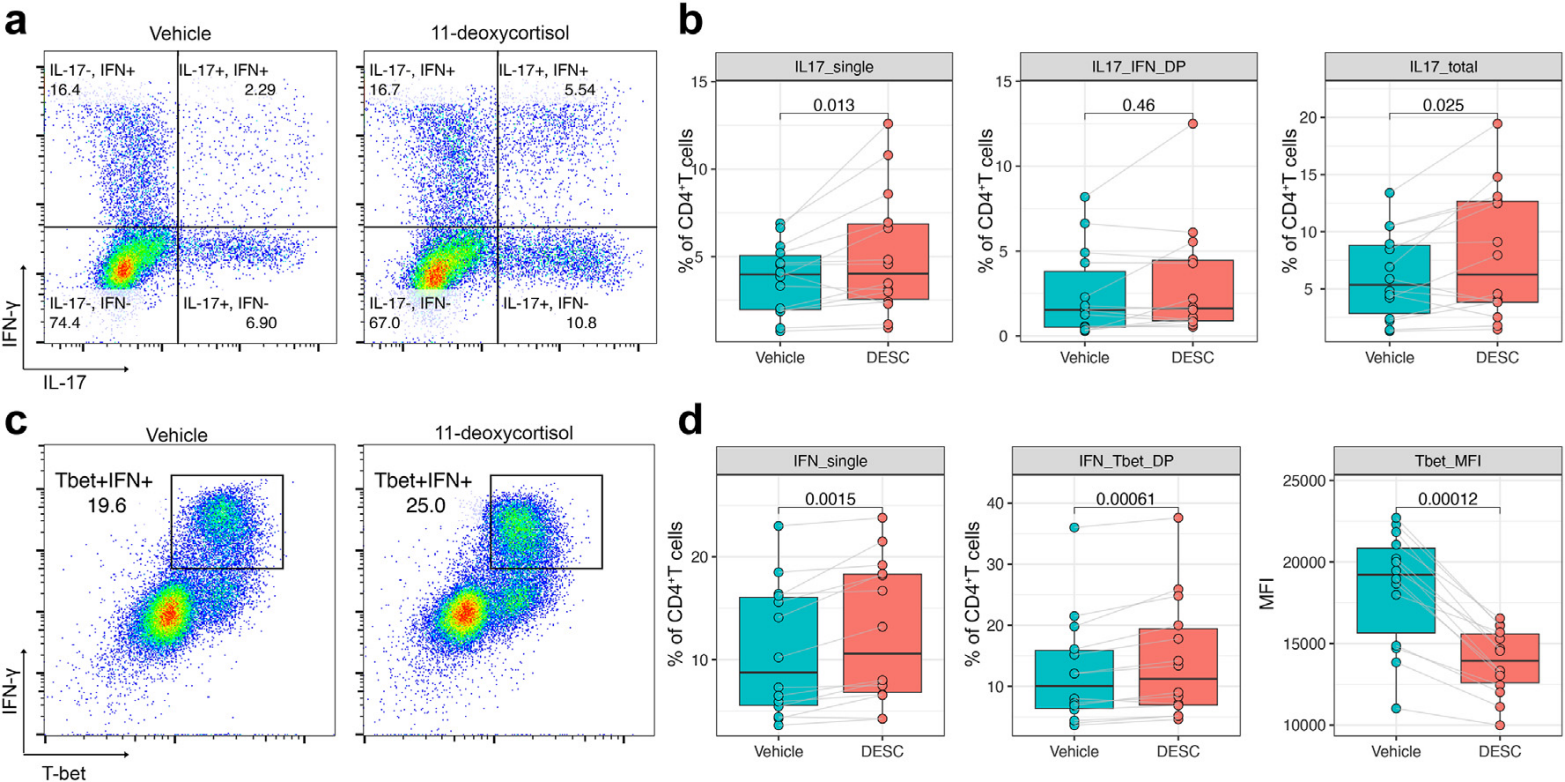
11-deoxycortisol promotes *in vitro* induction of CD4^+^Th17 cells by *C. albicans*. (a) Representative FACS plots of IL-17 and IFN-γ production on gated CD3^+^CD4^+^ T cells from one donor (out of 14 donors tested). (b) Statistical analysis of the proportions of the IL-17_single (lower right quadrant), IL-17_IFN-γ_double positive (upper right quadrant), and IL-17_total (lower right and upper right quadrant combined) subsets on gated CD3^+^CD4^+^ T cells (n = 14). (c) Representative FACS plots of IFN-γ and T-bet co-staining on gated CD3^+^CD4^+^ T cells from one donor (out of 14 donors tested). (d) Statistical analysis of the proportions of the IFN-γ_single (upper left quadrant in A), IFN-γ_T-bet_double positive (the gated cell population in C) on gated CD3^+^CD4^+^ T cells, and MFI of T-bet in IFN-γ-producing CD4^+^ T cells (n = 14). PBMCs were cultured with heat-inactivated *C. albicans* for 7 days and stimulated with PMA, ionomycin plus Golgi plug for 4 h prior to FACS staining. 11-deoxycortisol at the concentration of 10^−2^ nM was tested in all the conditions. In each box plot, the in-box line defines the median value, hinges depict the 25th and 75th percentiles and whiskers extend to ±1.5 interquartile ranges. Each dot represents one healthy volunteer. p-values as determined by Wilcoxon matched-pairs signed rank test are shown in the figures. IL17_IFN_DP, IL-17_IFN-γ_double positive; IFN_Tbet_DP, IFN-γ_T-bet_double positive; PMA, phorbol myristate acetate.

IL-17 production is the hallmark of CD4^+^ T helper 17 cells (Th17), which play a crucial role in mucosal immunity against fungal infections and can be induced by *C. albicans in vitro*.^37^ To investigate whether 11-deoxycortisol promotes the induction of Th17 cells, we cultured PBMCs with *C. albicans* for 7 days and performed intracellular staining of IL-17, IFN-γ and RAR-related orphan receptor gamma t (RORγt), a nuclear receptor essential for the generation of Th17 cells,^38^ on gated viable CD3^+^CD4^+^ fraction. No significant T cell proliferation was observed in this setting. Flowcytometry revealed that a small fraction of CD4^+^ T helper cells acquired the capacity to produce IL-17 and IFN-γ, and showed overexpression of RORγt, thus confirming the induction of Th17 cells (Supplementary Fig. S6). We observed that 11-deoxycortisol significantly upregulated the percentage of IL-17^+^ subsets within the CD4^+^ cell population, with a slight increase in the IFN-γ-producing CD4^+^ T cells (Fig. 7a and b). Co-staining of T-bet and IFN-γ showed increased percentage of T-bet^+^ IFN- γ^+^ cells, but the intensity of the T-bet fluorescence was significantly reduced (Fig. 7c and d), which was highly consistent with the lowered T-bet expression we observed in the T cell activation experiments.

To investigate whether 11-deoxycortisol works on its own or in collective action with multiple steroids, similar experiments were performed with steroid-stripped HPS. We found that the induction of Th17 cells by *C. albicans* was substantially inhibited when cultured in steroid-stripped HPS (Supplementary Fig. S7). In this setting, IL-17 production was significantly inhibited while IFN-γ concentration was elevated. Meanwhile, the IL-22 level remained unaffected. These results suggest that T cell polarization was skewed towards a predominant Th1 response upon *C. albicans* challenge when the culture medium was depleted of steroids. The addition of 11-deoxycortisol was not able to restore the Th17 cell generation, with no significant differences among various concentrations of 11-deoxycortisol being observed.

Taken together, these data suggest the capability of 11-deoxycortisol to promote Th17 response induced by *C. albicans*, whereas the optimal effect requires the presence of other endogenous steroids.

## Discussion

Besides their role in cortisol synthesis, the intrinsic biological activities of steroidogenesis intermediates have been poorly understood. In steroidogenic disorders like Cushing's disease (hypercortisolism) or Addison's disease (hypocortisolism), the overproduction or underproduction of 11-deoxycortisol is generally accompanied by drastic changes in cortisol concentrations, which imposes challenges to delineate the 11-deoxycortisol-specific effects.^9^ In this study, by correlating steroids profile with immunological parameters in two large cohorts of healthy volunteers, we described a strong association of 11-deoxycortisol, but not cortisol, with adaptive immune traits (mainly lymphoid cell numbers and function), which was robust and reproducible. Importantly, integration of *in vivo* findings with *in vitro* validation experiments supports the immunoregulating property of 11-deoxycortisol by promoting T-cell replication and Th17 polarization.

We identified that the positive associations of 11-deoxycortisol were primarily manifested in lymphoid “progenitor” like cellular subsets (naïve and memory cells). Naïve and memory T-cells have a longer life-span compared to effector cells, which are terminally differentiated and quickly undergo apoptosis after infection.^30^^,^^39^ When lymphocyte counts drop below a certain threshold, naïve T-cells replicate upon tonic TCR stimulation by self-antigens, also known as homeostatic T-cell proliferation, of which the underlying mechanism remains largely uninvestigated.^40^, ^41^, ^42^ In our study, we found that 11-deoxycortisol promotes T-cell proliferation *in vitro*, supporting a causal effect of endogenous steroids on immune maintenance in steady-state conditions. T-cell compartment shrinkage is one of the hallmarks of the declined immune competence as humans age (immunosenescence).^43^ Our findings provide preclinical evidence suggesting that 11-deoxycortisol could be one of the factors involved in maintaining adaptive immune function during aging.

The generation of memory T-cells provides efficacious antigen-specific protection upon reinfection.^30^ Th17 cells, a specific subset of tissue resident memory T-cells,^44^ fine-tune the balance between mucosal protective and pathogenic immunity. *C. albicans* is the major inducer of Th17 cells *in vivo* due to its early colonization in the microbiota since birth.^45^ We confirmed that *C. albicans* elicited a strong Th17 response in PBMCs, as previously described,^46^ while IL-17 defects are mainly accompanied by fungal mucosal infections.^47^ Our *in vitro* experiments validated the assumption that 11-deoxycortisol promotes IL-17 production, even though individual responses were variable. During Th17 induction, monocytes within the PBMCs fraction function as antigen presenting cells and generate polarizing cytokines (IL-1β, IL-23 and IL-6) upon encounter with *C. albicans*, modeling the differentiation of Th17 cells from naïve T-cells in intestine.^37^^,^^46^ We cannot exclude that 11-deoxycortisol may facilitate this process *in vivo*. To identify the precise cellular target of 11-deoxycortisol, future studies could consider using sorted monocytes and naïve CD4^+^ T-cells to assess the effect of the hormone on the activation of these immune cell populations, or purified *Candida*-specific memory Th17 cells to assess whether 11-deoxycortisol promotes the expansion of memory cells.

Interestingly, we also found that the optimal effect of 11-deoxycortisol requires the presence of other endogenous steroids. Supporting our findings, studies have shown that sex hormones (e.g., estrogen and progesterone) promote IL-17A production in Th17 cells, linking to the sex bias of Th17-related immune pathologies such as autoimmune disease and *Candida* infection.^48^^,^^49^ Understanding the underlying mechanism of how 11-deoxycortisol works together with sex hormones in regulating the polarization of pathogenic Th17 cells can help towards developing sex-specific therapies.

An outstanding question is how 11-deoxycortisol exerts its effects at a molecular level. We observed lower T-bet expression in both functional experiments, suggesting inhibited Th1 differentiation. Future study investigating the influence of the steroid precursors in the balance between Th1 and Th17 differentiation would be of great interest. RORγt, a member of the nuclear hormone receptors (NHRs) family, directs the differentiation program of Th17 cells.^38^ More importantly, RORγt is essential for thymocyte development and lymphoid organogenesis.^50^ Thus, RORγt might be a highly relevant 11-deoxycortisol target. It is known that small lipophilic molecules like hormones and steroids are natural ligands for NHRs, which function as crucial transcriptional regulators of multiple biological activities within immune cells.^51^, ^52^, ^53^ Recent studies have identified several endogenous ligands for RORγt as immune modulators, including canonical cholesterol biosynthetic intermediates (CBIs) and cholesterol derivatives such as 4ACD8 and oxysterols,^54^^,^^55^ highlighting the role of endogenous biosynthesis intermediates or metabolites in shaping the immune landscape. Unlike the steroidogenesis pathway that highly restricts within the adrenal cortex, cholesterol biosynthesis is universal in mammalian cells. Given the abundance of intracellular CBIs and their high affinity to RORγt,^54^ it is reasonable to assume that the majority of available RORγt in lymphocytes is occupied by endogenous ligands, which could possibly explain the moderate or even subtle effect of exogenous 11-deoxycortisol in our validation experiments. However, the agonist activity of 11-deoxycortisol for RORγt or other NHRs warrants further investigation.

Interestingly, another steroidogenesis intermediate, pregnenolone, has recently been shown to have immunomodulatory properties in mouse models.^56^ Pregnenolone, immediately catalyzed from cholesterol by *Cyp11a1*, is the common precursor for all the steroid hormones. Traditionally thought to be restricted in the adrenal cortex cells, however, researchers have confirmed the expression of *Cyp11a1* and detected substantial concentrations of pregnenolone in immune cells.^57^ More interestingly, inhibition of the *de novo* steroidogenesis in T cells promoted anti-tumor response in the tumor microenvironment.^58^ Inspired by this study, we originally hypothesized that *de novo* steroidogenesis of 11-deoxycortisol from immune cells could be the possible explanation of the positive correlation observed in both healthy cohorts. However, transcriptomic analysis of the PBMCs in 500FG cohort was not able to demonstrate expression of *Cyp21a2* in immune cells, thus excluding the hypothesis.

No correlation of 11-deoxycortisol with the myeloid cell numbers and functionality were observed in either of the two cohorts. Previous studies have shown that the behaviors of myeloid cells are more predominantly regulated via intrinsic cell-autonomous mechanisms, while the adaptive immune traits are more dependent on extrinsic cues like the endocrine signals.^59^ Somewhat counterintuitive considering the concept that cortisol is a profound immunomodulator,^5^^,^^60^ no strong correlation of cortisol to immune traits was observed. A possible explanation for the discrepancies in the associations between the immune traits and steroids, is the highly dynamic nature of the concentrations of these hormones in blood. Cortisol is released into circulation in a circadian and pulsatile manner, which peaks at the onset of active phase and decreases during the resting phase.^61^ Therefore, in our study, random sampling could not fully reflect the HPA axis activity. On the other hand, the ingress and egress of immune cells from lymphoid organs and bone marrow are under the tight control of circadian rhythm. Lymphocytes home to lymphoid nodes at active phase and migrate into circulation at resting phase, indicating that the lymphoid cellularity and cortisol level in the blood display opposite rhythms,^62^ hence the involvement of circadian rhythm could possibly confound the results of correlation analysis. Also, it has been shown that different steroid hormones display distinct circadian patterns that may not coincide with that of cortisol.^63^ Whether 11-deoxycortisol also displays the same circadian rhythm as cortisol is not yet well described.

The major strength of the study lies in the size of the cohorts included, and the comprehensive nature of the analysis, further supported by independent validation and mechanistic studies. To the best of our knowledge, this is the largest study comprehensively assessing the associations between physiological levels of steroid hormones and different innate and adaptive immune cells numbers and function. However, our study has also certain limitations, and further investigations are warranted in several aspects. Firstly, we cannot exclude that the differences in circadian rhythm of the different hormones may have obscured some associations with the immunological characteristics. To minimize the effect of the circadian rhythm, all blood samples were taken in the morning. Secondly, the validation cohort was relatively small and excluding the women taking OCP had further reduced the sample size—this may also have obscured some of potentially significant associations. Nonetheless, the consistency of the results complemented with the mechanistic validation studies argue strongly in favor of the robustness of the associations found. Thirdly, since only individuals of Western European ancestry and relatively few elderly individuals were included in the 500FG and 300BCG cohorts, future studies recruiting people of other ancestries and a wider range of ages are needed. Finally, the current study was conducted in a healthy population, thus our findings must be interpreted cautiously. In pathological conditions, there is also a dissociation of steroids with their binding globulins, peripheral distribution and metabolism, and a lack of circadian rhythmicity, compared to the physiological situations.^64^ How 11-deoxycortisol and other steroid hormones concentrations change overtime when the immune system is challenged by pathogens or malignancies remains largely unknown, hence it might be revealing to perform longitudinal monitoring in immunologically-related diseases and to explore the clinical implications.

In conclusion, the present comprehensive assessment of the relationship between steroid hormones and immune functions in a healthy population indicates a previously undescribed association of 11-deoxycortisol with stimulatory effects on lymphoid cells, an effect which could be leveraged in clinical conditions characterized by lymphoid cells defects. On the other hand, it will be of interest for future research to investigate whether such mechanisms are involved and can be modulated in pathological conditions, for example, to treat T-cell (or Th17 specific)-driven diseases including autoimmune diseases and organ transplant rejections.

## Contributors

Conceptualisation: RTNM, CJX, MGN. Data curation: VACMK. Formal analysis: CJX, XJ. Funding acquisition: MGN. Investigation: CP, MJ, PvH, AEvH, SJCFMM, VPM, HL, HD, HJPMK, IJ, BvC. Project administration and supervision: YL, LABJ, RTNM, CJX, MGN. Visualisation: CP, XJ. Writing—original draft: CP. RTNM, CJX, and MGN have accessed and verified the underlying data. All authors read and approved the final version of the manuscript. RTNM and CJX were responsible for the decision to submit the manuscript.

## Data sharing statement

Most data from the HFGP are freely available (Data—Human Functional Genomics Project). Other data collected in this study are available from the corresponding author upon reasonable request.

## Declaration of interests

The authors declare that there is no conflict of interest.

## Appendix A Supplementary data

Supplementary Figures and Tables

500FG study protocol

300BCG study protocol
