# Supplementary material for: 11-deoxycortisol positively correlates with T cell immune traits in physiological conditions
Source: eBioMedicine. 2023 Dec 21;99:104935. doi: 10.1016/j.ebiom.2023.104935 (PMC10776925; doi:10.1016/j.ebiom.2023.104935)
Supplement: Supplementary Figures and Tables [file mmc1.docx]

**Table of contents**

**Methodological Appendix**

**Figure S1, Correlations among the steroids in derivation and validation cohorts.**

**Figure S2, Replication in 300BCG, FDR adjustment not applied.**

**Figure S3, Gating strategy of T cell proliferation assay.**

**Figure S4, Dose response tests of androstenedione and 17 hydroxy progesterone**

**Figure S5, The effects of 11-deoxycortisol on T cell activation markers are does-related**

**Figure S6, Verification of the induction of Th17 cells**

**Figure S7, Immunological effects of 11-deoxycoritsol in steroid-stripped serum.**

**Table S1, Immune cell subsets in 500FG and 300BCG cohorts.**

**Table S2, Cytokine production in *ex vivo* stimulation experiments in 500FG cohort.**

**Table S3, Comparative analysis of the steroid concentrations in the 500FG and 300BCG cohort.**

**Table S4, Antibody information**

**Methodological Appendix**

**Immune phenotyping procedures**

Fresh peripheral blood cells were counted using a cell counter. The absolute number of white blood cells (WBC) per ml of blood determined by the cell counter was used to calculate the absolute numbers of CD45+WBC cell subsets as measured by flow cytometry. Both erythrocyte-lysed whole blood samples (panel 1-3) and density gradient isolated PBMC (panel 4) were analyzed by flow cytometry. Each sample was analyzed by four 10-color antibody panels: 1. general, 2. T cell, 3. B cell and 4. intracellular T cell/Treg. For each panel, the single cells within the leukocyte (CD45+) population were first gated and thereafter the major myeloid or lymphoid lineages identified. Single cells were identified by plotting the FS Time of Flight (FS TOF) against FS. In panel 1, granulocytes and lymphocytes were discriminated by forward scatter and side scatter, while monocytes were characterized by CD14 expression. Within the lymphocytes, T cells (CD3+, CD56-), NK cells (CD3-, CD56+), CD3+ CD56+ T cells and B cells (CD19+, HLA-DR+) were characterized. Subpopulations within T cells, NK cells and monocytes were analyzed by CD4 and CD8, CD56 and CD16, and CD14 and CD16 expression, respectively. In panel 2, CD4+ regulatory T cells (Treg, CD4+, CD25+ CD127 low) and CD45RA/CD27 and CD45RO/CD27 maturation stages of CD4 and CD8 T cells were identified. Panel 3 aimed to define CD19+ B cell maturation stages by the expression of IgM/IgD and/or CD24/CD38 expression. B cell subsets were additionally identified by differential CD19/CD20 and IgD/CD5 expression. Sub-phenotypic information was provided in the table due to the multiple gating strategies used; In panel 4, the major T cell populations (CD4, CD8 and Treg) were identified and subsequently analyzed for proliferation status by intracellular Ki67 expression. Treg (CD4+, CD25+ CD127low FOXP3+) were analyzed for expression of CD45RA and HLA-DR. Absolute cell counts in panel 4 (PBMC obtained after density gradient isolation) were calculated as described above taking into account the whole blood cell counts minus the granulocyte number as determined in panel 1.


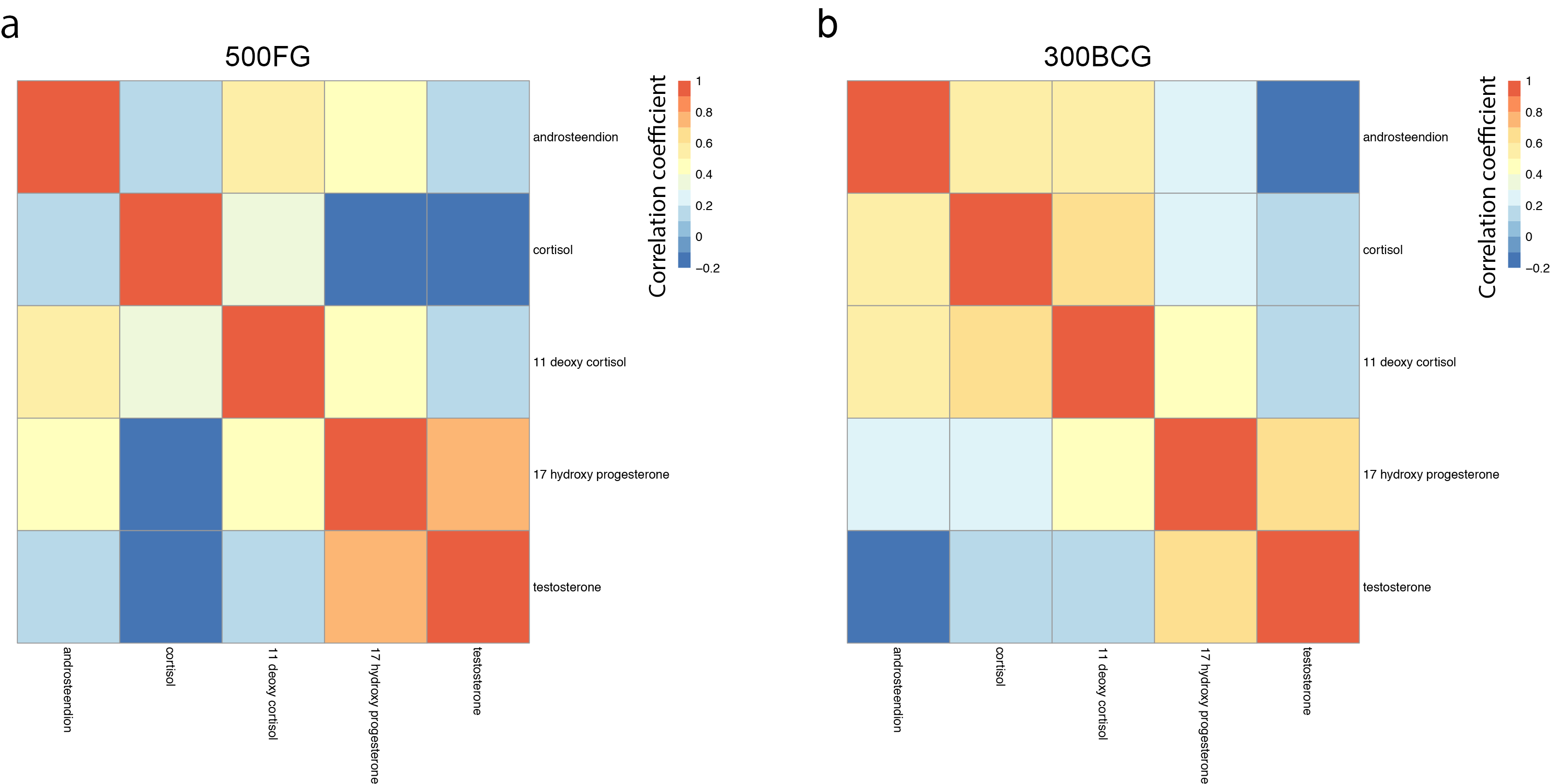


**Figure S1, Correlations among the steroids in derivation and validation cohorts**

(a) Correlation heatmap of steroids from the 500FG cohort (n=484). (b) Correlation heatmap of steroids from the 300BCG cohort (n=232). Colors indicate correlation coefficients.


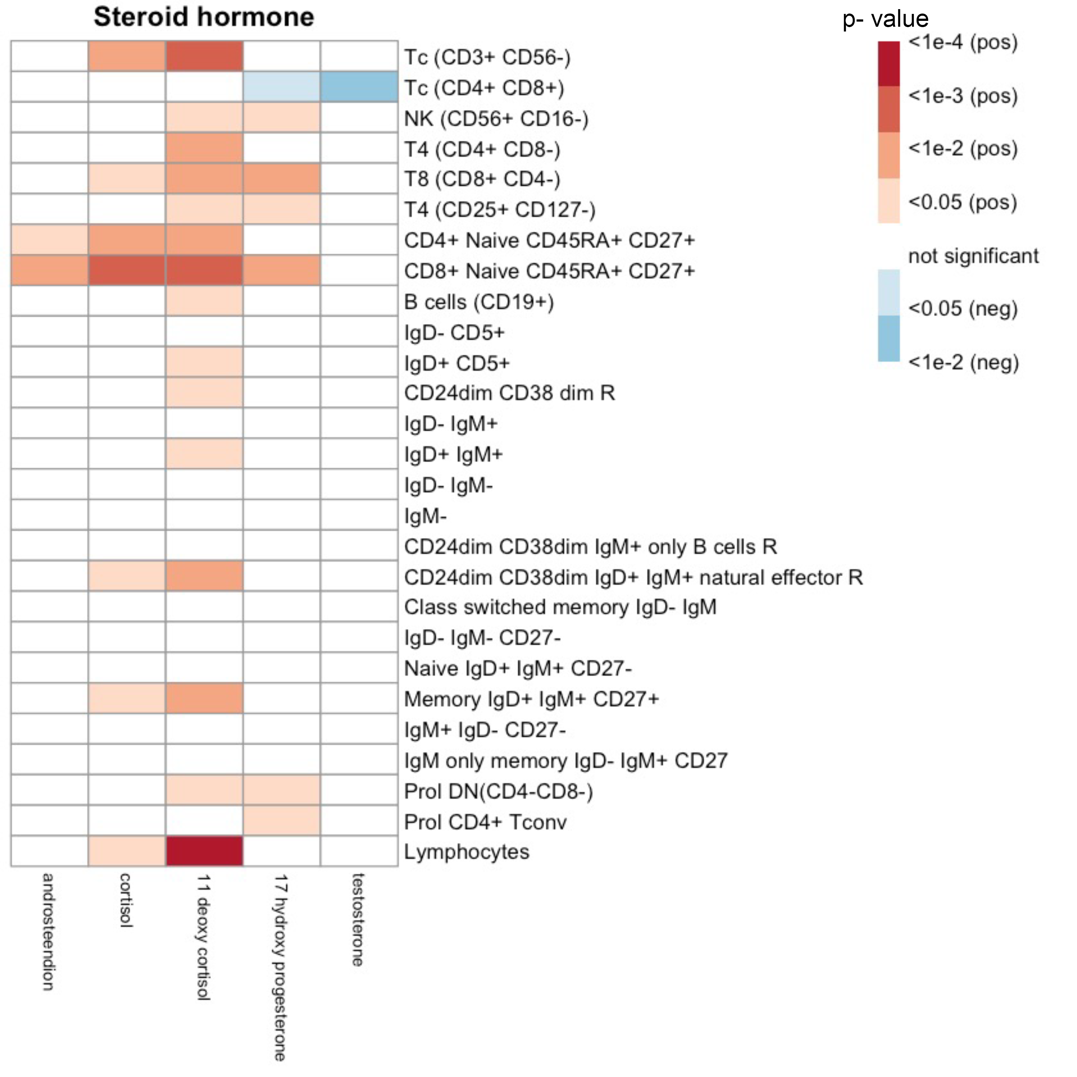


**Figure S2, Replication in 300BCG, FDR adjustment not applied**

Correlation of immune traits to testosterone, cortisol, androstenedione, 11-deoxycortisol, 17 hydroxy progesterone in the 300BCG cohort, after correcting age, gender (n=232). FDR adjustment was not applied, correlations do not reach statistical significance are shown as blank.


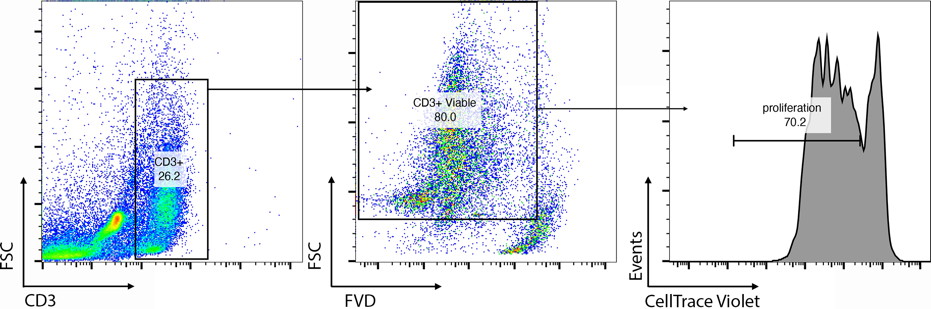


**Figure S3, Gating strategy of T cell proliferation assay.**

Viable CD3^+^ T cells were gated based on APC-A700-CD3 and fixable viability dye (FVD), after which T cell proliferation was analyzed on the PB450-CellTrace Violet signal.


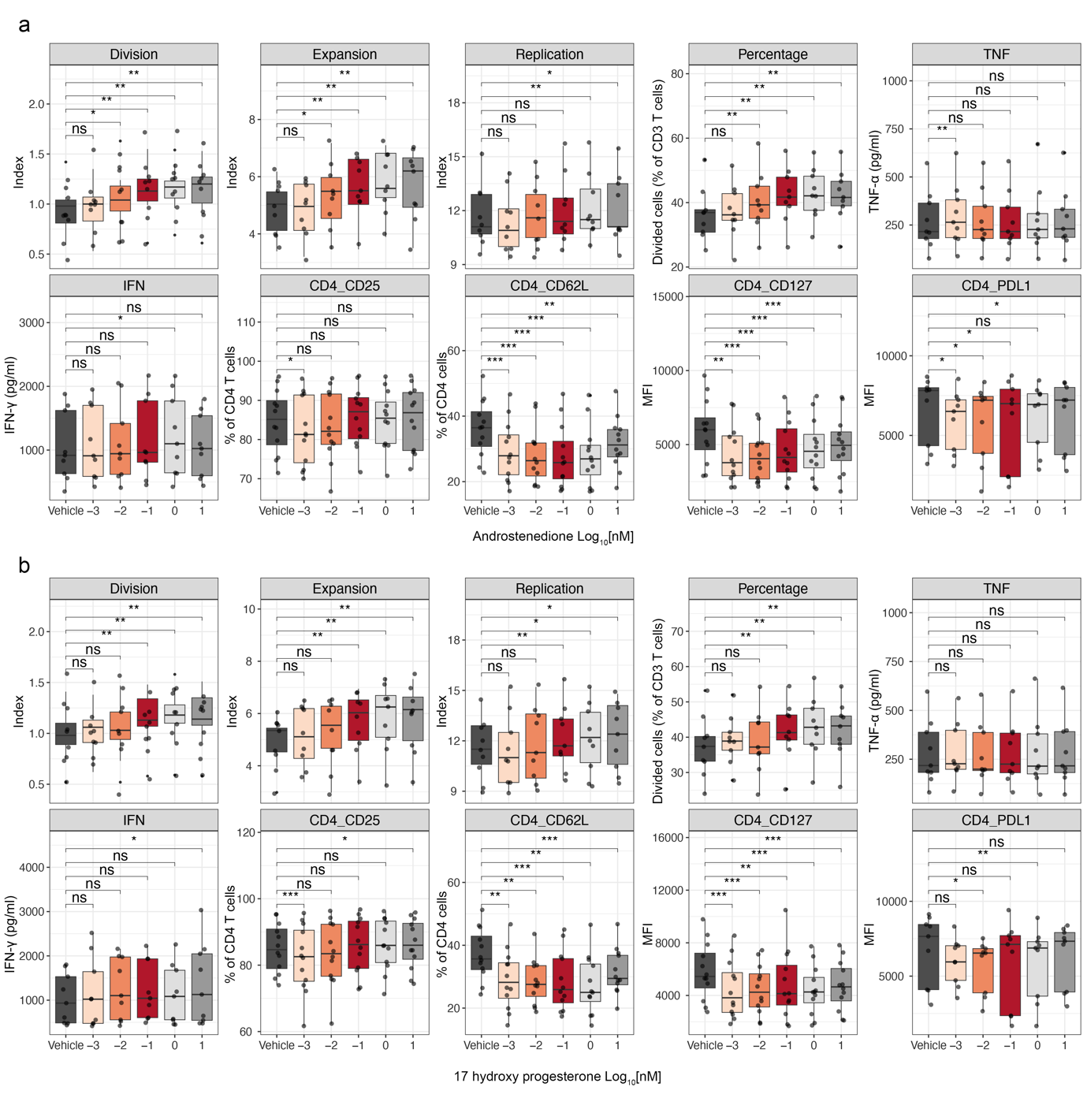


**Figure S4, Dose response tests of androstenedione and 17 hydroxy progesterone**

(a) Dose response test of androstenedione in CD3CD28-activated settings. (b) Dose response test of 17-hydroxy progesterone in CD3CD28-activated settings. PBMCs labeled with CellTrace Violet were stimulated with anti-CD3CD28 dynabeads for 4 days in the presence of androstenedione or 17 hydroxy progesterone at indicated concentrations. Division index, expansion index, replication index and the percentage of the divided cells are calculated by the proliferation program in Flowjo using the compensated PB450-CellTrace Violet fluorescence (n=9). The concentrations of TNF-α and IFN-γ in the cell culture supernatants were quantified by ELISA (n=9). The frequencies of CD25-positive and CD62L positive cells, and the MFI of CD127 and PD-L1 within the CD4+ T cell population were determined by flowcytometry (n=12). Each dose was compared with vehicle control, p-values were determined by Wilcoxon signed rank test, * *p* < 0.05, ** *p* < 0.01, *** *p* < 0. 001. ns, not significant.


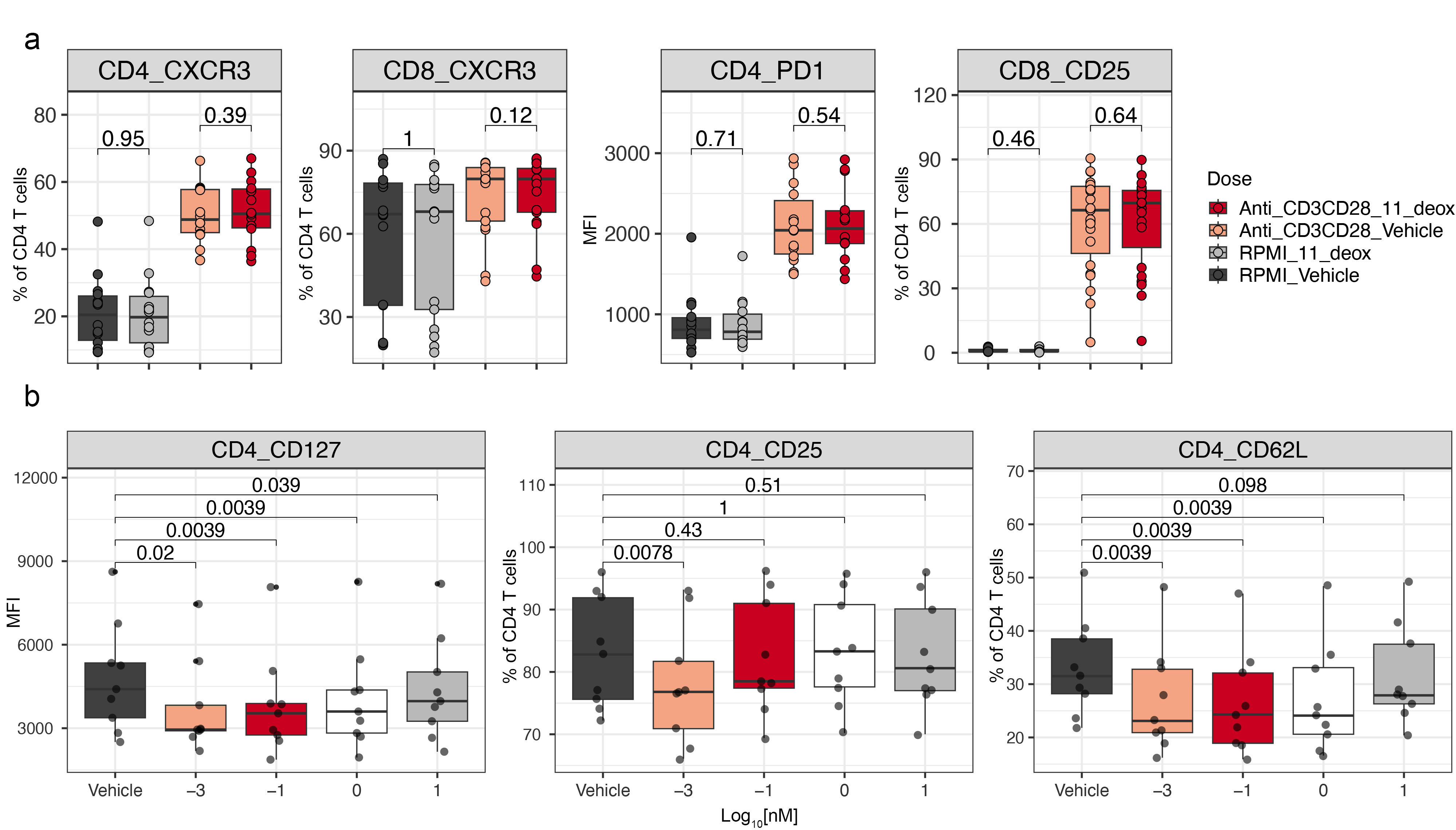


**Figure S5, The effects of 11-deoxycortisol on T cell activation markers are does-related**

(a) The frequencies of CXCR3^+^CD4^+^, CXCR3^+^CD8^+^ and CD25^+^CD8^+^ cell populations, and the expression of PD-1 on CD4^+^ T cells were not affected by 11-depxycortisol (n=14). RPMI_vehicle versus RPMI_11_deox was compared, Anti_CD3CD28_vehicel versus Anti_CD3CD28_11_deox was compared. (b) 11-deoxycortisol downregulates the expression of CD62L and CD127 on CD4^+^ T cells at a broad dose range (n=9). Each dose was compared with vehicle control. p-values were determined by Wilcoxon signed rank test, * *p* < 0.05, ** *p* < 0.01, *** *p* < 0. 001. ns, not significant.


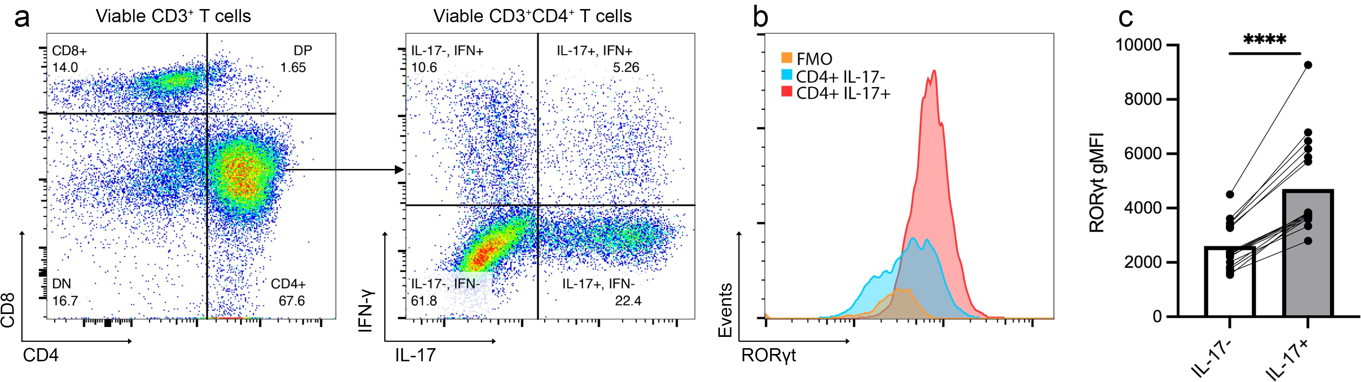


**Figure S6, Verification of the induction of Th17 cells.**

PBMCs were primed with heat-inactivated *C. albicans* for 7 days. Cells were stimulated with PMA and ionomycin in the presence of Golgi plug for 5 hours. (a) The gating of CD4^+^, CD8^+^, double positive (DP) and double negative (DN) of the CD3^+^ viable cells. (b) Intracellular IL-17 and IFN production by CD4^+^ T cells. (c) Histogram of RORγt expression, IL-17^+^ CD4^+^ and IL-17^-^ CD4^+^ cell populations were compared, FMO control was used for gating. (D) pooled data analysis of RORγt expression as determined by gMFI. IL-17^-^CD4^+^ cells versus IL-17^+^CD4^+^ cells were compared, n=17. **** *p* < 0.0001 by Wilcoxon test.


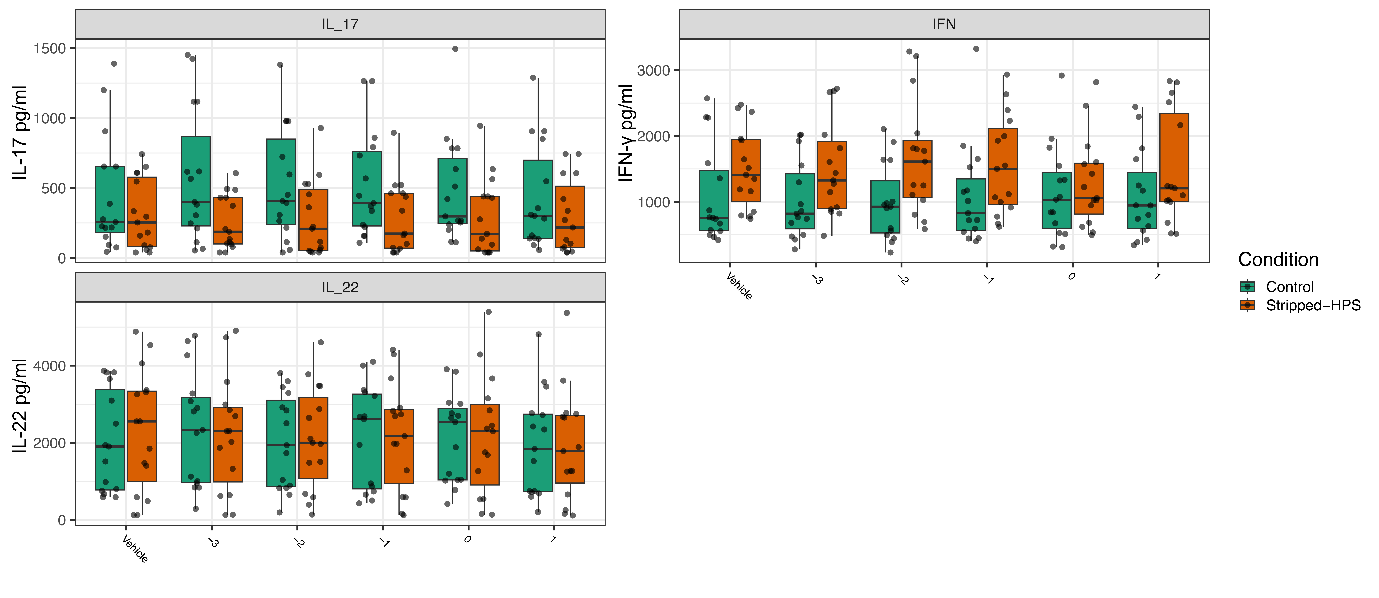


**Figure S7, Immunological effects of 11-deoxycoritsol in steroid-stripped serum.**

PBMCs were primed with *C. albicans* in the presence of 10% steroid-stripped HPS for 7 days, after which, IFN-gamm(a)IL-17 and IL-22 levels in the supernatant were determined by ELISA. Each dot represents one healthy volunteer (n=15). Steroid-stripped HPS was prepared by incubating HPS with dextran-treated-charcoal overnight at 4 degree, HPS treated in the same way without charcoal was used as control. A ten-fold serial dilution of 11-deoxycortisol was tested, methanol vehicle was used as control. Working concentrations are shown as –Log_10_[M].

**Table S1, Immune cell subsets in 500FG and 300BCG cohorts**

| WBC | Monocytes (CD14+) | T cell prol | | Prol DP (CD4+CD8+) |
| --- | --- | --- | --- | --- |
|  | Lymphocytes |  |  | Prol DN (CD4- CD8-) |
|  | Granulocytes |  |  | Prol CD8 |
| Monocytes | Non-classical monocytes (CD14++CD16+) |  |  | Prol CD4+ Treg |
|  | Intermediate monocytes (CD14+CD16+) |  |  | Prol CD4+ Tconv |
|  | Classical monocytes (CD14++ CD16-) | T Reg | | Treg HLA-DR+ |
| Lymphocytes | T cells (CD3+ CD56-) |  |  | Treg CD45RA+ |
|  | T cells (CD3+ CD56+) |  |  | Treg CD45RA- |
|  | NK cells (CD3- CD56+) | NK cells | | NK dim (CD56+ CD16+) |
|  | B cells (CD19+) |  |  | NK bright (CD56++ CD16-) |
| T cells | DP (CD4+ CD8+) |  |  | NK (CD56+ CD16-) |
|  | DN (CD4- CD8-) | B cells | -/+ CD20 | CD19+ CD20+ B cells |
|  | CD8+ T cells |  |  | CD19+ CD20- plasma blasts/cells |
|  | CD4+ T cells |  | IgD/CD5 | IgD- CD5++ |
|  | Treg CD25+ CD127low |  |  | IgD+ CD5++ |
|  | CD8+ Naive CD45RO- CD27+* |  |  | IgD- CD5+ |
|  | CD8+ naive CD45RA+ CD27+ |  |  | IgD+ CD5+ |
|  | CD8+ EM CD45RO+ CD27- |  | CD24/CD38 | CD24++ CD38++ Transitional B cells |
|  | CD8+ EM CD45RA+ CD27+ |  |  | CD24+ CD38+ |
|  | CD8+ Eff CD45RO- CD27- |  |  | CD24+ CD38+ CD27- IgM+ Naive mature cells* |
|  | CD8+ Eff CD45RA+ CD27- |  |  | CD24+ CD38+ IgM- IgM- B cells |
|  | CD8+ CM CD45RO+ CD27+ |  |  | CD24+ CD38+ CD27+ IgM+ IgG- IgM only B cells |
|  | CD8+ CM CD45RA- CD27+ |  |  | CD24+ CD38+ CD27+ IgM+ IgG+ Natural effector B cells |
|  | CD45RO+ CD45RA+ T cells |  | IgD/IgM | IgD- IgM- |
|  | CD45RO+ CD45RA- T cells |  |  | IgD- IgM- CD27- |
|  | CD45RO- CD45RA+ T cells* |  |  | IgD- IgM- CD38++ plasmablasts |
|  | CD45RO- CD45RA- T cells |  |  | IgD- IgM- CD38+ CD27+ class switched memory B cells |
|  | CD4+ Naive CD45RO- CD27+* |  |  | IgD- IgM+ |
|  | CD4+ Naive CD45RA+ CD27+ |  |  | IgD- IgM+ CD27- |
|  | CD4+ EM CD45RO+ CD27-* |  |  | IgD- IgM+ CD27+ IgM only memory |
|  | CD4+ EM CD45RA- CD27- |  |  | IgD+ IgM+ |
|  | CD4+ Eff CD45RO- CD27- |  |  | IgD+ IgM+ CD27- Naive B cells |
|  | CD4+ Eff CD45RA+ CD27- |  |  | IgD+ IgM+ CD27+ Memory B cells |
|  | CD4+ CM CD45RO+ CD27+* |  |  | IgD+ IgM- |
|  | CD4+ CM CD45RA- CD27+ |  |  | IgM+ |
|  |  |  |  | IgM+ CD27- |
|  |  |  |  | IgM+ CD38++ CD27+ |
|  |  |  |  | IgM+ CD38+ CD27+ Class non switched memory |

*: Not determined in 300BCG

CM, central memory; DP, double positive; DN, double negative; EM, Effector memory; Eff, effector; Prol, proliferating; Tconv, conventional T cells; Treg, regulatory T cells

**Table S2, Cytokine production in *ex vivo* stimulation experiments in 500FG cohort**

| Cell system | | Macrophage | | PBMC | | | Whole Blood | | | | PBMC | | |
| --- | --- | --- | --- | --- | --- | --- | --- | --- | --- | --- | --- | --- | --- |
| Time of stimulation | | 1 day | | | | | 2 days | | | | 7 days | | |
| Cytokine | | IL-6 | TNF-α | IL-6 | TNF-α | IL-1β | IL-6 | TNF-α | IL-1β | IFN-γ | IL-17 | IL-22 | IFN-γ |
| Bacteria | B. burgdoferi |  |  | x | x | x |  |  |  |  | x | x | x |
|  | B. fragilis |  |  | x | x | x |  |  |  |  | x | x | x |
|  | E. coli |  |  | x | x | x |  |  |  |  |  |  |  |
|  | S. aureus |  |  | xx | xx | xx | x | x | x | x | x | x | xx |
|  | C. burnetii |  |  | x | x | x |  |  |  |  |  |  |  |
|  | M. tuberculosis | x | x | xx | xx | xx |  |  |  |  | x | x | xx |
|  | S. typhimurium | x | x |  |  |  |  |  |  |  |  |  |  |
| Fungi | A. fumigatus | x | x | x | x | x |  |  |  |  | x | x | x |
|  | C. albicans conidia | x | x | x | x | x | x | x | x | x | x | x | x |
|  | Cryptococcus |  |  | x | x | x |  |  |  |  | x | x | x |
| Virus | Influenza |  |  | x | x | x |  |  |  |  |  |  |  |
| TLR ligands | CpG |  |  | x | x | x |  |  |  |  |  |  |  |
|  | PolyIC |  |  | x | x | x |  |  |  |  |  |  |  |
|  | LPS | x | x | x | x | x | x | x | x | x |  |  |  |
|  | Pam3Cys |  |  | x | x | x |  |  |  |  |  |  |  |
| Non-microbial stimuli | MSU |  |  | x | x | x |  |  |  |  |  |  |  |
|  | MSU C16 |  |  | x | x | x |  |  |  |  |  |  |  |
|  | PHA |  |  |  |  |  | x | x | x | x |  |  |  |

x: cytokines determined only on the 500FG cohort; xx: cytokines determined in both the 500FG and 300BCG cohorts; CpG, short synthetic single-stranded DNA molecules containing unmethylated CpG dinucleotides; MSU, monosodium urate crystals; MSUC16, combination of monosodium urate crystals (MSU) and palmitic acid (C16:0); PH(A)phytohemagglutinin.

**Table S3, Comparative analysis of the steroid concentrations of the 500FG and 300BCG cohort.**

|  | 500FG | | | 300BCG | |
| --- | --- | --- | --- | --- | --- |
| Steroids | Male | Female-OCP | Female | Male | Female |
| n | 208 | 145 | 121 | 139 | 93 |
| Androstenedione (nmol) | 3.53 (2.57-4.65) ^#^ | 3.19 (2.30-4.26) *** | 4.06 (2.95-5.62) | 3.42 (2.75-4.66) ^####^ | 4.82 (3.29-5.99) |
| Cortisol (µmol) | 0.43 (0.35-0.53) | 0.97 (0.80-1.23) **** | 0.43 (0.33-0.50) | 0.40 (0.33-0.50) | 0.38 (0.28-0.50) |
| 11-deoxycortisol (nmol) | 0.95 (0.50-1.50) ^##^ | 0.8 (0.40-1.50) | 0.7 (0.40-1.30) | 0.90 (0.60-1.50) ^#^ | 0.70 (0.40-1.20) |
| 17-hydroxyprogesteron (nmol) | 2.93 (2.22-3.89) ^####^ | 0.64 (0.36-1.02) **** | 1.17 (0.72-2.29) | 2.87 (2.17-3.72) ^####^ | 1.25 (0.87-2.19) |
| Testosterone (nmol) | 18.55 (14.95-23.00) ^####^ | 0.77 (0.55-0.99) *** | 0.99 (0.75-1.30) | 16.7 (14.25-20.75) ^####^ | 0.89 (0.68-1.15) |
| Progesterone (nmol) | 0.25 (0.17-0.32) ^#^ | 0.22 (0.16-0.30) *** | 0.26 (0.16-0.80) |  |  |

Data are shown as median (25th-75th percentile).

* Female vs female taking OCP in the 500FG cohort, statistical were performed with Mann-Whitney test. *** *p* < 0.001, **** *p* < 0.0001.

# Male vs. female (not taking OCP) in both 500FG and 300BCG cohort, Mann-Whitney test. # *p* < 0.05, ## *p* < 0.01, #### *p* < 0.0001

**Table S4, Antibody information**

| **Antibody** | **Company** | **Catalog Number** | **Species** | **RRID** |
| --- | --- | --- | --- | --- |
| Alexa Fluor(R) 700 anti-human CD3 | BioLegend | 300424 | mouse | AB_493741 |
| FITC anti-human CD62L | BioLegend | 304804 | mouse | AB_314464 |
| IL-17A Monoclonal Antibody (eBio64DEC17), FITC | Thermo Fisher Scientific | 11-7179-82 | mouse | AB_763574 |
| PE anti-human CD127 (IL-7Ralpha) | BioLegend | 351304 | mouse | AB_2564136 |
| PE anti-human CD279 (PD-1) | BioLegend | 329906 | mouse | AB_940481 |
| PerCP/Cyanine5.5 anti-T-bet | BioLegend | 644806 | mouse | AB_1595593 |
| PE/Cyanine7 anti-human IFN-gamma | BioLegend | 506518 | mouse | AB_2123321 |
| APC anti-human CD274 | BioLegend | 329708 | mouse | AB_940360 |
| Brilliant Violet 510(TM) anti-human CD8 | BioLegend | 344732 | mouse | AB_2564623 |
| Brilliant Violet 650(TM) anti-human CD4 | BioLegend | 300536 | mouse | AB_2561351 |
| Brilliant Violet 785(TM) anti-human CD8 | BioLegend | 344739 | mouse | AB_2566202 |
| CD25 Monoclonal Antibody (BC96), PE-Cyanine7 | Thermo Fisher Scientific | 25-0259-42 | mouse | AB_1257140 |
| Hu ROR GMA T PE-CF594 Q21-559 50Tst | BD Biosciences | 567532 | mouse | AB_2916639 |
| Brilliant Violet 785(TM) anti-human CD183 (CXCR3) | BioLegend | 353738 | mouse | AB_2565924 |
