# Supplementary material for: 11-deoxycortisol positively correlates with T cell immune traits in physiological conditions
Source: eBioMedicine. 2023 Dec 21;99:104935. doi: 10.1016/j.ebiom.2023.104935 (PMC10776925; doi:10.1016/j.ebiom.2023.104935)
Supplement: 500FG study protocol [file mmc2.doc]

**500FG project**

**PROTOCOL TITLE: 500FG project, an explorative study**

| **Protocol ID** |  |
| --- | --- |
| **Short title** | 500FG project |
| **EudraCT number** | Not applicable |
| **Version** | 42561.091.12 |
| **Date** | March 13th 2013 |
| **Coordinating investigator/project leader** | Prof. Dr. M.G.Netea  Afd. Experimentele Interne Geneeskunde 463  RadboudUMC  Postbus 9101  6500 HB Nijmegen |
| **Principal investigator(s)** | Dr. Leo Joosten, Dr. S. Smeekens, Dr. M. Oosting, MSc. M. Jaeger  Afd. Experimentele Interne Geneeskunde 463  RadboudUMC  Postbus 9101  6500 HB Nijmegen |
| **Sponsor** | Prof. Dr. J. Smit  Afd. Experimentele Interne Geneeskunde 463  RadboudUMC  Postbus 9101  6500 HB Nijmegen |
| **Subsidising party** |  |
| **Independent expert (s)** |  |
|  |  |
|  |  |
| **Laboratory sites** | Laboratorium Algemeen Interne Geneeskunde (462),  RadboudUMC  Postbus 9101  6500 HB Nijmegen |

**Project group:**

**Afdeling Algemeen Interne Geneeskunde RadboudUMC** (Postbus 9101, 6500 HB Nijmegen, T:024-3618819)

- Prof. Dr. M.G. Netea: internist-infectioloog; E: [mihai.netea@radboudumc.nl](mailto:mihai.netea@radboudumc.nl)
- Dr. H. ter Hofstede: internist-infectioloog; E: Hadewych.terHofstede@cbg.umcn.nl
- Msc. A.Berende: internist-infectioloog i.o.; E: anneleen.berende@radboudumc.nl
- Dr. L.A.B. Joosten: E: [leo.joosten@radboudumc.nl](mailto:leo.joosten@radboudumc.nl)
- MSc. S.Smeekens; E: [sanne.smeekens@radboudumc.nl](mailto:sanne.smeekens@radboudumc.nl)
- MSc. M.Oosting; E: [marije.doppenberg-oosting@radboudumc.nl](mailto:marije.doppenberg-oosting@radboudumc.nl)
- MSc. M.Jaeger; [E: martin.jaeger@radboudumc.nl](mailto:E: martin.jaeger@radboudumc.nl)
- MSc. W.A. van der Heijden E: [wouter.vanderheijden@radboudumc.nl](mailto:wouter.vanderheijden@radboudumc.nl)
- Prof. dr A.J.A.M. van der Ven E:Andre,vanderven@radboudumc.nlProf. Dr J.W.M. van der Meer: E: [jos.vandermeer@radboudumc.nl](mailto:jos.vandermeer@radboudumc.nl)
- Prof. Dr. J.Smit: E: jan.smit@radboudumc.nl

**Afdeling Medische Microbiologie RadboudUMC** (Postbus 9101, 6500 HB Nijmegen, T: 024-3614338)

- Drs. C.J.M. Bartels

**Department of Laboratory Medicine–Laboratory of Medical Immunology RadboudUMC** (Postbus 9101, 6500 HB Nijmegen, T: 024-3616534)

- Dr. F. Preijers
- Dr. H. Koenen
- Prof. Dr. I. Joosten

**Center for Computational and Integrative Biology and Gastrointestinal Unit, Massachusetts General Hospital, Harvard School of Medicine, Boston, MA 02114; and** **Broad Institute of Massachusetts Institute of Technology and Harvard University, Cambridge, MA 02142** (T: (617) 724-6113)

- Dr. R. Xavier

**Genetics Department, University Medical Centre Groningen and University of Groningen** (Postbus 30.001, 9700 RB Groningen, T: 050 361 7100)

- Prof. Dr. C. Wijmenga

**PROTOCOL SIGNATURE SHEET**

| **Name** | **Signature** | **Date** |
| --- | --- | --- |
| **For non-commercial research,**  **Head of Department:**  Prof. Dr. J.W.M. van der Meer, MD PhD / Prof. Dr. J. Smit, MD PhD  Head of Department of Internal Medicine,  RadboudUMC |  |  |
| **Coordinating Investigator/Project leader:**  Prof. Dr. M.G. Netea, MD PhD  Department of Experimental Internal Medicine, RadboudUMC |  |  |
| **Principal investigator:**  Prof.Dr. L. Joosten, Dr. S.Smeekens, Dr. M.Oosting, M.Jaeger MSc.  Department of Internal Medicine, RadboudUMC |  |  |

**TABLE OF CONTEN**TS

[1. INTRODUCTION AND RATIONALE 11](#__RefHeading___Toc422301143)

[2. OBJECTIVES 13](#__RefHeading___Toc422301144)

[STUDY DESIGN 13](#__RefHeading___Toc422301145)

[2.1 DNA analysis 13](#__RefHeading___Toc422301146)

[2.2 Microbiome analysis 14](#__RefHeading___Toc422301147)

[2.3 Determining the phenotype and function of circulating immune cells 14](#__RefHeading___Toc422301148)

[2.4 Clinical parameters, biomarkers and questionnaires (for HIV cohort only) 16](#__RefHeading___Toc422301149)

[3. STUDY POPULATION 17](#__RefHeading___Toc422301150)

[Inclusion criteria 17](#__RefHeading___Toc422301151)

[3.1 Sample size calculation 18](#__RefHeading___Toc422301152)

[4. METHODS 19](#__RefHeading___Toc422301153)

[4.1 Study parameters/endpoints 19](#__RefHeading___Toc422301154)

[4.1.1 Main study parameter/endpoint 19](#__RefHeading___Toc422301155)

[4.2 Study procedures 19](#__RefHeading___Toc422301156)

[4.3 Withdrawal of individual subjects 19](#__RefHeading___Toc422301157)

[4.4 Replacement of individual subjects after withdrawal 19](#__RefHeading___Toc422301158)

[5. SAFETY REPORTING 21](#__RefHeading___Toc422301159)

[5.1 Section 10 WMO event 21](#__RefHeading___Toc422301160)

[5.1.1 Adverse events (AEs) 21](#__RefHeading___Toc422301161)

[6. STATISTICAL ANALYSIS 22](#__RefHeading___Toc422301162)

[7. ETHICAL CONSIDERATIONS 23](#__RefHeading___Toc422301163)

[7.1 Regulation statement 23](#__RefHeading___Toc422301164)

[7.2 Recruitment and consent 23](#__RefHeading___Toc422301165)

[7.3 Benefits and risks assessment, group relatedness 23](#__RefHeading___Toc422301166)

[7.4 Compensation for injury 24](#__RefHeading___Toc422301167)

[8. ADMINISTRATIVE ASPECTS, MONITORING AND PUBLICATION 25](#__RefHeading___Toc422301168)

[8.1 Handling and storage of data and documents 25](#__RefHeading___Toc422301169)

[8.2 Amendments 25](#__RefHeading___Toc422301170)

[8.3 Annual progress report 25](#__RefHeading___Toc422301171)

[8.4 End of study report 25](#__RefHeading___Toc422301172)

[8.5 Public disclosure and publication policy 25](#__RefHeading___Toc422301173)

[9. REFERENCES 26](#__RefHeading___Toc422301174)

**LIST OF ABBREVIATIONS AND RELEVANT DEFINITIONS**

| **ABR** | **ABR form, General Assessment and Registration form, is the application form that is required for submission to the accredited Ethics Committee (In Dutch, ABR = Algemene Beoordeling en Registratie)** |
| --- | --- |
| **AE** | **Adverse Event** |
| **AIDS** | **Acquired Immune Deficiency Syndrome** |
| **AR** | **Adverse Reaction** |
| **CA** | **Competent Authority** |
| **CCMO** | **Central Committee on Research Involving Human Subjects; in Dutch: Centrale Commissie Mensgebonden Onderzoek** |
| **CD** | **Cluster of Differentiation** |
| **CV** | **Curriculum Vitae** |
| **DNA** | **Desoxyribo Nucleic Acid** |
| **DSMB** | **Data Safety Monitoring Board** |
| **EDTA** | **Ethylenediaminetetraacetic acid** |
| **EU** | **European Union** |
| **EudraCT** | **European drug regulatory affairs Clinical Trials** |
| **FACS** | **Fluorescent Activated Cell Sorter** |
| **GCP** | **Good Clinical Practice** |
| **HIV** | **Human Immunodeficiency Virus** |
| **IB** | **Investigator’s Brochure** |
| **IC** | **Informed Consent** |
| **IL** | **Interleukin** |
| **IMP** | **Investigational Medicinal Product** |
| **IMPD** | **Investigational Medicinal Product Dossier** |
| **LPS** | **Lipopolysaccharide** |
| **METC** | **Medical research ethics committee (MREC); in Dutch: medisch ethische toetsing commissie (METC)** |
| **MNC** | **Mono Nuclear Cells** |
| **Mono** | **Monocyte** |
| **MSU** | **Mono Sulfate Urate** |
| **MØ** | **Macrophage** |
| **PBMC** | **Peripheral Blood Mononuclear Cell** |
| **PHA** | **phytohemagglutinin** |
| **RNA** | **Ribonucleic acid** |
| **RPMI** | **Roswell Park Memorial Institute** |
| **RUMC** | **Radboud University Medical Center** |
| **RVVC** | **Recurrent Vulvo vaginal candidemia** |
| **(S)AE** | **(Serious) Adverse Event** |
| **SNP** | **Single Nucleotide Polymorphism** |
| **SPC** | **Summary of Product Characteristics (in Dutch: officiële productinfomatie IB1-tekst)** |
| **Sponsor** | **The sponsor is the party that commissions the organisation or performance of the research, for example a pharmaceutical**  **company, academic hospital, scientific organisation or investigator. A party that provides funding for a study but does not commission it is not regarded as the sponsor, but referred to as a subsidising party.** |
| **SUSAR** | **Suspected Unexpected Serious Adverse Reaction** |
| **Wbp** | **Personal Data Protection Act (in Dutch: Wet Bescherming Persoonsgevens)** |
| **WMO** | **Medical Research Involving Human Subjects Act (in Dutch: Wet Medisch-wetenschappelijk Onderzoek met Mensen** |

**SUMMARY**

**Rationale:** The response of the host to exogenous (e.g. infectious) or endogenous (e.g. metabolic) stressors depends on the genetic make-up of the host on the one hand, and environmental factors on the other hand. One of the most important environmental components that influences human physiological responses is the colonizing microbial flora. In a healthy human body, more microbial cells are present on the skin and mucosae (e.g. oral, gut, vagina) than normal human cells. Due to the important effects of the colonizing microflora for multiple biological processes (e.g. host defense, digestion, etc), a finely tuned balance between the microorganisms that form the microbiome and the host is very important for the maintenance of health. This balance might be disturbed in people suffering from chronic inflammatory diseases, infections or metabolic diseases.

It has been recently hypothesized that these two factors, genetic and environmental (in this case the microbiome), strongly influence each other and the immune system of the host. Furthermore, it has been demonstrated that microbiome composition can change over time and seasonal changes have been reported .In this respect, the interaction between the genome, the microbiome and the immune response becomes crucial for the health status of an individual and for the development of disease. However, a comprehensive analysis of the genome-microbiome-host defense interaction was never performed. Moreover, it is not known how this interaction is affected in patients with infections or inflammatory diseases.

**Objective**: To characterize the interaction between the genetic background, the microbiome, and the immune responses in healthy individuals, both cross-sectional and over time, and to identify the disturbances in this interaction in patients with specified infections (candidiasis, Lyme disease, HIV) or inflammatory diseases (gout).

**Study design:** The explorative study will be performed in the RadboudUMC The duration of the study is 3 years. This explorative study starts with recruiting healthy individuals, of which a subgroup (n=50) will be measured in total 4 times, whereafter also patients with several diseases will be recruited at the RadboudUMC. We will use several approaches to investigate the above-described factors:

1. Metadata will be collected from all the participants using standard questionnaires on lifestyle. These questionnaires will be coded with full respect for the privacy of the individuals, will be anonymous, and will not permit the identification of the individuals. In case of the patients, clinical data (per type of pathology) will be collected.
2. DNA will be isolated using 1 ml of whole blood. Gene polymorphisms will be assessed in the DNA samples of patients or in healthy individuals.
3. Microbiome analysis will be performed on stool, oral, vaginal, and skin samples.
4. The function of the immune system will be analysed at several levels using circulating cells from venous blood: immunophenotyping will be done using FACS analysis, circulating factors will be measured in plasma or serum, in-vitro stimulations of cells and analysis of mRNA and cytokine responses.

**Study population:**

Cohorts of healthy controls and patients:

- Healthy individuals (n=450)
- Healthy individuals (n=50) for 4 repeated measurements
- Recurrent Vulvo-Vaginal Candidemia (RVVC) patients (n=200)
- Candidemia patients (n=50)
- Lyme disease patients (n=150)
- Gout patients (n=200)
- HIV-infected patients (n=400)

**Main study parameters/endpoints:**

1. Genetic variations that influence the innate and adaptive immune response genes will be assessed in DNA samples of healthy individuals by systems biology and pathway analysis approaches. The identified polymorphisms in this first step will be thereafter tested to assess whether they influence susceptibility to the diseases assessed here.
2. The microbiome will be compared between healthy individuals and patients, and the influence of the genetic background of the host on microbial composition will be assessed.
3. The correlation of the microbiome (classes of colonizing microorganisms) with the phenotype of immune responses will be assessed: immunophenotyping of both healthy individuals and patients, circulating factors, innate and adaptive ex-vivo responses.
4. The composition of the microbiome will be monitored over time (4 times, over the course of 12 months in order to cover all seasons ), To investigate how the microbiome is influenced by external factors like seasonal influences, diet, etc, and to what extent changes in microbial composition influence the immune response.

**Nature and extent of the burden and risks associated with participation, benefit and group relatedness:**

Burden:

- For patients and controls: collection of extra blood, when possible during regular blood sampling. This comprises a maximum of 1 PaxGene tube of 8 mL, 1 heparin tube of 10 ml, 6 EDTA tubes of 10 ml, 1 citrate tube of 3 ml and 1 serum tube of 5 ml (total amount 86 mL) and 1 additional citrate tube for HIV patients (total amount 89 mL)
- Collection of stool, urine, oral smear, and skin smear samples for both patients and controls.
- For women: smear of the vaginal area for vaginal microbiome (will be performed by the participant herself using a provided kit).
- For HIV patients and 50 out of the 500 healthy volunteers: self-report questionnaires on neuropsychiatric symptoms (20-30minutes).

Risks:

- No risks other than local hematoma related to a single venous puncture.

Benefit:

- There will be no direct benefits for the subjects enrolled in this study.

# INTRODUCTION AND RATIONALE

Several factors have been demonstrated to date to have a crucial effect on the susceptibility and outcome of infections and (auto)inflammatory diseases: (1) the genetic make-up of the individual, (2) the colonizing microorganisms on its skin and mucosae, and (3) variations in the immune responses.

(1) The knowledge about DNA polymorphisms has become a valuable parameter for assessing risks to develop many important human diseases. Recently, we (and others) have demonstrated that polymorphisms in immunity-related genes lead to differences in susceptibility to fungal diseases , bacterial infections such as *Borrelia* or *Bacteroides* , arthritis , or autoimmune disorders such as inflammatory bowel disease (i.e. Crohn’s disease) .

(2) Next to differences in DNA patterns, it could also be demonstrated that the presence of certain microorganisms in the gut (e.g. probiotic bacteria) are able to influence the cytokine production and possible clinical outcome . All vertebrates display complex communities of microorganisms on body surfaces, called the microbiome or microbiota. However, most of these microbes are not cultivable and therefore 16S sequence analysis has proven to give crucial information regarding the complexity of microbial communities on the skin and mucosae . It has been demonstrated that the microbiome can change over time and that there is a continuous turnover of the microbial composition . The knowledge about the variation in the human gut will be increased in a second project called the Human Microbiome project . A search on PubMed indicates the exponential growth of this discipline (0 in 1999 against 865 articles in 2011). The microbiome was demonstrated to give valuable information about the origin, lifestyle, development of immune responses, infections, metabolic and autoimmune diseases . Next to microbial patterns in the gut, urine and skin samples were also described to give valuable information in health and disease . It could be demonstrated that the microbial components present in the human body can be related to immune responses or autoinflammatory disorders . Therefore, it is plausible to link the differences in microbial status and illnesses .

(3) Next to the presence or absence of certain groups of microbes in disease, the phenotype and function of circulating cells might also differ between health and disease. In HIV/AIDS patients is the best example described, a typical trait of this disease is a decreased number of CD4+ T cells in the circulation. Next to HIV/AIDS, CD4+ T cell numbers were also described to differ in certain stages of tuberculosis , arthritis and Crohn’s disease . Next to T cells, other cell types, such as dendritic cells, may also change their phenotypic characteristics during disease .

The capacity of immune cells to produce cytokines and chemokines has proven to be highly important in health and disease. Differences in cytokine production were linked to several diseases, including Lyme disease , Crohn’s disease , but also fungal infections with for example candida . Individuals with altered cytokine responses were reported to be highly susceptible for the development of disease.

The introduction of combination antiretroviral therapy (cART) has substantially improved the life expectancy of HIV-infected patients . Nevertheless, chronic HIV-infection, despite a suppressed viral load, is associated with increased risk for cardiovascular disease, neuropsychiatric and neurocognitive disorders and cancer . These long-term complications of HIV are thought to arise from persisting immune activation , a process which may in part be maintained by HIV-associated microbial translocation . Small microbiome studies have been conducted in HIV patients , but an integrative view of host genome, microbiome, systemic immune activation and clinical parameters are lacking in HIV infected individuals.

Despite the research that has linked each of these processes to the susceptibility to disease, practically nothing is known how they influence each other and how does this interaction relate to the various diseases. In other words, it is not known whether genetic polymorphisms influence the composition of the microbiome, how these two factors together modify the immune response of the host, and what are the consequences for the susceptibility to infections or (auto)inflammatory disorders. This is the aim of the recently awarded ERC-Consolidator Grant (nr.310372) to M.G. Netea, which stays at the core of this study.

Therefore, we will assess by systems biology and pathway analysis the interaction between the DNA polymorphisms on the one hand, the microbiome of the skin, intestinal tract and vagina, and the immune responses in a large group of healthy individuals. After assessing the interaction of these factors in healthy controls, we will assess whether this balance is distorted in certain patients groups with infections or (auto)inflammatory disorders.

# OBJECTIVES

The aim of this explorative study is to test the hypothesis that susceptibility to and severity of certain infectious and inflammatory diseases can be explained by the interaction between the genome, microbiome and immunological responses: i.e. presence of polymorphisms, differences in microbial composition, differences in phenotypes and/or of circulating cells (altered cytokine production).

Specific research questions are:

1. Do genetic variations influence the immune responses and subsequently the susceptibility or severity of disease?
2. Is there a difference in the composition of the microbiome between healthy controls and patient groups?
3. Is the colonization with certain classes of microorganisms influenced by genetic polymorphisms of the host?
4. What is the phenotype and function of the circulating cells in patients and controls, and is that influenced by the gene polymorphisms on the one hand, and microbiome on the other hand?

# STUDY DESIGN

The duration of the study is 3 years. This study has an explorative design. The study starts with recruiting healthy individuals after which patients with several diseases will also be recruited at the Radboudumc. The study will be performed in the Radboudumc, in collaboration with Harvard School of Medicine and UMC Groningen. Patients will be recruited from the Radboudumc.

## DNA analysis

In this part of the study all individuals that have been included will be studied. The study comprises:

1. Healthy individuals (n=450) (control)
2. Healthy individuals (n=50) (control) for repeated measurements
3. RVVC patients (n=200)
4. Candidemia patients (n=50)
5. Patients diagnosed with Lyme disease (n=150)
6. Gout patients (n=200)
7. HIV-infected patients (n=400)

DNA samples will be obtained from EDTA blood collected from healthy individuals and patients; the rest of the EDTA tube will be used for analysis of circulating mediators in the plasma.

Common genetic variations (single nucleotide polymorphisms (SNPs)) will be determined in DNA by immunochip arrays (ref. Smeekens et al, Nature Communications, 2013). The selection of these polymorphisms is based on the following criteria: the polymorphisms have a minor allele frequency of at least 5% and the polymorphisms have known functional effects or have previously been found to be associated with susceptibility to infectious diseases. Quality of genotype data will be verified by genotyping ten percent of the samples in duplo, as well as sequencing several samples for each polymorphism that proves to influence the function of the immune system, in order to verify genotype assignment.

## Microbiome analysis

Microbiome analysis will be performed using 16S sequencing and SLiME software package. The analysis will be performed in stool samples collected by the healthy individuals and patients themselves using sterile containers provided by the RUMC. Oral smears, vaginal smears, and skin smears will be also collected in swap containers provided by the RUMC. Urine will be collected by the participants in sterile urine containers. All samples will be stored at -80°C until analysis.

## Determining the phenotype and function of circulating immune cells

Immunophenotyping of ciroculating cell populations will be measured using FACS analysis (e.g. CD3/CD4/CD8 for T-lymphocytes; CD19/CD20 for B-cells; CD14 for monocytes, CD16/CD56 for NK-cells) in whole ACDA blood at LABGK, LMI. mRNA expression will be measured in whole blood collected in PaxGene tubes (BD/Qiagen). Circulating mediators (cytokines, acute phase proteins, etc) will be measured in plasma and/or serum samples.

Ex-vivo stimulation experiments (only in controls) will assess cytokine production by primary blood leukocytes:

i. The in-vitro immune response will be determined in single samples of whole blood stimulated for 48 hours with RPMI, LPS, PHA, *Candida*, and *Staphylococcus*.

ii. To explore the cytokine production by PBMCs, cells will be stimulated according to the table below.

| **Stimulation of PBMCs 24 hours** | **Stimulation of PBMCs for 24 hours and 7 days** |
| --- | --- |
| **LPS low concentration** | RPMI |
| **LPS high concentration** | *Borrelia burgdorferi* |
| **Pam3Cys** | Borrelia mix (*B.b./B.a./B.g.*) |
| **Flagellin** | *Candida conidia* |
| **Poly I:C** | *Candida hyphae* |
| **CpG** | *Aspergillus conidia* |
| **R848** | *Aspergillus hyphae* |
| **β-glucan** | *Mycobacterium tuberculosis* |
| **β-glucan + Pam3Cys** | *Mycobacterium avium* |
| **MSU** | *Staphylococcus aureus* |
| **MSU + C16** | *Cryptococcus* |
| **E.coli** | *Bacteroides fragilis* |

The stimulation experiments will be performed at the laboratory of Experimental Medicine at the RadboudUMC. Within 2-4 hours, the heparin blood will be used for whole blood stimulation with the stimuli mentioned above. From the EDTA blood, cells will be isolated within 2-4 hours after blood sampling. Cells to be used are mononuclear cells (MNCs) and in-vitro differentiated monocyte-derived macrophages (MDMs). The cells will be exposed to the stimuli mentioned in the table above. The function of the various pattern recognition receptors will be tested by different receptor-specific ligands.

50 out of the total 500 healthy volunteers will be asked to give blood and microbiome samples 4 times in total, with intervals of 3 months. These 50 individuals also receive a questionnaire after each visit. During the last visit, participants will be asked to complete a self-reported neuropsychiatric questionnaire, which consists of the DASS, BIS-11 and part of the MATE (see 2.4 Clinical parameters, biomarkers and questionnaires).

## Clinical parameters, biomarkers and questionnaires (for HIV cohort only)

# Clinical parameters will be collected from the hospital information system (EPIC (eg. HIV-load, CD4 count). Biomarkers for cardiovascular disease (e.g. platelet function, coagulation parameters and lipid profile) and neuropsychiatric disease (e.g. tryptophan metabolites) will be measured. Additionally, patients are asked to complete a self-reported neuro-psychiatric questionnaire (a combination of the “Depression Anxiety Stress Scales (DASS)” , “Barratt Impulsiveness Scale-11 (BIS-11)”, and a part of the “Meten van Addicties voor Triage en Evaluatie (MATE)”).

# STUDY POPULATION

Volunteers will be actively recruited using flyers and posters. Blood will be drawn at the outpatient clinic of the RUNMC after obtaining written informed consent. All healthy subjects should be of Caucasian origin with the Dutch nationality. The gender distribution of the cohort should be around 50% males and 50% females. 50 volunteers that have indicated that they want to be contacted for future research will be invited through e-mail to participate a second, third and fourth time.

## Inclusion criteria

**Healthy individuals (n=500)**

In order to be eligible to participate in this study, all subjects must meet all of the following criteria:

- older than 18 years
- no pregnancy (for women)
- no chronic or acute disease at the time of assessment
- no use of chronic or acute medication during the last month before the study

Inclusion criteria for the patient groups:

**RVVC patients (n=200)**

- healthy women, age >18 years
- no pregnancy, no diabetes, no antibiotic use
- at least 3 episodes of vulvo-vaginal candidiasis per year, microbiologically confirmed

**Candidemia patients (n=50)**

- patient with blood culture positive for a *Candida* species
- age > 18 years
- treatment given longer than 24h before sample collection

**Lyme disease patients (n=150)**

- age > 18 years old
- clinical diagnosis based on erythema migrans.
- positive serology (IgG) for *Borrelia*.

**Gout patients (n=200)**

- age > 18 years
- diagnosis based on clinical criteria and positive urate crystals in the joint fluid

**HIV-infected patients (n=400)**

- age > 18 years
- documented HIV infectioncaucasian
- no opportunistic infection at the time of sampling
- no use of antibiotics in the last month
- no active hepatitis B or C
- in females, no pregnancy
- viral load <200 copies/mL

## Sample size calculation

The sample size calculation is not possible to be provided due to the variable frequencies of the various traits in genome-microbiome interaction (SNP frequency, variable prevalence of specific microbial genera and species, etc), and their effect on cytokine production. As a result of this explorative study, the size calculation will be variable depending on the type of polymorphism analyzed. The frequencies of the various microorganism classes in the colonizing microbiome is not known in our populations, and therefore power calculations are impossible to be performed.

Earlier studies in the Human Microbiome project have assessed microbiome traits in 250 individuals . In earlier studies by our group, the associations between genome-immunity have been reliable obtained in a group of approximately 100 volunteers . Because we intend to add an additional aspect to this analysis, and assess the interaction between genome-microbiome-immunity, after discussion with our colleagues performing the biostatistics analysis at the Broad Institute at MIT and Harvard (group of Prof. Ramnik Xavier), we decided to increase the number of individuals tested to 500.

The following populations will be used in this explorative study:

- Healthy individuals (n=450) plus 50 which will be measured longitudinally.
- Recurrent Vulvo-Vaginal Candidemia (RVVC) patients (n=200)
- Candidemia patients (n=50)
- Lyme disease patients (n=150)
- Gout patients (n=200)

HIV-infected patients (n=400)

# METHODS

## Study parameters/endpoints

### Main study parameter/endpoint

Metadata: Lifestyle questionnaires Neuropsychiatric questionnaire (HIV cohort only)

DNA: Gene polymorphisms at DNA level

Microbiome: Presence of groups of bacteria

Phenotype: Specific populations of cells

Functional data: Cytokine productionBiomarkers Cardiovascular disease (e.g. platelet function, lipid profile), neuropsychiatric disease (e.g. tryptophan metabolites) (HIV cohort only).

Clinical data: e.g. CD4 nadir, viral load, ART (patient cohorts only)

## Study procedures

Metadata: Lifestyle forms, excel for analysis, statistical programs Neuropsychiatric questionnaire (combination of DASS, BIS-11 and part of MATE (HIV cohort only)

DNA: DNA isolation kit (Qiagen),sequencing machine, real-time PCR

Microbiome: 16S sequencing and SLiMe software analysis

Phenotype: FACS-machine

Functional data: PBMC isolation, ELISA/Luminex measurement

Biomarkers: High-performance liquid chromatography

Clinical data: Medical record abstraction from EPIC (patient cohorts only)

For the participants: a single collection of venous blood is the only invasive procedures to be performed

## Withdrawal of individual subjects

Subjects can leave the study at any time for any reason if they wish to do so without any consequences. The investigator can decide to withdraw a subject from the study for urgent medical reasons.

## Replacement of individual subjects after withdrawal

If subject left the study, all information will be discarded.

## Storage of materials

Materials collected from the participants will be stored indefinitely in the Biobank from the Radboudumc Nijmegen, unless the participant refuses to store the materials. This is a question in the informed consent form. Material will be discarded when the participant disagrees with storage of the materials. Left over material in other centers (Harvard, Groningen) will be sent back to Nijmegen and stored in the Biobank. Material is stored anonymously as coded as described before. The main researchers have access to the code sample list.

Re-use of the samples of participants is only possible when the participant agrees with re-use as mentioned in the informed consent form.

# SAFETY REPORTING

## Section 10 WMO event

In accordance to section 10, subsection 1, of the WMO, the investigator will inform the subjects and the reviewing accredited METC if any adverse effect occurs, on the basis of which it appears that the disadvantages of participation may be significantly greater than was foreseen in the research proposal. The study will be suspended pending further review by the accredited METC, except insofar as suspension would jeopardise the subjects’ health. The investigator will take care that all subjects are kept informed.

### Adverse events (AEs)

- Adverse events are not expected during this study
- No risks other than minor local hematoma related to venous puncture.

# STATISTICAL ANALYSIS

The primary analysis of this explorative study will be to determine differences in terms of cytokine profiles together with genetic variations, microbiome variations, cell phenotypes, and RNA differences in in-vitro stimulated cells of controls versus patients. For quantitatively comparing cytokine production we will employ the non-parametric Mann-Whitney U for unpaired continuous variables to analyze comparisons for statistical significant differences. GraphPad PRISM software will be used for the calculations. Results will be expressed as means + standard errors of the means (SEM). P values less than .05 are considered statistically significant.

To determine differences in prevalence of genetic polymorphisms genotype data will be analyzed using haploview software. Statistical analyses will be performed in collaboration with the group of Prof. C. Wijmenga (Department of Genetics, UMCG) and the group of Prof. Ramnik Xavier (Broad Institute at MIT and Harvard). A log2-transformation and quantile normalization will be performed. Quantile normalization is useful for normalizing across a series of conditions. A Kruskal-Wallis test will be performed on the normalized data.

Microbiome data will be analyzed using SLiME software analysis, in collaboration with Dr. D. Gevers (Human Microbiome Project, Broad Institute at MIT and Harvard, Boston, USA). The associations between genome-microbiome-immune response will be performed by systems biology and pathway analysis, in collaboration with Prof. R Xavier (Harvard University and Broad Institute at MIT, Boston, Massachussetts).

# ETHICAL CONSIDERATIONS

## Regulation statement

The study will be conducted according to the principles of the Declaration of Helsinki (newest version) and in accordance with the Medical Research Involving Human Subjects Act (WMO). The study will start after approval by the Ethics Review Board (CMO) region Arnhem-Nijmegen.

## Recruitment and consent

All patients from whom blood is obtained receive the information letter by mail before the visit to the outpatient clinic of the Department of Medicine, RadboudUMC. Written informed consent will then be obtained during the visit to the outpatient clinic. Additional explanation about the study can then also be given by the researcher. Healthy volunteers for the study will be recruited by advertisement on posters and flyers.

## Benefits and risks assessment, group relatedness

The study population consists of capacitated adults. Minors and incapacitated subjects will be excluded.

Burden:

- For patients and controls: collection of venous blood, if possible during regular blood sampling. This comprises a maximum of 6 EDTA tubes à 10 ml, 1 heparin tube à 10 ml, 1 PaxGene tube a 8 mL, and 1 citrate tube a 3 mL and 1 serum tube à 5 ml (total amount 86mL) and 1 additional citrate tube for HIV patients (total amount 89 mL)
- Collection of stool, urine, oral smear, and skin smear samples for both patients and controls.
- For women: smear of the vaginal area for vaginal microbiome (will be performed by the participant herself using a provided kit).
- For HIV patients and 50 out of the 500 healthy volunteers: self-report questionnaires on neuropsychiatric symptoms (30-45 minutes).

Risks:

- No risks other than local hematoma are related to venous puncture.

Benefit:

There will be no benefits for the subjects enrolled in this study.

## Compensation for injury

Participants in this study will receive 50 Euro. Participants that are enrolled for the longitudinal study will receive 50 Euros for each time they participate during the inclusion period.

# ADMINISTRATIVE ASPECTS, MONITORING AND PUBLICATION

## Handling and storage of data and documents

All subject data will be handled anonymously. The investigator will record all data, including clinical data of the patients in a database in which patients are anonymously coded. Only the main researcher will have access to the patient identifying data.

## Amendments

Amendments are changes made to the research after a favourable opinion by the accredited METC has been given.

All amendments will be notified to the METC that gave a favourable opinion.

## Annual progress report

The sponsor/investigator will submit a summary of the progress of the trial to the accredited METC once a year. Information will be provided on the date of inclusion of the first subject, numbers of subjects included and numbers of subjects that have completed the trial, serious adverse events/ serious adverse reactions, other problems, and amendments.

## End of study report

The investigator will notify the accredited METC of the end of the study within a period of 8 weeks. The end of the study is defined as the last patient’s last visit.

In case the study is ended prematurely, the investigator will notify the accredited METC, including the reasons for the premature termination.
Within one year after the end of the study, the investigator/sponsor will submit a final study report with the results of the study, including any publications/abstracts of the study, to the accredited METC.

## Public disclosure and publication policy

The study results, both positive and negative, will be submitted for publication to peer reviewed journals. The investigators will hold final responsibility for the decision to submit the publication.

# REFERENCES

1. Davenport, E. R., O. Mizrahi-Man, K. Michelini, L. B. Barreiro, C. Ober, and Y. Gilad. 2014. Seasonal variation in human gut microbiome composition. *PloS one* 9: e90731.

2. Faith, J. J., J. L. Guruge, M. Charbonneau, S. Subramanian, H. Seedorf, A. L. Goodman, J. C. Clemente, R. Knight, A. C. Heath, R. L. Leibel, M. Rosenbaum, and J. I. Gordon. 2013. The long-term stability of the human gut microbiota. *Science* 341: 1237439.

3. Grice, E. A., H. H. Kong, S. Conlan, C. B. Deming, J. Davis, A. C. Young, N. C. S. Program, G. G. Bouffard, R. W. Blakesley, P. R. Murray, E. D. Green, M. L. Turner, and J. A. Segre. 2009. Topographical and temporal diversity of the human skin microbiome. *Science* 324: 1190-1192.

4. Ursell, L. K., J. C. Clemente, J. R. Rideout, D. Gevers, J. G. Caporaso, and R. Knight. 2012. The interpersonal and intrapersonal diversity of human-associated microbiota in key body sites. *The Journal of allergy and clinical immunology* 129: 1204-1208.

5. Smeekens, S. P., T. S. Plantinga, F. L. van de Veerdonk, B. Heinhuis, A. Hoischen, L. A. Joosten, P. D. Arkwright, A. Gennery, B. J. Kullberg, J. A. Veltman, D. Lilic, J. W. van der Meer, and M. G. Netea. 2011. STAT1 hyperphosphorylation and defective IL12R/IL23R signaling underlie defective immunity in autosomal dominant chronic mucocutaneous candidiasis. *PloS one* 6: e29248.

6. Oosting, M., H. ter Hofstede, F. L. van de Veerdonk, P. Sturm, B. J. Kullberg, J. W. van der Meer, M. G. Netea, and L. A. Joosten. 2011. Role of interleukin-23 (IL-23) receptor signaling for IL-17 responses in human Lyme disease. *Infection and immunity* 79: 4681-4687.

7. Stappers, M. H., N. A. Janssen, M. Oosting, T. S. Plantinga, P. Arvis, J. W. Mouton, L. A. Joosten, M. G. Netea, and I. C. Gyssens. 2012. A role for TLR1, TLR2 and NOD2 in cytokine induction by Bacteroides fragilis. *Cytokine*.

8. Plantinga, T. S., J. Fransen, N. Takahashi, R. Stienstra, P. L. van Riel, W. B. van den Berg, M. G. Netea, and L. A. Joosten. 2010. Functional consequences of DECTIN-1 early stop codon polymorphism Y238X in rheumatoid arthritis. *Arthritis research & therapy* 12: R26.

9. Plantinga, T. S., T. O. Crisan, M. Oosting, F. L. van de Veerdonk, D. J. de Jong, D. J. Philpott, J. W. van der Meer, S. E. Girardin, L. A. Joosten, and M. G. Netea. 2011. Crohn's disease-associated ATG16L1 polymorphism modulates pro-inflammatory cytokine responses selectively upon activation of NOD2. *Gut* 60: 1229-1235.

10. Brorsson, C. A., S. Onengut, W. M. Chen, J. Wenzlau, L. Yu, P. Baker, A. J. Williams, P. J. Bingley, J. C. Hutton, G. S. Eisenbarth, P. Concannon, S. S. Rich, F. Pociot, and C. Type 1 Diabetes Genetics. 2015. Novel association between immune-mediated susceptibility loci and persistent autoantibody positivity in type 1 diabetes. *Diabetes*.

11. Plantinga, T. S., W. W. van Maren, J. van Bergenhenegouwen, M. Hameetman, S. Nierkens, C. Jacobs, D. J. de Jong, L. A. Joosten, B. van't Land, J. Garssen, G. J. Adema, and M. G. Netea. 2011. Differential Toll-like receptor recognition and induction of cytokine profile by Bifidobacterium breve and Lactobacillus strains of probiotics. *Clinical and vaccine immunology : CVI* 18: 621-628.

12. Handelsman, J. 2004. Metagenomics: application of genomics to uncultured microorganisms. *Microbiology and molecular biology reviews : MMBR* 68: 669-685.

13. Gevers, D., R. Knight, J. F. Petrosino, K. Huang, A. L. McGuire, B. W. Birren, K. E. Nelson, O. White, B. A. Methe, and C. Huttenhower. 2012. The human microbiome project: a community resource for the healthy human microbiome. *PLoS biology* 10: e1001377.

14. Alan, G., and J. P. Sarah. 2012. Microbes as forensic indicators. *Tropical biomedicine* 29: 311-330.

15. Vaarala, O. 2013. Human intestinal microbiota and type 1 diabetes. *Current diabetes reports* 13: 601-607.

16. Fouts, D. E., R. Pieper, S. Szpakowski, H. Pohl, S. Knoblach, M. J. Suh, S. T. Huang, I. Ljungberg, B. Sprague, S. K. Lucas, M. Torralba, K. E. Nelson, and S. L. Groah. 2012. Integrated next-generation sequencing of 16S rDNA and metaproteomics differentiate the healthy urine microbiome from asymptomatic bacteriuria in neuropathic bladder associated with spinal cord injury. *Journal of translational medicine* 10: 174.

17. Naik, S., N. Bouladoux, C. Wilhelm, M. J. Molloy, R. Salcedo, W. Kastenmuller, C. Deming, M. Quinones, L. Koo, S. Conlan, S. Spencer, J. A. Hall, A. Dzutsev, H. Kong, D. J. Campbell, G. Trinchieri, J. A. Segre, and Y. Belkaid. 2012. Compartmentalized control of skin immunity by resident commensals. *Science* 337: 1115-1119.

18. Hooper, L. V., D. R. Littman, and A. J. Macpherson. 2012. Interactions between the microbiota and the immune system. *Science* 336: 1268-1273.

19. Morgan, X. C., T. L. Tickle, H. Sokol, D. Gevers, K. L. Devaney, D. V. Ward, J. A. Reyes, S. A. Shah, N. Leleiko, S. B. Snapper, A. Bousvaros, J. Korzenik, B. E. Sands, R. J. Xavier, and C. Huttenhower. 2012. Dysfunction of the intestinal microbiome in inflammatory bowel disease and treatment. *Genome biology* 13: R79.

20. Vaziri, N. D., J. Wong, M. Pahl, Y. M. Piceno, J. Yuan, T. Z. Desantis, Z. Ni, T. H. Nguyen, and G. L. Andersen. 2012. Chronic kidney disease alters intestinal microbial flora. *Kidney international*.

21. Streitz, M., S. Fuhrmann, D. Thomas, E. Cheek, L. Nomura, H. Maecker, P. Martus, N. Aghaeepour, R. R. Brinkman, H. D. Volk, and F. Kern. 2012. The phenotypic distribution and functional profile of tuberculin-specific CD4 T-cells characterizes different stages of TB infection. *Cytometry. Part B, Clinical cytometry*.

22. Omoyinmi, E., R. Hamaoui, A. Pesenacker, K. Nistala, H. Moncrieffe, S. Ursu, L. R. Wedderburn, and P. Woo. 2012. Th1 and Th17 cell subpopulations are enriched in the peripheral blood of patients with systemic juvenile idiopathic arthritis. *Rheumatology* 51: 1881-1886.

23. Reikvam, D. H., G. Perminow, L. G. Lyckander, J. M. Gran, P. Brandtzaeg, M. Vatn, and H. S. Carlsen. 2011. Increase of regulatory T cells in ileal mucosa of untreated pediatric Crohn's disease patients. *Scandinavian journal of gastroenterology* 46: 550-560.

24. Kramer, M., M. G. Netea, D. J. de Jong, B. J. Kullberg, and G. J. Adema. 2006. Impaired dendritic cell function in Crohn's disease patients with NOD2 3020insC mutation. *Journal of leukocyte biology* 79: 860-866.

25. Plantinga, T. S., W. J. van der Velden, B. Ferwerda, A. B. van Spriel, G. Adema, T. Feuth, J. P. Donnelly, G. D. Brown, B. J. Kullberg, N. M. Blijlevens, and M. G. Netea. 2009. Early stop polymorphism in human DECTIN-1 is associated with increased candida colonization in hematopoietic stem cell transplant recipients. *Clinical infectious diseases : an official publication of the Infectious Diseases Society of America* 49: 724-732.

26. 2000. Survival after introduction of HAART in people with known duration of HIV-1 infection. The CASCADE Collaboration. Concerted Action on SeroConversion to AIDS and Death in Europe. *Lancet* 355: 1158-1159.

27. Rabkin, J. G. 2008. HIV and depression: 2008 review and update. *Current HIV/AIDS reports* 5: 163-171.

28. Guaraldi, G., G. Orlando, S. Zona, M. Menozzi, F. Carli, E. Garlassi, A. Berti, E. Rossi, A. Roverato, and F. Palella. 2011. Premature age-related comorbidities among HIV-infected persons compared with the general population. *Clinical infectious diseases : an official publication of the Infectious Diseases Society of America* 53: 1120-1126.

29. Freiberg, M. S., C. C. Chang, L. H. Kuller, M. Skanderson, E. Lowy, K. L. Kraemer, A. A. Butt, M. Bidwell Goetz, D. Leaf, K. A. Oursler, D. Rimland, M. Rodriguez Barradas, S. Brown, C. Gibert, K. McGinnis, K. Crothers, J. Sico, H. Crane, A. Warner, S. Gottlieb, J. Gottdiener, R. P. Tracy, M. Budoff, C. Watson, K. A. Armah, D. Doebler, K. Bryant, and A. C. Justice. 2013. HIV infection and the risk of acute myocardial infarction. *JAMA internal medicine* 173: 614-622.

30. Hunt, P. W. 2012. HIV and inflammation: mechanisms and consequences. *Current HIV/AIDS reports* 9: 139-147.

31. Sankaran, S., M. D. George, E. Reay, M. Guadalupe, J. Flamm, T. Prindiville, and S. Dandekar. 2008. Rapid onset of intestinal epithelial barrier dysfunction in primary human immunodeficiency virus infection is driven by an imbalance between immune response and mucosal repair and regeneration. *Journal of virology* 82: 538-545.

32. Dinh, D. M., G. E. Volpe, C. Duffalo, S. Bhalchandra, A. K. Tai, A. V. Kane, C. A. Wanke, and H. D. Ward. 2015. Intestinal microbiota, microbial translocation, and systemic inflammation in chronic HIV infection. *The Journal of infectious diseases* 211: 19-27.

33. Ellis, C. L., Z. M. Ma, S. K. Mann, C. S. Li, J. Wu, T. H. Knight, T. Yotter, T. L. Hayes, A. H. Maniar, P. V. Troia-Cancio, H. A. Overman, N. J. Torok, A. Albanese, J. C. Rutledge, C. J. Miller, R. B. Pollard, and D. M. Asmuth. 2011. Molecular characterization of stool microbiota in HIV-infected subjects by panbacterial and order-level 16S ribosomal DNA (rDNA) quantification and correlations with immune activation. *Journal of acquired immune deficiency syndromes* 57: 363-370.

34. Dillon, S. M., E. J. Lee, C. V. Kotter, G. L. Austin, Z. Dong, D. K. Hecht, S. Gianella, B. Siewe, D. M. Smith, A. L. Landay, C. E. Robertson, D. N. Frank, and C. C. Wilson. 2014. An altered intestinal mucosal microbiome in HIV-1 infection is associated with mucosal and systemic immune activation and endotoxemia. *Mucosal immunology* 7: 983-994.

35. Mutlu, E. A., A. Keshavarzian, J. Losurdo, G. Swanson, B. Siewe, C. Forsyth, A. French, P. Demarais, Y. Sun, L. Koenig, S. Cox, P. Engen, P. Chakradeo, R. Abbasi, A. Gorenz, C. Burns, and A. Landay. 2014. A compositional look at the human gastrointestinal microbiome and immune activation parameters in HIV infected subjects. *PLoS pathogens* 10: e1003829.

36. Tunjungputri, R. N., A. J. Van Der Ven, A. Schonsberg, T. S. Mathan, P. Koopmans, M. Roest, R. Fijnheer, P. G. Groot, and Q. de Mast. 2014. Reduced platelet hyperreactivity and platelet-monocyte aggregation in HIV-infected individuals receiving a raltegravir-based regimen. *Aids* 28: 2091-2096.

37. O'Mahony, S. M., G. Clarke, Y. E. Borre, T. G. Dinan, and J. F. Cryan. 2015. Serotonin, tryptophan metabolism and the brain-gut-microbiome axis. *Behavioural brain research* 277: 32-48.

38. Dantzer, R., J. C. O'Connor, M. A. Lawson, and K. W. Kelley. 2011. Inflammation-associated depression: from serotonin to kynurenine. *Psychoneuroendocrinology* 36: 426-436.

39. Lovibond, P. F., and S. H. Lovibond. 1995. The structure of negative emotional states: comparison of the Depression Anxiety Stress Scales (DASS) with the Beck Depression and Anxiety Inventories. *Behaviour research and therapy* 33: 335-343.

40. Patton, J. H., M. S. Stanford, and E. S. Barratt. 1995. Factor structure of the Barratt impulsiveness scale. *Journal of clinical psychology* 51: 768-774.

41. Schippers, G. M., T. G. Broekman, and A. Bucholz. 2007. *MATE 2.0. Handleiding en protocol*. Betaboeken, Nijmegen.

42. Group, N. H. W., J. Peterson, S. Garges, M. Giovanni, P. McInnes, L. Wang, J. A. Schloss, V. Bonazzi, J. E. McEwen, K. A. Wetterstrand, C. Deal, C. C. Baker, V. Di Francesco, T. K. Howcroft, R. W. Karp, R. D. Lunsford, C. R. Wellington, T. Belachew, M. Wright, C. Giblin, H. David, M. Mills, R. Salomon, C. Mullins, B. Akolkar, L. Begg, C. Davis, L. Grandison, M. Humble, J. Khalsa, A. R. Little, H. Peavy, C. Pontzer, M. Portnoy, M. H. Sayre, P. Starke-Reed, S. Zakhari, J. Read, B. Watson, and M. Guyer. 2009. The NIH Human Microbiome Project. *Genome research* 19: 2317-2323.

43. Smeekens, S. P., A. Ng, V. Kumar, M. D. Johnson, T. S. Plantinga, C. van Diemen, P. Arts, E. T. Verwiel, M. S. Gresnigt, K. Fransen, S. van Sommeren, M. Oosting, S. C. Cheng, L. A. Joosten, A. Hoischen, B. J. Kullberg, W. K. Scott, J. R. Perfect, J. W. van der Meer, C. Wijmenga, M. G. Netea, and R. J. Xavier. 2013. Functional genomics identifies type I interferon pathway as central for host defense against Candida albicans. *Nature communications* 4: 1342.
