# Supplementary material for: 11-deoxycortisol positively correlates with T cell immune traits in physiological conditions
Source: eBioMedicine. 2023 Dec 21;99:104935. doi: 10.1016/j.ebiom.2023.104935 (PMC10776925; doi:10.1016/j.ebiom.2023.104935)
Supplement: 300BCG study protocol [file mmc3.pdf]

Understanding variability of immune responses to BCG vaccination: a systems biology approach

October 2016

PROTOCOL TITLE:

Understanding variability of immune responses to BCG vaccination: a systems biology approach

**Protocol ID**	**300BCG**
**Short title**	**Determinants of the response to BCG vaccine**
**Version**	**4**
**Date**	**17-10-2016**
**Coordinating investigator/ project leader**	***S.J.C.F.M. Moorlag, MSc, MD***<br>*Simone.Moorlag@radboudumc.nl*<br>***024 36 67218***<br>***Radboudumc, Department of Experimental Internal Medicine, 6525 GA, Nijmegen***
**Principal investigator(s) (in Dutch: hoofdonderzoeker/ uitvoerder)**	***Prof. Dr. M.G. Netea***<br>*mihai.netea@radboudumc.nl*<br>***024 36 14652***<br>***Radboudumc, Department of Experimental Internal Medicine, 6525 GA Nijmegen***
**Sponsor (in Dutch: verrichter/opdrachtgever)**	***Division of Internal Medicine, Radboudumc, Nijmegen, The Netherlands***
**Subsidising party**	
**Independent expert (s)**	**Dr. Q. De Mast, internist-infectiologist**
**Laboratory sites**	***Department of Experimental Internal Medicine, Route 462***<br><br>***Radboudumc***<br>***Postbus 9101***<br>***6500 HB Nijmegen***<br>***0243616345***
**Pharmacy**	***Centrale apotheek Radboudumc***

PROTOCOL SIGNATURE SHEET

**Name**	**Signature**	**Date**
**Head of Department:**<br>***Prof. Dr. J. Smit***		
**[Coordinating Investigator/Project leader/Principal Investigator]:**<br>***S.J.C.F.M. Moorlag, physician-researcher***		

TABLE OF CONTENTS

1. INTRODUCTION AND RATIONALE 10
2. OBJECTIVES 13
3. STUDY DESIGN 14
4. STUDY POPULATION 16
   4.1 Population (base) 16
   4.2 Inclusion criteria 16
   4.3 Exclusion criteria 16
   4.4 Sample size calculation 16
5. TREATMENT OF SUBJECTS 17
   5.1 Investigational product/treatment 17
   5.2 Use of co-intervention 17
6. INVESTIGATIONAL PRODUCT 18
   6.1 Name and description of investigational product(s) 18
   6.2 Summary of findings from non-clinical studies 18
   6.3 Summary of findings from clinical studies 18
   6.4 Summary of known and potential risks and benefits 19
   6.5 Description and justification of route of administration and dosage 19
   6.6 Dosages, dosage modifications and method of administration 19
   6.7 Preparation and labelling of Investigational Medicinal Product 19
   6.8 Drug accountability 19
7. METHODS 20
   7.1 Study parameters/endpoints 20
      7.1.1 Main study parameter/endpoint 20
      7.1.2 Other study parameters 20
   7.2 Study procedures 21
   7.3 Withdrawal of individual subjects 22
   7.4 Replacement of individual subjects after withdrawal 22
   7.5 Follow-up of subjects withdrawn from treatment 22
   7.6 Premature termination of the study 22
8. SAFETY REPORTING 23
   8.1 Temporary halt for reasons of subject safety 24
   8.2 AEs, SAEs and SUSARs 23
      8.2.1 Adverse events (AEs) 23
      8.2.2 Serious adverse events (SAEs) 23
      8.2.3 Suspected unexpected serious adverse reactions (SUSARs) 24
   8.3 Annual safety report 25
   8.4 Follow-up of adverse events 25
   8.5 [Data Safety Monitoring Board (DSMB) / Safety Committee] 25
9. STATISTICAL ANALYSIS 26
   9.1 Primary study parameter(s) 26
10. ETHICAL CONSIDERATIONS 28

10.1 Regulation statement 28
10.2 Recruitment and consent.. 28
10.3 Benefits and risks assessment, group relatedness 28
10.4 Compensation for injury 29
10.5 Incentives (if applicable) 29
11. ADMINISTRATIVE ASPECTS, MONITORING AND PUBLICATION 30
11.1 Handling and storage of data and documents 30
11.2 Monitoring and Quality Assurance 30
11.3 Amendments 31
11.4 Annual progress report 31
11.5 Temporary halt and (prematurely) end of study report 31
11.6 Public disclosure and publication policy 31
12. STRUCTURED RISK ANALYSIS 32
12.1 Synthesis 32
13. REFERENCES 32

LIST OF ABBREVIATIONS AND RELEVANT DEFINITIONS

ABR	ABR form, General Assessment and Registration form, is the application form that is required for submission to the accredited Ethics Committee (In Dutch, ABR = Algemene Beoordeling en Registratie)
AE	Adverse Event
AR	Adverse Reaction
BCG	Bacillus Calmette-Guérin
CA	Competent Authority
CCMO	Central Committee on Research Involving Human Subjects; in Dutch: Centrale Commissie Mensgebonden Onderzoek
CV	Curriculum Vitae
DSMB	Data Safety Monitoring Board
ELISA	Enzyme-linked immunosorbent assay
EU	European Union
EudraCT	European drug regulatory affairs Clinical Trials
FACS	Fluorescence-activated cell sorting
GCP	Good Clinical Practice
IB	Investigator's Brochure
IC	Informed Consent
IMP	Investigational Medicinal Product
IMPD	Investigational Medicinal Product Dossier
METC	Medical research ethics committee (MREC); in Dutch: medisch ethische toetsing commissie (METC)
PBMC	Peripheral blood mononuclear cell
(S)AE	(Serious) Adverse Event
SNP	Single nucleotide polymorphism
SPC	Summary of Product Characteristics (in Dutch: officiële productinfomatie IB1-tekst)
Sponsor	The sponsor is the party that commissions the organisation or performance of the research, for example a pharmaceutical company, academic hospital, scientific organisation or investigator. A party that provides funding for a study but does not commission it is not regarded as the sponsor, but referred to as a subsidising party.
SUSAR	Suspected Unexpected Serious Adverse Reaction
TB	Tuberculosis

**Wbp**	**Personal Data Protection Act (in Dutch: Wet Bescherming Persoonsgevens)**
**WMO**	**Medical Research Involving Human Subjects Act (in Dutch: Wet Medisch-wetenschappelijk Onderzoek met Mensen**

SUMMARY

Rationale: The Bacillus Calmette-Guérin (BCG) vaccine not only protects against infection with *Mycobacterium tuberculosis* and *Mycobacterium leprae*, but has also been shown to induce protection against a large number of unrelated pathogens. The non-specific effects of BCG lead to significant reduced infant morbidity and mortality. These striking effects are most likely mediated by the enhanced release of monocyte-derived cytokines resulting from epigenetic reprogramming of innate immune cells by BCG, a process that has been called trained immunity. However, the factors that influence the individual response to BCG vaccination remain largely unknown. A better understanding of the mechanisms involved is crucial in order to find ways to enhance innate immunological memory and could lead to the development of new vaccines and therapeutics.
Objective: To elucidate the host and environmental factors that influence the magnitude of the individual trained immunity responses to BCG vaccination using a systems biology approach.
Study design: Intervention trial.
Study population: 300 Healthy adult volunteers.
Intervention: Healthy adult volunteers will be vaccinated with BCG vaccine.
Main study parameters/endpoints:
1) E*x vivo* cytokine production by immune cells before and after vaccination upon stimulation with different pathogens will be measured by ELISA.
2) Immune cell subpopulations will be assessed by FACS.
3) Genetic variations that influence training will be assessed in DNA samples by Human Core SNP array from Illumina.
4) Epigenetic, transcription and metabolomic profiles before and after vaccination will be compared.
5) The correlation between the microbiome and the immune responses will be assessed.
6) The relationship between trained immunity and other host factors such as age, sex and metabolic state will be investigated.
7) The impact of environmental factors (e.g. season, stress) on induction of adaptive and innate immunity will be studied.

Nature and extent of the burden and risks associated with participation, benefit and group relatedness: Currently there is no known direct benefit to the study participants. The risks are negligible. Vaccination could cause pain, scarring at the site of injection, headache and fever. Venipuncture could result in minor discomfort and local hematoma formation could occur at the site of the blood drawing. Experienced personnel will be used in order to

minimize the minor side-effects. Volunteers have to spend 3 short visits and each visit they are asked to fill in a questionnaire and to donate a certain amount of blood.

1. INTRODUCTION AND RATIONALE

The most widely used vaccine in the world is the live attenuated Bacillus Calmette-Guérin (BCG) vaccine, which is recommended by the WHO in all areas with a high incidence of tuberculosis (TB). However, vaccination with BCG has significant effects on health beyond TB, coined 'non-specific effects'. One well known example of these effects is the ability of BCG to improve disease outcome in patients with bladder cancer (Sylvester, van der, & Lamm, 2002). Strikingly, in several epidemiological studies it was shown that immunization of neonates with BCG vaccine leads to a 50% reduction of neonatal mortality in low-income countries. This observed effect is probably due to the fact that already within three days after vaccination with BCG, children are less likely to develop sepsis and respiratory infections (Garly et al., 2003; Roth, Garly, Jensen, Nielsen, & Aaby, 2006).
Two possible mechanisms have been proposed as an explanation of the beneficial non-specific effects of BCG. First, BCG has been shown to enhance the adaptive immune response to unrelated antigens, leading to an increased immune response to other vaccinations up to one year after BCG vaccination (Kleinnijenhuis et al., 2014; Leentjens et al., 2015; Ota et al., 2002). However, the key mechanism by which BCG reduces overall mortality is probably by altering innate immunity. Immunological studies performed by our group have shown that BCG induces an increased immune response to secondary infections through epigenetic reprogramming of innate immune cells. More specific, results demonstrate increased H3K4 trimethylation at the promoter site of certain inflammatory genes in monocytes, resulting in enhanced pro-inflammatory responses to unrelated pathogens for up to 3 months after vaccination (Buffen et al., 2014; Jensen et al., 2015; Kleinnijenhuis et al., 2014). The term 'trained immunity' describes these memory properties of the innate immune system (Netea, Quintin, & van der Meer, 2011).

Although the discovery that epigenetic changes are responsible for innate immune memory has greatly improved our understanding of trained immunity, the factors that influence individual variability to BCG vaccination remain largely unknown. By identifying factors that correlate with adaptive and trained immunity by BCG, we will take an important step to understand individual variability to vaccination and design approaches for personalized vaccination. An increased understanding of trained immunity is crucial in order to control and enhance innate immune memory, which might lead to a complete new class of vaccines and therapeutics.

In this study we will adopt an interdisciplinary approach using immune phenotyping by multiplex cytokine analysis, flow cytometry and genome, transcriptome and metabolome profiling to characterize responses involved in adaptive and trained immunity after BCG vaccination. This systematic study enables us to study several factors that have been demonstrated to date to strongly influence the immune system and the immune response to vaccines (Pulendran, 2014). The factors that will be investigated in the current study are: 1) host genetic variation, 2) the epigenome, transcriptome and metabolome, 3) the microbiome,

4) other host factors such as sex, age and metabolic state, and 5) environmental factors, including physical stress and season.

1) Host genetic variation

One of the key host factors that may account for inter-individual variability is the genetic make-up of the individual. Immunogenetics studies have already revealed single nucleotide polymorphisms (SNPs) in cytokine, cytokine receptor, and innate immune response genes that influence vaccine-induced immunity (Poland, Ovsyannikova, Jacobson, & Smith, 2007). Polymorphisms in genes also control trained immunity. Recently, we have demonstrated that SNPs in the autophagy genes *ATG2B* and *ATG5* influence both the *in vitro* and *in vivo* training effect of BCG (Buffen et al., 2014). These findings revealed autophagy as a central event modulating trained immunity induced by BCG. Although future research is needed to identify the molecular mechanisms through which autophagy influences trained immunity, its discovery provides a better insight into the immunological process of training. Therefore, broadening our knowledge about DNA polymorphisms that effect training induced by BCG will be of great value in deciphering the pathways involved.

2) Epigenome, Transcriptome, Metabolome

DNA modifications that do not alter the DNA sequence but do affect gene activity are known as epigenetic changes. As mentioned earlier, it has been shown that epigenetic changes mediate trained immunity. In this study we will compare epigenomic modifications before and after vaccination and we will study the duration of these changes. In addition we will compare transcription profiles, which reflects the genes that are actively expressed at any given moment. Furthermore, we will study metabolomics as emerging evidence has suggested a very strong relationship between the immune and the metabolic system (Arts, Gresnigt, Joosten, & Netea, 2016; Hotamisligil, 2006). Because the metabolome is the final downstream product of gene transcription, it is closest to the phenotype of the cell. Moreover, it was demonstrated that inhibition of specific metabolic pathways blocks monocyte induction of trained immunity (Cheng et al., 2014). This underlines the key role for metabolism in trained immunity and further research into metabolite profiles is warranted

3) The Microbiome

Next to differences in genome, transcriptome and metabolome, the unique composition of microorganisms in the gut may also be able to impact training by BCG. The complex community of microorganisms on body surfaces such as the gut and the skin is called the microbiome. It has been demonstrated that the presence of certain classes of microorganisms can influence cytokine production and even disease outcome (Plantinga et al., 2011; Stappers et al., 2012). Both autoinflammatory disorders as well as infections have been related to microbial components present in the human body. Currently, it is not known if there is also a link between the microbiome and the induction of adaptive immune memory and trained immunity by BCG. In this study we will investigate if differences in microbial status correlate with the strength of these immune responses.

4) Other host and environmental factors

Besides ‘omic’ sciences and the microbiome, other host factors such as sex, age, substance abuse and the metabolic state of individuals are able to exert major influences on the immune system (Duraisingham et al., 2013; Hotamisligil, 2006). For example, it was found that sex-differential effects for almost all routine vaccinations exist. The RTS,S malaria vaccine as well as the DTP-containing pentavalent vaccine have negative nonspecific effects in girls, resulting in an higher all-cause mortality compared to boys (Aaby et al., 2003; Klein, Shann, Moss, Benn, & Aaby, 2016). Studies suggest that the beneficial non-specific effects of BCG on infant mortality are often more pronounced among girls (Roth et al., 2006). Furthermore, environmental factors may impact immunity. Diet, psychological stress, and allergies may all represent variables influencing immune responses to vaccination. Also seasonal differences in vaccine responses have been described (Hansen et al., 2014). It is currently not known if and to what extent any of these host and environmental factors influence training induced by BCG in adults.

In conclusion, despite all the research on factors that influence the immune system, very little is known about the effect they may have on adaptive immune memory and trained immunity induced by BCG vaccination. Therefore, we will initiate a systems biology approach to integrate pathway analysis of DNA polymorphisms, the epigenome, transcriptome and metabolome, the microbiome and other host and environmental factors that may modulate the nonspecific effects induced by BCG. The results of this study will be critical in providing a better understanding of the mechanisms underlying trained immunity. This in turn will identify the pathways that we could tackle in order to boost innate defense mechanisms through trained immunity induction. This could be in particular appealing for vulnerable populations, such as elderly people, that show a decreased resistance to pathogens. Moreover, results may show how to improve the induction of protection by BCG-based and other vaccines against infections and perhaps even against malignancies.

2. OBJECTIVES

Primary objective:

The main aim of this study is to determine which host- and environmental factors influence adaptive immune memory and trained immunity induced by BCG vaccination.

Specific research questions:

1. What is the variation of adaptive and trained immunity memory in a cohort of healthy volunteers after vaccination with BCG?
2. Which genetic polymorphisms modulate adaptive and trained immunity induced by BCG vaccination?
3. In which way does trained immunity alter the epigenome, transcriptome and metabolome of the host monocytes and do these changes correlate with trained immunity?
4. Is there a correlation between differences in the composition of the microbiome and the induction of adaptive memory and trained immunity?
5. Is the induction of trained immunity influenced by non-genetic host factors such as sex, age, substance abuse and metabolic state?

Secondary objectives:

6. Investigate whether environmental factors such as diet, psychological stress and season influence the ability to induce adaptive immunity and trained immunity by BCG vaccination.
7. Investigate if local skin inflammation and development of vaccination scar at the vaccination site 2 weeks after BCG vaccination correlate with induction of trained immunity.
8. Investigate whether the capacity of monocytes to display trained immunity in *in vitro* experiments correlates with trained immunity *in vivo*.

3. STUDY DESIGN

The intervention trial will be performed at the Radboudumc. 300 Healthy volunteers (equal numbers of females and males) will be recruited to receive a vaccination with BCG. After screening and obtaining informed consent, blood will be drawn by venipuncture before and 2 weeks and 3 months after vaccination as depicted below. The total duration of the study will be 2 years.

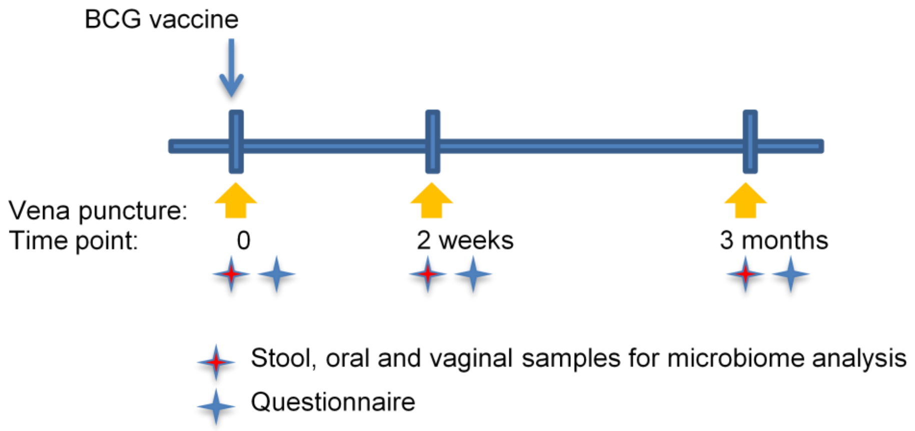

In order to identify new molecular pathways responsible for trained immunity, we will make use of an ‘omics’ based approach. Genomic information will be obtained using a SNP whole-genome SNP array from Illumina. Epigenomic, transcriptomic, and metabolomic data will be obtained from monocytes isolated before and after vaccination with BCG. In addition, cytokine assays will be performed on stimulated cells, and flow cytometry (FACS) will be used to assess immune cell populations in the circulation.

3.1 Determining function of circulating immune cells

Ex vivo stimulation experiments will assess cytokine productions by immune cells:

i. The *ex vivo* immune response will be determined in purified monocytes and PBMCs stimulated for 24 hours with RPMI (control), LPS, *Candida albicans*, *Mycobacterium tuberculosis* and *Staphylococcus aureus*. Time points: before vaccination, and 2 weeks and 3 months after vaccination.
At t=0 blood will be collected for 1) *in vitro* training experiments, and 2) direct stimulation experiments.

ii. Concentrations of various cytokines (Monocyte-derived and Th1/Th17-derived) will be measured in supernatants by ELISA.

3.2 Genomics

DNA samples will be obtained from EDTA blood collected from all healthy individuals. After isolation, DNA samples will be sequenced and genetic variations (SNPs) will be determined by a whole-genome SNP array from Illumina (collaboration group Prof. C. Wijmenga, UMC Groningen).

3.3 Epigenomics, transcriptomics and metabolomics
ChIP-PCR analysis will be used to measure enrichment of H3K4me3 and H3K9me3 at the promoter of *TNFA*, *IL6* and *IL1B* in human monocytes isolated from subjects before and after (2 weeks and 3 months) BCG vaccination**.** Epigenetic changes in monocytes as well as changes in mRNA and metabolites will be measured in all blood samples before and after vaccination.

3.4 Microbiome analysis
Volunteers will be asked to collect gut, oral and vaginal microbiome samples before BCG vaccination and two weeks and 3 months after vaccination. Microbiome analysis will be performed using shotgun sequencing metagenomics. All samples will be stored at -80°C until analysis.

3.5 Questionnaires
At all time points of blood collection, participants are asked to complete a questionnaire addressing questions on: current or previous infections (flu, fever, cold), substance abuse, perceived physical stress, medication (NSAIDs, aspirin), vaccination history (e.g. flu vaccine) etcetera.

Vaccinations will be performed at the outpatient clinic of the Department of Internal Medicine Radboudumc, and the experimental studies will all be performed at the laboratory of Experimental Internal Medicine at the Radboudumc.

4. STUDY POPULATION

4.1 Population (base)

Healthy volunteers (≥ 18 years old) of Western European ethnic background will be actively recruited using flyers and posters. The gender distribution of the cohort should be around 50% males and 50% females.

4.2 Inclusion criteria

In order to be eligible to participate in this study, a subject must meet all of the following criteria:

- Age ≥ 18 years
- No known chronic diseases, no acute infection at the time of vaccination
- Written informed consent

4.3 Exclusion criteria

A potential subject who meets any of the following criteria will be excluded from participation in this study:

- Use of chronic or acute medication during the last month before the study other than oral anti-contraceptive drugs
- Vaccination within 3 months prior to study period (subjects cannot be vaccinated with other vaccines during the study)
- Medical history of disease associated with immune deficiency
- Previous BCG vaccination
- Contact with tuberculosis patients or born in a tuberculosis endemic country
- Acute (febrile) illness within 4 weeks prior to start of study
- Pregnancy

4.4 Sample size calculation

The sample size calculation is not possible due to the variable frequencies of various traits in this study, such as SNP frequency and the variable prevalence of the microorganism classes in the microbiome. The size calculation will be variable depending on which type of polymorphism will be analyzed. The frequencies are not known and therefore power calculations are not possible to perform. Because our aim is to find new DNA polymorphisms that modulate training we need a larger sample size than the *in vivo* BCG studies that have been performed to date. To be able to detect any potential relevant SNPs, we decided to include 300 individuals, based on previous studies within the Human Functional Genomics Project.

5. VACCINATION OF SUBJECTS

5.1 Investigational product/treatment

Subjects in this study will be vaccinated with BCG vaccine (InterVax, Canada) using the standard vaccination technique for this vaccine, which is intradermal injection in the left upper arm. This vaccine is distributed by the Rijksinstituut voor Volksgezondheid en Milieu (RIVM) under approval of the Ministery of VWS and meets the requirements of WHO for dried BCG vaccine.

5.2 Use of co-intervention

Subjects are not allowed to use any medication; an exception is made for oral anticonception.

6. INVESTIGATIONAL PRODUCT

6.1 Name and description of investigational product

Bacille Calmette-Guérin is a live attenuated strain of *Mycobacterium bovis* developed in 1921 to prevent tuberculosis and other mycobacterial related infections. It is the most widely administered vaccine in the world and up to three billion individuals have received the vaccine. BCG is mainly used in countries with a high prevalence of tuberculosis to prevent childhood tuberculous meningitis and miliary disease. Although the vaccine primarily protects against tuberculosis, the vaccine is also effective for protection against leprosy and diseases related to nontuberculous mycobacteria. The BCG vaccine used in this study has been selected by the RIVM for use in the Netherlands (under approval of the Ministry of VWS) and is delivered by InterVax (Toronto, Canada) to the RIVM. The vaccine is registered in Bulgaria and produced by BulBio National Center of Infectious and Parasitic Diseases, BB-NCIPD Ltd. (Sofia, Bulgaria). The vaccine meets all the requirements of WHO for dried BCG vaccine (Requirements for Biological Substances No 11, formulated by WHO Expert Committee of Biological Standardization, Technical Report Series, No 745, 1987; 771, 1988).
Package contains 1 ampoule of lyophilized powder (0.5 mg) and 1 ampoule of diluent (1 ml, 0.9% NaCl). After resuspension of lyophilized vaccine in diluent, the vial contains 0.5 mg/ml. According to standard vaccination practices, participants will be vaccinated with 0.1 ml of injection suspension.

6.2 Summary of findings from non-clinical studies

In vitro experiments with isolated monocytes have demonstrated that BCG is able to induce innate immune memory. If monocytes are incubated with BCG for 24 hours and stimulated 7 days later with unrelated fungal and bacterial pathogens, this will result in a twofold enhanced release of monocyte-derived cytokines, such as IL-6, TNF-α and IL-1β, when compared to non-BCG trained cells. The ability to train monocytes is induced through the NOD2 receptor and mediated by epigenetic reprogramming (Kleinnijenhuis et al., 2012).

6.3 Summary of findings from clinical studies

BCG vaccine is the most used human vaccine in the world, with an excellent track record of safety. It mainly protects against severe forms of tuberculosis (meningitis, milliary TB), while providing only a limited protection against pulmonary TB. In addition, epidemiological studies have demonstrated that BCG vaccination reduces infant mortality independent of its effect on tuberculosis, which suggests that BCG might provide nonspecific protection against unrelated infections (Shann, 2013). *In vivo* studies with BCG vaccine performed by our research group have shown that BCG induces training of the innate immune system. The enhanced function of human monocytes persists for at least 3 months after vaccination, some of the protective effects have even been shown to last up to 1 year (Kleinnijenhuis et al., 2012).

6.4 Summary of known and potential risks and benefits

Apart from protection against extrapulmonary infection with *Mycobacterium tuberculosis* and *Mycobacterium leprae*, there are no expected benefits for participants in the study.
Potential risks include only the side effects of the vaccine, of which localized skin reactions are most common. The local reaction after BCG vaccination is usually mild and self-limiting. Less common are fever and headache after vaccination and if they occur they are typically mild. Enlargement of the axillary lymph nodes may occasionally occur after vaccination but will usually regress spontaneously after a few months.
After vaccination, seroconversion to Mantoux testing has been described to occur in 41.8% of the individuals, which decreases to 21.2% after 10 years (Farhat, Greenaway, Pai, & Menzies, 2006). For these reasons Mantoux testing cannot be used as a diagnostic test for tuberculosis in the future. However, other diagnostic tools are available such as the Quantiferon test and chest X-ray.
Local haematoma formation could occur at the site of vena puncture. See also the summary of product characteristics for a list of side effects.

6.5 Description and justification of route of administration and dosage

Reconstituted BCG vaccine should be given intradermally. According to standard vaccination practices, the dose for adults is 0.1 ml (see summary of product characteristics).

6.6 Dosages, dosage modifications and method of administration

0.1 ml of reconstituted BCG vaccine containing 0.050 mg of BCG will be administered intradermally in the left upper arm as recommended by the manufacturer (see summary of product characteristics).

6.7 Preparation and labelling of Investigational Medicinal Product

BCG vaccine will be purchased from the Clinical Pharmacy of the Radboudumc. Vaccines will be labeled according to GMP. Storage and preparation of vaccines will be done by experienced researchers.

6.8 Drug accountability

BCG vaccine will be purchased from the Clinical Pharmacy of the Radboudumc. Vaccines will be stored at 4°C in a room with temperature registration through the Xiltrix system. Vaccines will be kept in a dedicated box, labeled according to GMP. No other medication will be kept here. Issue of vaccines will be monitored on a drug accountability sheet.

7. METHODS

7.1 Study parameters/endpoints

7.1.1 Main study parameter/endpoint

According to the objectives:

<u>1) Assessment of immune cell function</u>

a. Stimulation Assays

Before BCG vaccination and at two different time points after vaccination (2 weeks and 3 months) blood will be drawn from which monocytes will be isolated by density centrifugation over Ficoll-Paque.

Cells will be restimulated *ex vivo* with different stimuli:

- control culture medium (RPMI)
- *E. coli* LPS (10ng/ml)
- *M. tuberculosis* 1x10^6 microorganisms/ml
- *C. albicans* 1x10^6 microorganisms/ml
- *S. aureus* 1x10^6 microorganisms/ml

b. Cytokine Measurements

Cytokine production will be assessed with ELISA after 24h (IL-6, TNFα, IL1-β) or 7 days (IFNγ, IL-17, IL-22) incubation. The cytokine production at two different time points after vaccination will be compared with the response before vaccination.

c. Immunophenotyping

Immunophenotyping of cell subpopulations using myeloid cell and lymphocyte protocols will be done using FACS analysis.

2) Genomics

Genetic variation will be assessed by a genome-wide SNP array from Illumina.

3) Epigenomics, transcriptomics, metabolomics

Epigenetic changes by ChIP-PCR as well as differences in transcriptome (by RNA sequencing) and metabolome (perform at Metabolon) will be determined before and after vaccination.

4) Microbiome

Human microbiome variation will be measured by shotgun metagenomics in collaboration with the group of Prof. Ramnik Xavier (Broad Institute/MIT).

7.1.2 Other study parameters

5) Metadata

Metadata (clinical, environmental data) will be collected from all subjects using questionnaires at each visit.

6) Skin inflammation at vaccination site
The vaccination site will be examined two weeks after BCG vaccination. A picture will be taken and scar size and the presence of local inflammation will be documented by an independent researcher.

7.2 Study procedures

All experiments and handling of samples will be done according to GCP standards. Subjects will be actively recruited using flyers and posters. If they meet the in- and exclusion criteria they will be informed about the study by the investigator and they will receive documentation regarding the study. A pregnancy test will be performed for female volunteers. If volunteers that meet all inclusion and exclusion criteria want to participate in the study, informed consent is signed. A Mantoux test will not be part of the procedure since 1) participants from tuberculosis endemic countries are excluded from the studies, and 2) a Mantoux test itself could potentially influence the effects induced by BCG.

<u>Invasive procedures to be performed:</u>
Day 0 (visit 1): vena puncture: 2.5 ml PaxGene tube, 50 ml EDTA bloed, 8 ml serum
vaccination with BCG vaccine.
Day 14 (visit 2): vena puncture: 2.5 ml PaxGene tube, 50 ml EDTA bloed, 8 ml serum
Month 3 (visit 3): vena puncture: 2.5 ml PaxGene tube, 50 ml EDTA bloed, 8 ml serum

<u>Other procedures regarding subjects:</u>
1) Questionnaires
At all time points metadata will be collected using questionnaires. At the first visit this will be a more extensive list of questions regarding lifestyle, that will take about 20 minutes to complete. At all other time points the questionnaire will be shorter and will take about 5 minutes to complete. It addresses questions on current health status, level of physical stress etcetera (see the F1 questionnaire document for a complete overview).
2) Microbiome
Stool,oral and vaginal samples will be collected by the participants in three separate containers on day 0, day 14 and 3 months after vaccination.

<u>Laboratory techniques to be performed:</u>
Stimulation and cytokine assays: At all time points PBMC isolation will be performed and monocytes will be *ex vivo* stimulated with various pathogens. Cytokine production will be assessed after 24 hours and after 7 days with ELISA technique. Cytokine levels before and after vaccination will be compared. Immunophenotyping will be done using FACS analysis.

Genomics: At the first visit 3 ml of blood for DNA isolation of healthy volunteers will be stored.
Microbiome: Samples will be analysed by shotgun metagenome sequencing.

Epigenomics, transcriptomics metabolomics: Supernatant and cell lysates will be stored for epigenomics, transcriptomics and metabolomics studies.

7.3 Withdrawal of individual subjects

Subjects can leave the study at any time for any reason if they wish to do so without any consequences. The investigator can decide to withdraw a subject from the study for urgent medical reasons. Reasons for withdrawal will be documented.

7.4 Replacement of individual subjects after withdrawal

When subjects withdraw from the study they will be replaced in order to obtain a sample size of 300 subjects. The maximum number of drop outs that will be replaced is 50.

7.5 Follow-up of subjects withdrawn from treatment

If medically necessary, subjects will be monitored after withdrawal medically by the principal investigator.

7.6 Premature termination of the study

The BCG vaccine is considered to be a safe vaccine, therefore, no serious adverse events are expected. However, if there is sufficient ground that continuation of the study will jeopardise subject health or safety, the study will not be continued before the cause of the SAE is known and a further positive decision by the accredited METC is given.

8. SAFETY REPORTING

8.1 Temporary halt for reasons of subject safety

In accordance to section 10, subsection 4, of the WMO, the sponsor will suspend the study if there is sufficient ground that continuation of the study will jeopardise subject health or safety. The sponsor will notify the accredited METC without undue delay of a temporary halt including the reason for such an action. The study will be suspended pending a further positive decision by the accredited METC. The investigator will take care that all subjects are kept informed.

8.2 AEs, SAEs and SUSARs

8.2.1 Adverse events (AEs)

Adverse events are defined as any undesirable experience occurring to a subject during the study, whether or not considered related to the vaccination. All adverse events reported spontaneously by the subject or observed by the investigator or his staff will be recorded.

8.2.2 Serious adverse events (SAEs)

A serious adverse event is any untoward medical occurrence or effect that:

- results in death;
- is life threatening (at the time of the event);
- requires hospitalisation;
- results in persistent or significant disability or incapacity;
- is a congenital anomaly or birth defect; or
- any other important medical event that did not result in any of the outcomes listed above due to medical or surgical intervention but could have been based upon appropriate judgement by the investigator.

An elective hospital admission will not be considered as a serious adverse event.

As the BCG vaccine is considered to be safe, no SAEs are expected. However, in case of a SAE the condition of the volunteer will be evaluated by the independent MDs, who can decide to break the randomization code. If needed, the volunteer will be admitted to our hospital or will be evaluated by an independent MD at the outpatient clinic.

The investigator will report all SAEs to the sponsor without undue delay after obtaining knowledge of the events.

The sponsor will report the SAEs through the web portal *ToetsingOnline* to the accredited METC that approved the protocol, within 7 days of first knowledge for SAEs that result in death or are life threatening followed by a period of maximum of 8 days to complete the initial preliminary report. All other SAEs will be reported within a period of maximum 15 days after the sponsor has first knowledge of the serious adverse events.

8.2.3 Suspected unexpected serious adverse reactions (SUSARs)

Adverse reactions are all untoward and unintended responses to an investigational product related to any dose administered.

Unexpected adverse reactions are SUSARs if the following three conditions are met:

1. the event must be serious (see chapter 9.2.2);
2. there must be a certain degree of probability that the event is a harmful and an undesirable reaction to the medicinal product under investigation, regardless of the administered dose;
3. the adverse reaction must be unexpected, that is to say, the nature and severity of the adverse reaction are not in agreement with the product information as recorded in:
   - Summary of Product Characteristics (SPC) for an authorised medicinal product;
   - Investigator's Brochure for an unauthorised medicinal product.

The sponsor will report expedited the following SUSARs through the web portal *ToetsingOnline* to the METC *<reporting via webportalToetsingOnline is only applicable for investigator initiated studies>*:

- SUSARs that have arisen in the clinical trial that was assessed by the METC;
- SUSARs that have arisen in other clinical trials of the same sponsor and with the same medicinal product, and that could have consequences for the safety of the subjects involved in the clinical trial that was assessed by the METC.

The remaining SUSARs are recorded in an overview list (line-listing) that will be submitted once every half year to the METC. This line-listing provides an overview of all SUSARs from the study medicine, accompanied by a brief report highlighting the main points of concern.

The expedited reporting of SUSARs through the web portal Eudravigilance or ToetsingOnline is sufficient as notification to the competent authority.

The sponsor will report expedited all SUSARs to the competent authorities in other Member States, according to the requirements of the Member States.

The expedited reporting will occur not later than 15 days after the sponsor has first knowledge of the adverse reactions. For fatal or life threatening cases the term will be maximal 7 days for a preliminary report with another 8 days for completion of the report.

8.3 Annual safety report

In addition to the expedited reporting of SUSARs, the sponsor will submit, once a year throughout the clinical trial, a safety report to the accredited METC, competent authority, and competent authorities of the concerned Member States.
This safety report consists of:

- a list of all suspected (unexpected or expected) serious adverse reactions, along with an aggregated summary table of all reported serious adverse reactions, ordered by organ system, per study;
- a report concerning the safety of the subjects, consisting of a complete safety analysis and an evaluation of the balance between the efficacy and the harmfulness of the medicine under investigation.

8.4 Follow-up of adverse events

All AEs will be followed until they have abated, or until a stable situation has been reached. Depending on the event, follow up may require additional tests or medical procedures as indicated, and/or referral to the general physician or a medical specialist. SAEs need to be reported till end of study within the Netherlands, as defined in the protocol

8.5 [Data Safety Monitoring Board (DSMB) / Safety Committee]

As we classify this study as a ‘laag-risico onderzoek’, with a ‘verwaarloosbaar risico’, once a year an independent competent colleague will perform the monitoring according to the provided guidelines by the NFU.

9. STATISTICAL ANALYSIS

9.1 Primary study parameter(s)

The first step in the analysis of this study will be to examine the induction of adaptive immune memory and trained immunity in the cohort and to determine the variation between subjects. Inter-individual variation of the cytokine responses will be evaluated using Fligner-Killeen test of homogeneity of variances. The variance for each of the stimulation conditions will be compared to the unstimulated state.

Cytokine production upon *ex vivo* restimulation of monocytes before vaccination will be compared with cytokine levels after vaccination with BCG. Since every subject will be compared to themselves and cytokine production is considered to be non-parametric, the Wilcoxon signed rank test will be used in order to calculate the enhanced function of monocytes (trained immunity).

Results will be expressed as means ± standard errors of the means (SEM). P values less than 0,05 are considered statistically significant.

All tests will be performed on GraphPad Prism 6.0 software (GraphPad).

To determine and link genetic polymorphisms to trained immunity, genotype data will be analyzed using haploview software. The Wilcoxon signed rank test will be used in metabolomics and epigenomics studies in order to compare data before and after vaccination. Data validation will be essential to ensure that findings are not false positives.

Microbiome reads will be first processed using KneadData. This includes quality-trimming (trimmomatic parameters: MAXINFO:90:0.5), read-filtering based on a minimum read length of 60bp and removal of potential human contamination by filtering reads that aligned to the human genome (reference genome hg19). Quality-controlled, paired-end reads will be aligned against a database of unique clade-specific marker genes using bowtie2 and taxonomic profiles and inferred with MetaPhlAn 2.2 (Segata et al., 2012). For the subsequent analysis we will consider species as well as genus composition of the samples. Functional microbiome profiling will be done using HUMAnN2 (Segata et al., 2013). Briefly, reads will be mapped against a customized database of functionally annotated pangenomes only considering organisms that were identified during the taxonomic profiling step. Functional annotation of the protein-sequences in the pangenomes to their respective UniRef50 family will be provided.

Given the complexity and the amount of data that will be generated in this study, the statistical analyses will be performed in strong collaboration with bioinformaticians and systems biology experts from the group of Prof. C. Wijmenga (Department of Genetics,

UMCG) and of Prof. R. Xavier (Broad Institute at MIT/Harvard).

10 ETHICAL CONSIDERATIONS

10.1 Regulation statement

All experiments and handling of samples will be performed according to the latest version of the declaration of Helsinki (last amended at the 59th WMA General Assembly, Seoul, Korea, October 2008), the Medical Research Involving Human Subjects Act (WMO), and Good Clinical Practice.

10.2 Recruitment and consent

Healthy volunteers will be recruited by advertisements (e.g. flyers, posters) in Nijmegen, on bulletin boards at the university, via online bulletin boards and through presentations at the university. In addition, information about this study will be provided to participants of previous studies (e.g. the 500FG project) at our department that have given permission to be approached for future studies. Written information about the study will be provided and subjects have the opportunity to ask questions to the investigator. If subjects want to participate in the study, the main investigator will discuss the study with them and subjects can ask questions if they have any. In addition, an independent physician could provide answers to questions subjects might have. If subjects would like to participate in the study and they meet all the in- and exclusion criteria, informed consent will be signed and upcoming visits will be planned. At least one day will be taken into account before informed consent can be given. Please find attached as separate documents the patient information and informed consent form.

10.3 Benefits and risks assessment, group relatedness

The study population consists of healthy adults. Apart from protection against extrapulmonary infection with *Mycobacterium tuberculosis* and against leprosy, there are no expected benefits for participants in the study. However, findings may show new mechanisms responsible for the non-specific effects of vaccination and this may lead to novel strategies to optimize vaccination programs.

The risks to the subjects in this study are minimal. Potential risks include only the side effects of the vaccine, of which localized skin reactions are most common. The local reaction after BCG vaccination is usually mild and self-limiting. Less common are fever and headache after vaccination and if they occur they are typically mild. Enlargement of the axillary lymph nodes may occasionally occur after vaccination but will usually regress spontaneously after a few months.

No risks other than local hematoma and minor pain are related to venous puncture. Risks will be minimized since venipuncture will be performed by experienced personnel.

In conclusion, the potential risk and burden are proportional to the potential value of this study.

10.4 Compensation for injury

The sponsor/investigator has a liability insurance which is in accordance with article 7 of the WMO.

The sponsor (also) has an insurance which is in accordance with the legal requirements in the Netherlands (Article 7 WMO). This insurance provides cover for damage to research subjects through injury or death caused by the study.

The insurance applies to the damage that becomes apparent during the study or within 4 years after the end of the study.

10.5 Incentives

Participants in this study will receive 150 Euro (i.e. hundred fifty Euro) as compensation for the time spent in the hospital for study procedures. The BCG vaccine (about 35 euro) is given to the subjects free of charge and might benefit them in the future. A proportional share of the total fee will be given when a subject ends participation before the end of the study. When the reason for withdrawal is because of medical issues, the entire fee will be given.

11. ADMINISTRATIVE ASPECTS, MONITORING AND PUBLICATION

11.1 Handling and storage of data and documents

Data will be handled coded and confidentially. The main investigator will code samples and only he/she and the PI will have access to the data. The code list linking the subjects to the coded data/materials will be stored by the main investigator. The code will consists of the study code followed by SxVx, where 'S' will indicate the subject number and V the different time points. All study materials (e.g. supernatants, lysed cells, plasma, stool and oral samples) will be stored according to Good Clinical practice and in a completely coded way up to 15 years. Both the main investigator and the PI have access to stored material.

This study follows a systems biology approach. This means that there is the possibility to produce large datasets. The study materials will be stored up to 15 years in order to perform experiments on innate immune memory with the current but also with potentially new techniques. The purpose of this is to facilitate all analyses performed in order to elucidate the underlying mechanisms of trained immunity; such as measuring additional pro-inflammatory cytokines, RNA transcription etcetera. Hence, there will be a direct link with the current research proposal.

All materials will be stored in a locked freezer at the department of Internal Medicine of the Radboudumc. The materials will be managed by the Principal Investigator (PI), professor Mihai Netea. The PI is responsible for all materials and his permission is needed when there is a request for material transfer, which will be in an anonymous form. Assessment by an accredited medical research ethics committee is required before materials will be used for research that has no connections with the current subject of research and/or research in which there is a possibility to encounter ancillary findings.Volunteers will be asked for their permission to store and analyse materials after the indicated study period on the informed consent form. On the same form, volunteers are also asked for their permission to be approached after the study period for future studies or follow-up.

11.2 Monitoring and Quality Assurance

Monitoring of this study will be done in accordance with the monitoring plan, which is made in collaboration with the Clinical Research Centre Nijmegen.

11.3 Amendments

Amendments are changes made to the research after a favourable opinion by the accredited METC has been given. All amendments will be notified to the METC that gave a favourable opinion.

11.4 Annual progress report

The sponsor/investigator will submit a summary of the progress of the trial to the accredited METC once a year. Information will be provided on the date of inclusion of the first subject, numbers of subjects included and numbers of subjects that have completed the trial, serious adverse events/serious adverse reactions, other problems, and amendments.

11.5 Temporary halt and (prematurely) end of study report

The investigator/sponsor will notify the accredited METC of the end of the study within a period of 8 weeks. The end of the study is defined as the last patient’s last visit.
The sponsor will notify the METC immediately of a temporary halt of the study, including the reason of such an action.

In case the study is ended prematurely, the sponsor will notify the accredited METC within 15 days, including the reasons for the premature termination.

Within one year after the end of the study, the investigator/sponsor will submit a final study report with the results of the study, including any publications/abstracts of the study, to the accredited METC.

11.6 Public disclosure and publication policy

The study results, both positive and negative, will be submitted for publication to peer reviewed journals. The investigators will hold final responsibility for the decision to submit publications.

12. STRUCTURED RISK ANALYSIS

12.1 Synthesis

The risks for participation in this study are very low. BCG vaccine is the most widely used vaccine in the world, being part of vaccination policies in many countries. The side effects of the vaccine are very well known and the common side effects are minor and almost always self-limiting. Major side effects are very rare in healthy people (see also summary of product characteristics). The BCG vaccine which will be used in the study has been selected by the RIVM for use in the Netherlands (under approval of the Ministry of VWS) and is delivered by InterVax (Toronto, Canada) to the RIVM. The vaccine is produced by BB-NCIPD Ltd (Sofia, Bulgaria) and is registered in Bulgaria. The vaccine has been recently approved by the CCMO for use in another study performed by our department (NL56222.01 9.15).
Because of the negligible risks for the healthy volunteers included in this study, we consider this trial a non-medicinal product trial.

13. REFERENCES

Aaby, P., Jensen, H., Samb, B., Cisse, B., Sodemann, M., Jakobsen, M., . . . Whittle, H. (2003). Differences in female-male mortality after high-titre measles vaccine and association with subsequent vaccination with diphtheria-tetanus-pertussis and inactivated poliovirus: reanalysis of West African studies. *Lancet, 361*(9376), 2183-2188. doi: 10.1016/s0140-6736(03)13771-3
Arts, R. J., Gresnigt, M. S., Joosten, L. A., & Netea, M. G. (2016). Cellular metabolism of myeloid cells in sepsis. *J Leukoc Biol*. doi: 10.1189/jlb.4MR0216-066R
Buffen, K., Oosting, M., Quintin, J., Ng, A., Kleinnijenhuis, J., Kumar, V., . . . Joosten, L. A. (2014). Autophagy controls BCG-induced trained immunity and the response to intravesical BCG therapy for bladder cancer. *PLoS Pathog, 10*(10), e1004485. doi: 10.1371/journal.ppat.1004485
Cheng, S. C., Quintin, J., Cramer, R. A., Shepardson, K. M., Saeed, S., Kumar, V., . . . Netea, M. G. (2014). mTOR- and HIF-1alpha-mediated aerobic glycolysis as metabolic basis for trained immunity. *Science, 345*(6204), 1250684. doi: 10.1126/science.1250684
Duraisingham, S. S., Rouphael, N., Cavanagh, M. M., Nakaya, H. I., Goronzy, J. J., & Pulendran, B. (2013). Systems biology of vaccination in the elderly. *Curr Top Microbiol Immunol, 363*, 117-142. doi: 10.1007/82_2012_250
Farhat, M., Greenaway, C., Pai, M., & Menzies, D. (2006). False-positive tuberculin skin tests: what is the absolute effect of BCG and non-tuberculous mycobacteria? *Int J Tuberc Lung Dis, 10*(11), 1192-1204.
Garly, M. L., Martins, C. L., Bale, C., Balde, M. A., Hedegaard, K. L., Gustafson, P., . . . Aaby, P. (2003). BCG scar and positive tuberculin reaction associated with reduced child mortality in West Africa. A non-specific beneficial effect of BCG? *Vaccine, 21*(21-22), 2782-2790.
Hansen, A. S., Lund, N., Flanagan, K. L., Rodrigues, A., Njie-Jobe, J., Sanyang, L. C., . . . Whittle, H. (2014). Randomized trial: The effect of oral polio vaccine at birth on polio antibody titers at 6 weeks and 6 months of age. *Trials in Vaccinology, 3*, 33-39. doi:
Hotamisligil, G. S. (2006). Inflammation and metabolic disorders. *Nature, 444*(7121), 860-867. doi: 10.1038/nature05485
Jensen, K. J., Larsen, N., Biering-Sorensen, S., Andersen, A., Eriksen, H. B., Monteiro, I., . . . Benn, C. S. (2015). Heterologous immunological effects of early BCG vaccination in

low-birth-weight infants in Guinea-Bissau: a randomized-controlled trial. *J Infect Dis, 211*(6), 956-967. doi: 10.1093/infdis/jiu508
Klein, S. L., Shann, F., Moss, W. J., Benn, C. S., & Aaby, P. (2016). RTS,S Malaria Vaccine and Increased Mortality in Girls. *MBio, 7*(2). doi: 10.1128/mBio.00514-16
Kleinnijenhuis, J., Quintin, J., Preijers, F., Benn, C. S., Joosten, L. A., Jacobs, C., . . . Netea, M. G. (2014). Long-lasting effects of BCG vaccination on both heterologous Th1/Th17 responses and innate trained immunity. *J Innate Immun, 6*(2), 152-158. doi: 10.1159/000355628
Kleinnijenhuis, J., Quintin, J., Preijers, F., Joosten, L. A., Ifrim, D. C., Saeed, S., . . . Netea, M. G. (2012). Bacille Calmette-Guerin induces NOD2-dependent nonspecific protection from reinfection via epigenetic reprogramming of monocytes. *Proc Natl Acad Sci U S A, 109*(43), 17537-17542. doi: 10.1073/pnas.1202870109
Leentjens, J., Kox, M., Stokman, R., Gerretsen, J., Diavatopoulos, D. A., van Crevel, R., . . . Netea, M. G. (2015). BCG Vaccination Enhances the Immunogenicity of Subsequent Influenza Vaccination in Healthy Volunteers: A Randomized, Placebo-Controlled Pilot Study. *J Infect Dis, 212*(12), 1930-1938. doi: 10.1093/infdis/jiv332
Netea, M. G., Quintin, J., & van der Meer, J. W. (2011). Trained immunity: a memory for innate host defense. *Cell Host Microbe, 9*(5), 355-361. doi: 10.1016/j.chom.2011.04.006
Ota, M. O., Vekemans, J., Schlegel-Haueter, S. E., Fielding, K., Sanneh, M., Kidd, M., . . . Marchant, A. (2002). Influence of Mycobacterium bovis bacillus Calmette-Guerin on antibody and cytokine responses to human neonatal vaccination. *J Immunol, 168*(2), 919-925.
Plantinga, T. S., van Maren, W. W., van Bergenhenegouwen, J., Hameetman, M., Nierkens, S., Jacobs, C., . . . Netea, M. G. (2011). Differential Toll-like receptor recognition and induction of cytokine profile by Bifidobacterium breve and Lactobacillus strains of probiotics. *Clin Vaccine Immunol, 18*(4), 621-628. doi: 10.1128/cvi.00498-10
Poland, G. A., Ovsyannikova, I. G., Jacobson, R. M., & Smith, D. I. (2007). Heterogeneity in vaccine immune response: the role of immunogenetics and the emerging field of vaccinomics. *Clin Pharmacol Ther, 82*(6), 653-664. doi: 10.1038/sj.clpt.6100415
Pulendran, B. (2014). Systems vaccinology: Probing humanity's diverse immune systems with vaccines. *Proceedings of the National Academy of Sciences, 111*(34), 12300-12306. doi: 10.1073/pnas.1400476111
Roth, A., Garly, M. L., Jensen, H., Nielsen, J., & Aaby, P. (2006). Bacillus Calmette-Guerin vaccination and infant mortality. *Expert Rev Vaccines, 5*(2), 277-293. doi: 10.1586/14760584.5.2.277
Segata, N., Boernigen, D., Tickle, T. L., Morgan, X. C., Garrett, W. S., & Huttenhower, C. (2013). Computational meta'omics for microbial community studies. *Molecular Systems Biology, 9*(1). doi: 10.1038/msb.2013.22
Segata, N., Waldron, L., Ballarini, A., Narasimhan, V., Jousson, O., & Huttenhower, C. (2012). Metagenomic microbial community profiling using unique clade-specific marker genes. *Nat Meth, 9*(8), 811-814. doi:
Shann, F. (2013). Nonspecific Effects of Vaccines and the Reduction of Mortality in Children. *Clinical Therapeutics, 35*(2), 109-114. doi:
Stappers, M. H., Janssen, N. A., Oosting, M., Plantinga, T. S., Arvis, P., Mouton, J. W., . . . Gyssens, I. C. (2012). A role for TLR1, TLR2 and NOD2 in cytokine induction by Bacteroides fragilis. *Cytokine, 60*(3), 861-869. doi: 10.1016/j.cyto.2012.08.019
Sylvester, R. J., van der, M. A., & Lamm, D. L. (2002). Intravesical bacillus Calmette-Guerin reduces the risk of progression in patients with superficial bladder cancer: a meta-analysis of the published results of randomized clinical trials. *J Urol, 168*(5), 1964-1970. doi: 10.1097/01.ju.0000034450.80198.1c
